# Functional network topology in drug resistant and well-controlled idiopathic generalized epilepsy: a resting state functional MRI study

**DOI:** 10.1093/braincomms/fcab196

**Published:** 2021-08-26

**Authors:** Emily J Pegg, Andrea McKavanagh, R Martyn Bracewell, Yachin Chen, Kumar Das, Christine Denby, Barbara A K Kreilkamp, Petroula Laiou, Anthony Marson, Rajiv Mohanraj, Jason R Taylor, Simon S Keller

**Affiliations:** 1 Department of Neurology, Manchester Centre for Clinical Neurosciences, Salford Royal NHS Foundation Trust, Salford, UK; 2 Division of Neuroscience and Experimental Psychology, School of Biological Sciences, Faculty of Biology, Medicine and Health, University of Manchester, Manchester, UK; 3 Department of Pharmacology and Therapeutics, Institute of Systems, Molecular and Integrative Biology, University of Liverpool, Liverpool, UK; 4 The Walton Centre NHS Foundation Trust, Liverpool, UK; 5 Department of Neurology, University Medical Center Göttingen, Göttingen, Germany; 6 Department of Biostatistics and Health Informatics, Institute of Psychiatry, Psychology and Neuroscience, King’s College London, London, UK; 7 Manchester Academic Health Sciences Centre, University of Manchester, Manchester, UK

**Keywords:** connectivity, epilepsy, networks, drug-resistance, fMRI

## Abstract

Despite an increasing number of drug treatment options for people with idiopathic generalized epilepsy (IGE), drug resistance remains a significant issue and the mechanisms underlying it remain poorly understood. Previous studies have largely focused on potential cellular or genetic explanations for drug resistance. However, epilepsy is understood to be a network disorder and there is a growing body of literature suggesting altered topology of large-scale resting networks in people with epilepsy compared with controls. We hypothesize that network alterations may also play a role in seizure control. The aim of this study was to compare resting state functional network structure between well-controlled IGE (WC-IGE), drug resistant IGE (DR-IGE) and healthy controls. Thirty-three participants with IGE (10 with WC-IGE and 23 with DR-IGE) and 34 controls were included. Resting state functional MRI networks were constructed using the Functional Connectivity Toolbox (CONN). Global graph theoretic network measures of average node strength (an equivalent measure to mean degree in a network that is fully connected), node strength distribution variance, characteristic path length, average clustering coefficient, small-world index and average betweenness centrality were computed. Graphs were constructed separately for positively weighted connections and for absolute values. Individual nodal values of strength and betweenness centrality were also measured and ‘hub nodes’ were compared between groups. Outcome measures were assessed across the three groups and between both groups with IGE and controls. The IGE group as a whole had a higher average node strength, characteristic path length and average betweenness centrality. There were no clear differences between groups according to seizure control. Outcome metrics were sensitive to whether negatively correlated connections were included in network construction. There were no clear differences in the location of ‘hub nodes’ between groups. The results suggest that, irrespective of seizure control, IGE interictal network topology is more regular and has a higher global connectivity compared to controls, with no alteration in hub node locations. These alterations may produce a resting state network that is more vulnerable to transitioning to the seizure state. It is possible that the lack of apparent influence of seizure control on network topology is limited by challenges in classifying drug response. It is also demonstrated that network topological features are influenced by the sign of connectivity weights and therefore future methodological work is warranted to account for anticorrelations in graph theoretic studies.

## Teaser

Pegg et al. report that, irrespective of seizure control, network topology in idiopathic generalized epilepsy is more regular and has higher global connectivity, compared with controls.

## Graphical Abstract

**Graphical Abstract:**
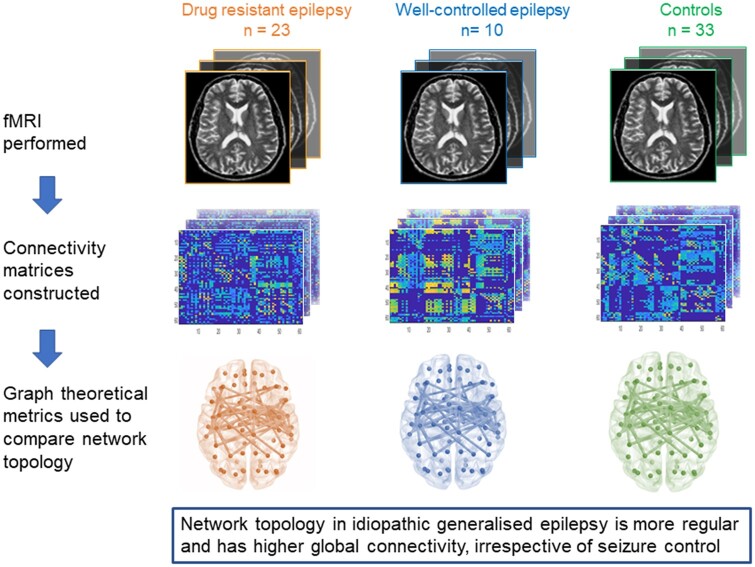

## Introduction

Epilepsy affects around 70 million people worldwide,^1^ of whom 15–20% are estimated to have idiopathic generalized epilepsy (IGE).^2^ IGEs comprise a group of syndromes characterized by the occurrence of generalized seizures in the absence of neurodevelopmental abnormalities or structural brain lesions.^3^ Approximately 18% of people with IGE do not become seizure-free despite an adequate trial of at least two appropriate and tolerated antiepileptic drugs (AEDs).^4^^,^^5^ Subsequent changes to drug regimens have a low chance of resulting in seizure freedom^6^ and, therefore, such patients are considered to have drug resistant epilepsy.^7^ In addition to a high seizure burden, people with drug resistant epilepsy have a higher rate of injury,^8^ sudden unexplained death in epilepsy,^9^ and social difficulties,^10^ compared with those with controlled seizures.

Traditionally, drug resistance in epilepsy has been examined from a cellular or genetic perspective. However, such approaches have failed to fully explain the underlying mechanisms of drug resistance.^11^ Since epilepsy is now understood to be a network disorder, in which seizures emerge from the dynamic resting state of the brain,^12^ investigating epilepsy drug resistance from a resting state network perspective may facilitate greater understanding of this important issue.

Resting state brain networks may be examined using functional MRI (fMRI), whereby blood oxygen level dependent (BOLD) signal is statistically analysed to establish the extent of connectivity between regions. Graph theory provides a robust mathematical method to subsequently delineate and analyse network topology (structure). Within this framework, each brain area is termed a ‘node’ and the connections between nodes are termed ‘edges’. Edges may be weighted according to the strength of correlation of BOLD signal between nodes. Information regarding the presence and strength of pairs of connections within a network is contained within a connectivity matrix and from this, a range of network metrics and features can be determined (Table 1).^13^^,^^14^ Overall evidence from graph theoretical studies derived from electroencephalography (EEG), magnetoencephalography (MEG) and MRI suggests that networks of people with focal epilepsy and IGE have a more regular topology compared with controls.^15^^,^^16^ It has been proposed that this regularity may render the network more likely to synchronize than a network that has a more random structure.^17^ However, there are inconsistencies within the literature, with some studies consistent with a more random network structure in epilepsy and others not identifying any differences in network regularity.^18–20^

**Table 1:** Commonly used graph theoretical terms and measures applied to epilepsy research

Node (vertex) (*n*)	The unit which forms a graph and represents an underlying brain region
Edge	Connection between two nodes
Directed edge	Information flows in one direction only
Undirected edge	Information flows in either direction
Weighted edge	A value given to an edge according to the strength of the connection
Degree distribution variance/node strength distribution variance	The variance of the node degree/node strength distribution
Degree (*k*)	Number of connections of a node
Node strength	The summed strength of connections of a node in a fully connected network. This is an equivalent measure to the node degree.
	Nodes with a high number of connections or a high connectivity strength may be regarded as ‘hub nodes’
	The mean value of the node strength values of all network nodes
Average node strength	
Clustering coefficient (*C*)	The probability that the neighbouring nodes of a given node are themselves connected
	C is averaged to calculate the clustering coefficient of the whole graph. (A measure of network segregation)
Mean clustering coefficient (*Ci*)	
Path length (*d*)	Minimum (or shortest) number of edges connecting 2 nodes
	Mean of the shortest path length between all pairs of network nodes (a measure of network integration)
Characteristic path length (*L*)	
Small-worldness	Ratio of average clustering coefficient of the graph to the mean clustering coefficient of a similar size random graph as a proportion of the ratio of the characteristic path length of the graph compared with the path length of a random graph
	[C/C *random* ]
	[P/P *random*]
	Small-world networks have higher than expected clustering coefficient with a characteristic path length of equal or lower value than a random graph
Betweenness centrality	A measure of to what extent a node lies on all shortest paths between each pair of network nodes.
	A measure of the importance of a node within the network. Nodes with high betweenness centrality may be regarding as ‘hub nodes’
	The mean value of the betweenness centrality values of all network nodes
Average betweenness centrality	

To our knowledge, analysing fMRI-derived functional connectivity from a global network perspective in IGE according to seizure control has not previously been considered. However, reduced connectivity in specific networks (cerebellar and default mode networks) in drug resistant IGE (DR-IGE) compared with well-controlled IGE (WC-IGE) has been described.^21^^,^^22^ In an EEG topology study by our group, in a different patient cohort, differences were found between controls and WC-IGE in the 10–12 Hz frequency band (compared with controls, mean degree and degree distribution variance was lower in WC-IGE and small world index was higher).^23^ This perhaps suggests that in people who respond to medication, drug-induced alterations to the network render the network less susceptible to seizures.

Considering that network topology may play a role in seizure control in IGE, and that diverging findings in the literature of IGE network topology may be influenced by a lack of evaluation according to seizure control,^16^ the aim of this study was to compare resting state global network topology in people with DR-IGE, WC-IGE and controls, using fMRI. Consistent with the intrinsic severity hypothesis of drug resistant epilepsy, where the inherent severity of epilepsy determines medication response,^24^ we hypothesize that network aberrations in epilepsy lie on a spectrum according to seizure control, with alterations in WC-IGE lying between those of DR-IGE and controls. We also tested the hypothesis that specific nodes which play a prominent role in network integration (so-called ‘hub nodes’), differ between people with IGE and controls.^18^^,^^25^ The potential importance of hub nodes in seizure susceptibility in focal epilepsy is well described,^26^^,^^27^ but hub nodes have been seldom explored in IGE.

## Materials and methods

### Recruitment

Thirty-five participants with IGE were recruited from the Walton Centre NHS Foundation Trust and from Salford Royal NHS Foundation Trust. All participants with IGE had been diagnosed by an experienced epileptologist according to current International League Against Epilepsy (ILAE) criteria ^3^ based on patient history, seizure semiology and EEG. Two participants were subsequently excluded. This was due to the re-classification of epilepsy type in one case and in the other, there was an MRI finding of focal cortical dysplasia (this was an incidental finding, the syndromic classification of IGE remains following review of diagnosis). Twenty-three participants had DR-IGE (persistent seizures despite AED treatment) and 10 were seizure-free for at least one year and therefore were classified as having WC-IGE. Thirty-four healthy controls were recruited locally from the University of Liverpool and the general public. None of the participants was familiar with the scanning environment.

Informed, written consent was obtained for all participants according to the Declaration of Helsinki and this study was approved by the local ethics committee (UK Research Ethical Committee reference 14/NW/0332).

### Data collection and pre-processing

3D T1-weighted and resting state fMRI (RS-fMRI) images were obtained for each participant using a 3T GE Discovery MR 750 MR system. Scanning was performed supine in the head-first orientation. Participants were instructed to stay awake and to look at a white fixation cross on a black background. T1-weighted data were acquired using the following parameters: Pulse sequence = BRAVO; echo time (TE) = 3.22 ms; repetition time (TR) = 8.2 ms; field of view (FOV) = 24, TI = 450 ms; slice thickness = 1 mm; voxel size = 1 mm × 1 mm × 1 mm; 140 slices; flip angle = 12. RS-fMRI was obtained with a 6-min^3^ T2-weighted sequence with the following parameters: Pulse sequence = gradient echo; TE = 25 ms; TR = 2000 ms; FOV = 24; slice thickness = 2.4 mm; voxel size = 3 mm × 3 mm × 3 mm; 180 volumes; 38 slices; flip angle = 75.

Spatial pre-processing was implemented in SPM12 using the standard SPM pipeline. Slice timing correction of the fMRI time series was performed using the first slice as the reference. Head motion and EPI distortion were corrected to the first functional volume. The estimated movement parameters (3 translation; 3 rotation) were saved and later included as covariates for each subject in the first level analysis to produce the connectivity matrix. Data were normalized into MNI (Montreal Neurological Institute) space using the ICBM 152 template of European brains^28^; the mean functional image was registered to the template image via a direct affine and interpolated into 2 × 2 × 2 mm voxel space using 4th-degree B-Spline method. The resulting warp parameter was then applied to all volumes. Gaussian kernel smoothing with an 8 mm full width half-maximum Gaussian kernel was employed at each data point and neighbourhood voxel. Tissue segmentation was performed using the SPM add-on CAT12 toolbox (http://www.neuro.uni-jena.de/cat/. Last accessed 18/05/21). This spatially normalizes the T1-weighted image into the MNI space then segments it into skull-stripped brain. Following this, adaptive maximum a posteriori segmentation^29^ was performed to quantify estimates of grey matter, white matter and cerebrospinal fluid present at each element. An exclusion threshold for motion >3 mm translation and >1° rotation was set.^30^

Spatially pre-processed data were next temporally pre-processed using the Functional Connectivity Toolbox (CONN).^31^ Component-based noise correction using the CompCor method^32^ was performed to reduce voxel specific noise, including noise arising from cardiac pulsations and respiratory modulations. Potential confounds from white matter and cerebrospinal fluid (based on principal component analysis of the multivariate BOLD signal within masks produced from T1-weighted tissue segmentation for each subject) were added as covariates in CONN. Head motion effects that were detected in spatial pre-processing (6 estimated movement parameters per volume) were used as covariates to further reduce noise. These steps are reported to increase the sensitivity of results of both correlated and anticorrelated networks.^31^ Furthermore, white matter and cerebrospinal fluid compartments were entered as covariates to reduce partial volume effects. Bandpass filtering was also implemented to further remove physiological noise and to limit BOLD to between 0.01 and 0.08 Hz. Networks within this frequency range are widely reported to represent the resting state of the brain.^31^^,^^33–35^

### Network construction

**Figure 1:**
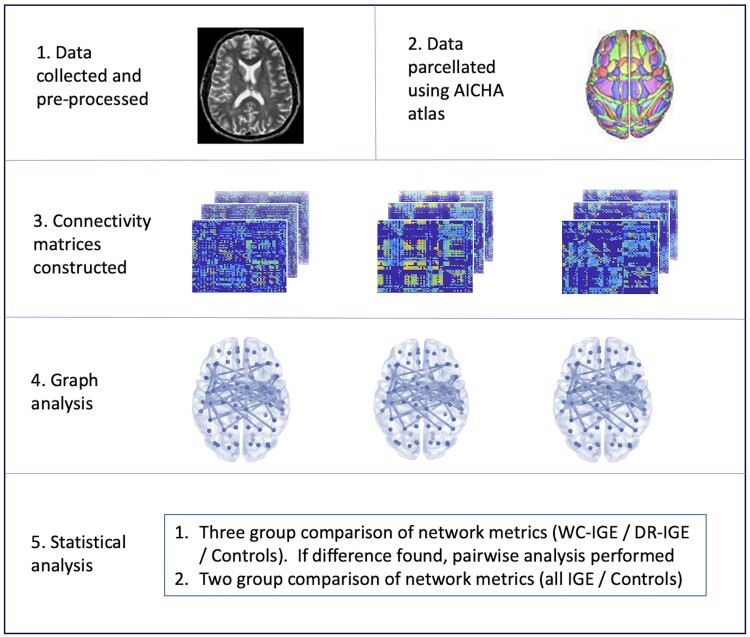
**Schematic overview of study methodology.** After data were collected and pre-processed, parcellation into network nodes was performed. Connectivity matrices were constructed for each participant. Graphs were created for each participant in each group, followed by group-level statistical analysis.

Weighted functional connectivity matrices were constructed using the CONN functional connectivity toolbox (Fig. 1). Data were parcellated using AICHA (Atlas of Intrinsic Connectivity of Homotopic Areas).^36^ This functional resting state connectivity atlas segregates data into 384 regions comprising 244 gyral regions, 100 sulcal regions and 40 grey matter nuclei. Network edges were defined using a weighted least squares linear model, where Pearson’s correlation of average BOLD signal was determined between each pair of regions, with the strength of correlation forming the weight. This was Fisher transformed to provide normally distributed scores, producing Z, representing the weighted matrix of Fisher transformed correlation coefficients.

Weighted, undirected, graphs were subsequently constructed using a custom script implemented in Matlab.^37^ Thresholding was performed in order to improve sensitivity to physiologically relevant connections versus noise,^13^ with connections with weights between −0.25 and + 0.25 excluded. There is no universally agreed threshold value, with variations from *r* = 0.1 to *r* = 0.8 seen in the literature.^38^ A threshold of *r* = 0.25 was selected as it is a commonly used threshold.^39^ A *post hoc* analysis explored the effects of altering the threshold by performing the analysis with thresholds of zero, *r* = 0.125, and *r* = 0.375. There is no optimal solution to handle negative values in graph theoretical analysis^40^; typically either positively correlated values or absolute values are used.^41^ The rationale for discarding negatively correlated edges comes from studies demonstrating that anticorrelated networks reflect artefact generated in pre-processing.^42^^,^^43^ However, there is also evidence to suggest that anticorrelated networks have an important role in brain functioning^44^^,^^45^ and as such, relevant connectivity information may be overlooked if negative correlations are ignored.^40^ In view of this debate, and the fact that graph theoretic measures cannot account for signed weights, two separate analyses were performed for global metrics; one based on networks created from only positive correlations, and the other using absolute correlations.

### Graph analysis

Global measures of average node strength, node strength distribution variance, average clustering coefficient, characteristic path length, small-world index and average betweenness centrality were calculated. These metrics were chosen to provide a broad overview of network topology. Because clustering coefficient and characteristic path length are sensitive to degree, normalized metrics were calculated for each by dividing average clustering coefficient and characteristic path length by the mean of the clustering coefficient and characteristic path length distributions of 500 surrogate random networks respectively.^46^^,^^47^

As a post hoc analysis, strength and betweenness centrality were also calculated for each node individually using an edge threshold of 0.25. Subsequently, ‘hub nodes’ were identified for each participant. Nodes were considered as hubs if both strength and betweenness centrality were greater than one standard deviation above the corresponding mean network value.^48–50^ The nodal metric analysis was carried out using absolute values only, and compared IGE with controls, in view of the results of the global network analysis.

### Statistical analysis

Demographic and outcome metric results were firstly assessed for normality (by reviewing kurtosis, skewness, histograms and Q–Q plots). Next, potential differences in demographics and outcome metrics between the three groups were evaluated using Kruskal–Wallis tests or one-way analysis of covariance, as appropriate. Age and epilepsy duration were included as co-variates. Where differences were found, pairwise comparisons were evaluated using a Mann–Whitney *U*- or Tukey test. This was Bonferroni corrected for multiple comparisons using a factor of six. In addition, both groups with epilepsy were combined into one cohort and global outcome metrics were compared with controls using an independent *t*-test, controlled for participant age.

Potential differences in connectivity between individual nodes in IGE compared with controls were evaluated by comparing the strength and betweenness centrality of each node, using a Mann–Whitney *U*-test. Correction for multiple comparisons was implemented using the false discovery rate (FDR)^51^ with a *q*-value of 0.1. Following the identification of hub nodes, the total number of times a node was considered a hub in each group was calculated and displayed visually. The number of hub nodes in each group was compared using a Kruskal–Wallis test.

An analysis to compare motion parameters between the participants with IGE and controls was performed. Normality of the data was tested first using a Lilliefors test and when significant (*P* < 0.05) a Mann–Whitney *U*-test was performed.

### Data availability statement

The data that support the findings of this study are available on request from the corresponding author. The original data are not publicly available due to ethical restrictions.

## Results

### Participant demographics

Median age significantly differed between groups (DR-IGE = 31 years; WC-IGE = 22.5 years; Controls = 32 years (Table 2). Kruskal–Wallis H = 8.02, *P* = 0.018). Pairwise comparisons found a difference in age between WC-IGE and controls (*P* = 0.014), with no significant differences between WC-IGE and DR-IGE (*P* = 0.094), DR-IGE and controls (*P* = 1.00), or between both IGE groups (combined) and controls (*P* = 0.066). Females comprised 59.7% of participants, with no significant difference across groups (Pearson Chi-square = 0.84, *P* = 0.656). Median duration of epilepsy was 14.5 years in DR-IGE and 6.5 years in WC-IGE. This difference was not statistically significant (Kruskal–Wallis H = 2.715, *P* = 0.099). The mean number of AEDs taken in the group with WC-IGE was 1.14 (range 1–2) and in the DR-IGE group was 1.9 (range 1–4). This difference was not statistically significant (Mann–Whitney *U* = 1.937, *P* = 0.524).

**Table 2:** Clinical details of participants with IGE

ID	Group	Age (years)	Gender	Onset age (years)	Seizure types	Antiepileptic medication	EEG findings
4	WC-IGE	25	M	19	MJ	Levetiracetam 1500 mg, valproate 1600 mg	Typical
18	WC-IGE	24	F	16	Abs, GTC	Valproate 1000 mg, lamotrigine 200 mg, levetiracetam 4000 mg	Typical
23	WC-IGE	23	M	16	Abs, GTC	Valproate 2100 mg, levetiracetam 500 mg	Typical
24	WC-IGE	19	F	13	GTC	Levetiracetam 3000 mg	NA
26	WC-IGE	18	F	15	Abs, eyelid myoclonus	Levetiracetam 2000 mg	Typical
27	WC-IGE	22	M	2	Abs, MJ	Valproate 1400 mg	NA
29	WC-IGE	56	F	3	Abs	Valproate 1500 mg	NA
31	WC-IGE	33	M	7	Abs	Valproate 1800 mg	Typical
32	WC-IGE	19	F	14	Abs, MJ	Levetiracetam 1000 mg	NA
34	WC-IGE	20	M	16	Abs, MJ, GTC	valproate 1700 mg, ethosuxamide 500 mg	Typical
1	DR-IGE	23	F	14	Abs, MJ	Levetiracetam 3000 mg, topiramate 300 mg, clobazam 10 mg	Typical
2	DR-IGE	19	M	16	IGE	Valproate 1000 mg	Typical
3	DR-IGE	19	F	8	GTC, Abs	Lamotrigine 200 mg	Normal
5	DR-IGE	60	F	13	GTC, Abs	Valproate 2500 mg	Typical
6	DR-IGE	24	M	15	GTC, MJ, abs	Levetiracetam 3000 mg, valproate 2500 mg, carbamazepine 1000 mg	Typical
7	DR-IGE	21	F	15	GTC, MJ, abs	Levetiracetam 4000 mg, valproate 2000 mg	Typical
8	DR-IGE	32	F	23	GTC, MJ	Levetiracetam 3500 mg, clobazam 15 mg	Normal
9	DR-IGE	38	M	18	GTC, MJ	Valproate 600 mg, lamotrigine 50 mg	Typical
10	DR-IGE	67	M	29	GTC, Abs	Valproate 2000 mg, lamotrigine 200 mg, clobazam 10 mg, phenobarbital 150 mg	NA
11	DR-IGE	46	F	7	Abs	Valproate 1200 mg, lamotrigine 200 mg, levetiracetam 2500 mg	Normal
13	DR-IGE	20	M	8	GTC, Abs	Valproate 2000 mg	Typical
14	DR-IGE	24	F	13	GTC, MJ	Topiramate 100 mg	NA
15	DR-IGE	35	M	6	GTC	Levetiracetam 2000 mg, valproate 2000 mg	Typical
16	DR-IGE	18	M	14	GTC, Abs	Valproate 1500 mg, zonisamide 350 mg	Typical
17	DR-IGE	39	M	17	GTC	Lamotrigine 75 mg	Typical
19	DR-IGE	21	M	16	GTC, abs, MJ	Valproate 2400 mg	NA
20	DR-IGE	36	F	17	GTC	Levetiracetam 1250 mg, lamotrigine 75 mg	Typical
21	DR-IGE	31	F	15	GTC	Levetiracetam 2000 mg, lamotrigine 400 mg	Normal
22	DR-IGE	31	F	16	GTC, MJ, Abs	Valproate 1500 mg, levetiracetam 3500 mg	Typical
25	DR-IGE	58	F	15	GTC, Abs	Valproate 1000 mg, zonisamide 400 mg, clonazepam 1.5 mg	Typical
28	DR-IGE	24	M	13	MJ, abs	Valproate 1700 mg	Typical
30	DR-IGE	57	F	7	GTC, abs	Valproate 1200 mg, carbamazepine 600 mg	Typical
33	DR-IGE	57	F	7	GTC, abs	Valproate 2000 mg, lamotrigine 75 mg	Typical

abs, absence; F, female; GTC, generalized tonic-clonic; M, male; MJ, myoclonic jerk; NA, not available; normal, normal interictal EEG; typical, interictal EEG findings consistent with IGE.

There was no significant difference in average root mean square motion values between groups (Mann–Whitney *U* = 1197, mean controls = 0.27, mean IGE = 0.33, *P* = 0.48).

### Global outcome metrics

**Figure 2:**
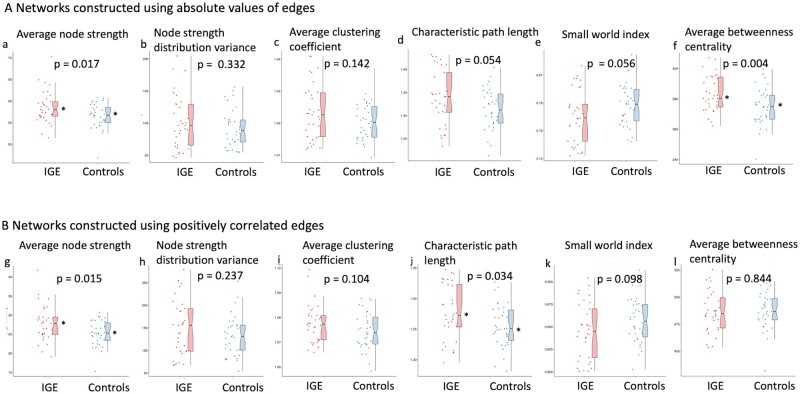
**Global outcome metrics.** (**A**) Networks constructed using absolute values of edges. (**B**) Networks constructed using positively correlated edges. Data are plotted for IGE (WC-IGE and DR-IGE combined) and controls. *Statistically significant difference between groups (*P* < 0.05).

In the graphs constructed using absolute values at a threshold of 0.25 (Supplementary Tables 1–3), there was a difference between the three groups in average betweenness centrality (one-way ANOVA F = 4.657, *P* = 0.013). Pairwise comparisons identified a significantly higher average betweenness centrality in WC-IGE compared with controls (*P* = 0.048) and a possible trend towards a significantly higher average betweenness centrality in DR-IGE compared with controls (*P* = 0.057), with no difference between WC-IGE and DR-IGE (*P* = 1). There were no other differences in global metrics at the three-group level. When both IGE groups (WC-IGE and DR-IGE combined) were compared with controls, a higher average node strength (Fig. 2a) and average betweenness centrality (Fig. 2f) were found in the group with IGE (respectively; *t* = 5.956, *P* = 0.017; *t* = 8.963, *P* = 0.004). A trend toward a significantly higher characteristic path length (Fig. 2d) and lower small-world index (Fig. 2e) was seen in IGE (respectively; *t* = 3.864, *P* = 0.054; *t* = 3.787, *P* = 0.056). There were no differences in node strength distribution variance (Fig. 2b) or clustering coefficient (Fig. 2c) between the two groups.

In the graphs constructed using positively correlated edges only, using a threshold of 0.25, there were no significant results at the three-group level. A higher average node strength (Fig. 2g) and greater characteristic path length (Fig. 2j) was identified in IGE (WC-IGE and DR-IGE combined) compared with controls (respectively; *t* = 6.200, *P* = 0.015; *t* = 4.717 *P* = 0.034). The remaining outcome metrics did not significantly differ between the two groups (Fig. 2h, j–l).

There was no correlation between age or epilepsy duration with any outcome metric (Pearson’s correlation *P* > 0.05 in all comparisons).

When edge thresholds of zero, 0.125, and 0.375 were tested, the finding of a statistically higher average node strength in the IGE group (WC-IGE and DR-IGE combined) compared with controls was robust across all thresholds and for networks constructed with both absolute edge values (respectively; *P* = 0.08, *P* = 0.02, *P* = 0.015) and positive edge values (respectively; *P* = 0.019, *P* = 0.018, *P* = 0.011). As with the threshold level of 0.25, higher characteristic path length and greater average betweenness centrality were also found in networks constructed with an edge threshold of 0.375 (respectively; *P* = 0.039, *P* = 0.041). Additionally, small world index was lower in IGE than controls using a threshold of 0.375 (using absolute edge values, *P* = 0.034) and with positive edges without a threshold (*P* = 0.035). Clustering coefficient was higher in IGE compared with controls with a threshold of 0.375 (using positive edge values, *P* = 0.011). No other significant differences were found in other group comparisons. Full details of results across various thresholds can be found in Supplementary Table 3.

### Nodal outcome metrics

**Figure 3:**
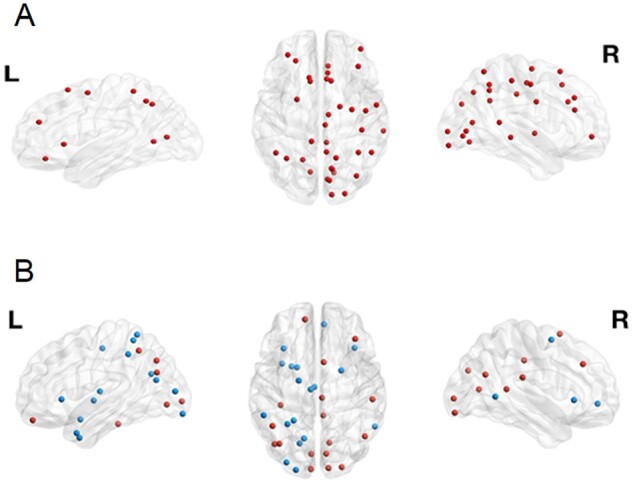
**Nodal differences between IGE and controls.** This illustrates the location of nodes that have significantly different uncorrected outcome metrics. (A) Node strength and (B) betweenness centrality. Red dots indicate a higher value in IGE, blue dots represent a lower value in IGE. L = left side of brain; R = right side of brain. This figure was created using BrainNet Viewer.^53^

Neither betweenness centrality nor node strength survived correction for multiple comparisons (Supplementary Tables 4 and 5). This was an exploratory study with 384 comparisons and therefore, results of uncorrected significant results, which may suggest a trend towards significance, are presented together with effect sizes.^52^ Uncorrected significant differences in betweenness centrality and node strength at the level of individual nodes between the IGE group and controls were found in 37 and 35 nodes respectively. Node strength was higher in IGE in each of the 35 nodes (Fig. 3A), whereas there was a greater betweenness centrality at some of the 37 nodes in IGE and a lower value in others (Fig. 3B).

The median number of hub nodes in each group was 38 and there was no significant difference in the total number of hub nodes between each group, in either the three group or two group comparison (respectively; Kruskal–Wallis *U* = 0.593, *P* = 0.743; Mann–Whitney *U* = 617, *P* = 0.671). On inspection of plots of the frequency of hub nodes at each location, there were no clear group differences between the location of hub nodes (Supplementary Table 6, Figs. 1–5).

## Discussion

This study investigated global RS-fMRI network features in people with DR-IGE, WC-IGE and healthy controls. The results suggest that compared with controls, network topology in IGE is less integrated and more regular (as evidenced by a higher path length) and has generally greater connectivity across the network nodes (demonstrated by a higher average node strength and average betweenness centrality), without a clear difference in the location of hub nodes. Network topology did not vary according to seizure control.

A higher characteristic path length results in a more regular network topology.^54^ It has been suggested that a regular configuration may render a network more vulnerable to synchronization.^15^ The finding of a higher characteristic path length in IGE, is consistent with the findings from a meta-analysis of functional connectivity studies in focal epilepsy using fMRI and EEG,^15^ and in structural studies in IGE.^19^^,^^55^^,^^56^ However, in the two fMRI-derived functional connectivity studies^18^^,^^57^ identified in our systematic review,^16^ there was no difference in characteristic path length between people with IGE and controls. However, in both previous studies, networks were constructed using absolute correlations whereas the finding of altered characteristic path length in the present study was in positively correlated networks. In these same studies, also in contrast to the present study, one reported a lower clustering coefficient and small-world index in IGE,^18^ and the other reported a higher small-world index in IGE.^57^ Average betweenness centrality and average node strength were not considered in these two studies. An important difference in our study compared with both of these studies is the method by which data were parcellated into nodes; in our study, a functional connectivity atlas was used, whereas the others used an anatomical atlas. It is known that the technique of data parcellation may affect connectivity measures^58^ and as such this is an important methodological decision. In functionally derived data parcellation schemes, nodes comprise components with similar temporal activation patterns. As such, it is suggested that such atlases are particularly suitable for functional connectivity analysis as the nodes reflect functionally coherent areas.^59–61^

The average node strength of a network reflects the strength of connections of each node across the network. Therefore, networks that have a higher average node strength perhaps reflect networks with generally greater connectivity. Similarly, networks with higher average betweenness centrality (a measure of the extent of ‘information flow’ within a network), may also reflect a greater resting state hyperexcitability of the epileptic brain.^62^ There are limited studies within the IGE literature that have considered these metrics. Increased average betweenness centrality, average node strength, or mean degree have been reported in at least two EEG/MEG studies.^63^^,^^64^ However, other studies have reported no difference between groups,^65^ or a decreased value^55^ It should be noted that the comparison between fMRI and EEG/MEG is challenging owing to their different sensitivities to temporal and spatial resolution, which may account for diverging findings.^16^

*Post hoc* analysis of global metrics using a range of edge thresholds showed that the results were robust for average node strength and for betweenness centrality at higher threshold values. The additional significant findings of increased clustering coefficient in IGE and lower small world index were also in support of a more regular network topology in IGE.

When the results using different thresholds of edges are compared, it is evident that the choice of threshold can alter the results. There appears to be a relationship between higher threshold values and the detection of a greater number of statistically significant results. Thresholds are applied to edges with the aim of decreasing sensitivity to spurious connections, but the optimal method to achieve this aim is not known. It is important to note that networks with lower summed synchronization values will become relatively less dense than networks with higher overall synchronization values after thresholding.^41^ This is a potential limitation since it is known that network density may affect some network metrics (particularly clustering coefficient and characteristic path length).^66^ This issue could potentially be overcome by constructing matrices that have the same number of connections in each network. However, this approach may result in networks with overall low connectivity producing fewer significant connections whilst potentially important connections in higher density networks may be disregarded.^66^

The additional findings depending on whether negatively correlated edges were discarded highlight that network topology is sensitive to the sign of the edge. As discussed above, the significance of anticorrelated networks and the extent to which they are influenced by pre-processing techniques are not fully elucidated. We suggest that by using absolute values, correlation values may be regarded as a reflection of the strength of neural connectivity, irrespective of the nature of the relationship. The similarity of results of both analyses suggests that the results are not confounded by taking into account negative correlations and in fact, their inclusion may improve sensitivity to the detection of network differences. How negative correlations may be mathematically accounted for in graph theoretic analysis is an important consideration for future graph theoretical studies.

Previous fMRI connectivity studies have reported widespread locations of specific nodes that display altered connectivity in IGE, with a similar location of hub nodes in IGE and controls.^18^^,^^57^ Both studies corrected for multiple comparisons using the FDR, but the threshold used is unclear. In Liao *et al*.’s study, significance levels did not survive this correction. Notwithstanding the fact that the individual nodal comparisons did not survive correction for multiple comparisons in our study, there is no suggestion from our study or from these previous studies, that there are specific regions of altered resting state connectivity in IGE. Whilst corticothalamic regions have been implicated in seizure genesis, it is possible that in generalized seizure disorders, the precise area of network aberration from where a seizure is initiated may vary between, or within, individuals.^67^

It is possible that differences in network features in the group with IGE compared with controls represent medication effects. Previous studies have described alterations in global efficiency (inverse of characteristic path length) with topiramate, but not with valproate, lamotrigine or levetiracetam.^68^ Another study reported altered betweenness centrality (but not other network metrics) with carbamazepine, but not with other commonly used AEDs.^69^ Therefore, overall, there is no strong evidence that medication effects directly explain the results. The inclusion of a group with epilepsy not taking an AED would help clarify this, but this would be practically difficult since AEDs are typically started at diagnosis.

This study did not find any differences in network topology dependent upon seizure control. One limitation of this interpretation is the small sample size, particularly in the WC-IGE group, which may have been underpowered to detect a possible difference. The low number of participants recruited with WC-IGE reflects the fact that they are less likely to remain under long-term follow up. In addition to potential underpowering, a small sample size may also result in a type 1 error.^70^ Normal values of network metrics in healthy controls have not been established, thus performing a power calculation is limited by the lack of a meaningful parameter to include in such a calculation. The reliability of our findings would be strengthened by reproducing the results in an independent dataset.

A larger study may have also permitted the comparison of IGE subtypes. Although there is strong evidence to support that IGE syndromes share pathophysiological and genetic relationships,^71^ it is possible that connectivity features vary between subtypes.^72^ Further studies with larger sample numbers are needed to explore whether IGE subtypes have different network features. Larger collaborations between institutions could help increase sample numbers ^73^^,^^74^ and clarify these areas of uncertainty. These study groups also differed in terms of age and epilepsy duration (although the latter was not statistically significant). The inclusion of these factors as covariates in the statistical analysis guards against confounding, however, it remains possible that the results were influenced by these differences.^75^^,^^76^ Though the number of AEDs taken by each of the IGE groups did not differ statistically, it is likely that the drug burden is higher in the DR-IGE group due to generally higher doses and a tendency towards polypharmacy. As such, this may also confound potential differences between the groups.

A further potential limitation relates to the difficulties in classifying response to AEDs; Patients may not be concordant with their antiepileptic medication and therefore may be inaccurately categorized as drug resistant. Alternatively, they may have unrecognized co-existent non-epileptic attacks, which could result in a seemingly higher seizure frequency. Conversely, there is evidence that some patients under-report seizures,^77^ which could potentially result in an incorrect classification of well-controlled epilepsy. In addition, it is known that a proportion of patients follow a fluctuating course, shifting in and out of seizure control.^5^ A larger study may enable the inclusion of this subgroup as a third category. Although people who fulfil the ILAE definition of seizure freedom have a lower risk of seizure recurrence within the next 12 months ^78^ and improved quality of life,^79^ it should be noted that there are potential issues with dichotomization of variables such as seizure frequency.^80^ In future studies, it may be of value to examine network metrics in relation to time since the last seizure.

A further limitation of this study is that interictal epileptiform discharges (IEDs) in the group with IGE may have confounded the results. IEDs are associated with co-localized BOLD activation, in addition to BOLD activation in distant areas.^81^ A combined EEG-fMRI study could overcome this limitation but would considerably add time, complexity and cost.

## Conclusions

In summary, this study demonstrates that the network topology in IGE is more regular and has higher global connectivity, with no evidence of systematic alteration in the location of nodes with high connectivity. This was found to be the case irrespective of seizure control. We suggest that examining drug resistance from a network perspective warrants further exploration in a larger, longitudinal, multimodal study.

## Supplementary material

Supplementary material is available at *Brain Communications* online.

## Funding

S.S.K. acknowledges support from the UK Medical Research Council (MR/S00355X/1 and MR/K023152/1) and Epilepsy Research UK (1085). P.L. is supported by the National Institute for Health Research (NIHR) Biomedical Research Centre at South London; Maudsley NHS Foundation Trust; King’s College London; Innovative Medicines Initiative 2 Joint Undertaking (Grant/Award number: 115902); European Union’s Horizon 2020 Research and Innovation Programme; EFPIA.

## Competing interests

The authors report no competing interests.

## Supplementary Material

fcab196_Supplementary_Data

## Abbreviations

AED =: antiepileptic drug
BOLD =: blood oxygen level dependent
DR-IGE =: drug resistant idiopathic generalized epilepsy
FDR =: false discovery rate
fMRI =: functional MRI
IED =: interictal epileptiform discharge
IGE =: idiopathic generalized epilepsy
MEG =: magnetoencephalography
RS-fMRI =: resting state functional MRI
WC-IGE =: well-controlled idiopathic generalized epilepsy
