# Supplementary material for: Functional network topology in drug resistant and well-controlled idiopathic generalized epilepsy: a resting state functional MRI study
Source: Brain Commun. 2021 Aug 26;3(3):fcab196. doi: 10.1093/braincomms/fcab196 (PMC8417840; doi:10.1093/braincomms/fcab196)
Supplement: fcab196_Supplementary_Data [file fcab196_supplementary_data.zip › Original Submission.pdf]

OXFORD UNIVERSITY PRESS | Brain Communications

Functional network topology in drug resistant and well-controlled Idiopathic Generalised Epilepsy: A resting-state fMRI study

Journal:	*Brain Communications*
Manuscript ID	BRAINCOM-2020-365
Manuscript Type:	Original Article
Date Submitted by the Author:	20-Nov-2020

Complete List of Authors:	Pegg, Emily; Salford Royal NHS Foundation Trust Greater Manchester Neuroscience Centre, Neurology; The University of Manchester,<br>McKavanagh, Andrea; University of Liverpool<br>Bracewell, Martyn; The Walton Centre NHS Foundation Trust<br>Chen, Yachin; University of Liverpool<br>Das, Kumar; The Walton Centre NHS Foundation Trust<br>Denby, Christine; The Walton Centre NHS Foundation Trust<br>Laiou, Petroula ; King's College London Institute of Psychiatry Psychology and Neuroscience<br>Marson, Anthony; University of Liverpool,<br>Mohanraj, Rajiv; Salford Royal Hospital Greater Manchester Neuroscience Centre<br>Taylor, Jason ; The University of Manchester<br>Keller, Simon; University of Liverpool

Keywords:	graph theory, drug resistant epilepsy, idiopathic generalised epilepsy, fMRI, connectivity, network analysis

SCHOLARONE™
Manuscripts

Functional network topology in drug resistant and well-controlled Idiopathic Generalised Epilepsy: A resting-state fMRI study

Emily J Pegg[1,2], Andrea McKavanagh[3], R Martyn Bracewell[4], Yachin Chen[3], Kumar Das[4], Christine Denby[4], Petroula Laiou[5], Tony Marson[3], Rajiv Mohanraj[1,2], Jason R Taylor[2,6], Simon S Keller[3].

[1] Department of Neurology, Manchester Centre for Clinical Neurosciences, Salford Royal NHS Foundation Trust.

[2] Division of Neuroscience and Experimental Psychology, School of Biological Sciences, Faculty of Biology, Medicine and Health, University of Manchester.

[3] Department of Pharmacology and Therapeutics, Institute of Systems, Molecular and Integrative Biology, University of Liverpool.

[4] The Walton Centre NHS Foundation Trust, Liverpool.

[5] Department of Biostatistics and Health Informatics, Institute of Psychiatry, Psychology and Neuroscience, King's College London, London.

[6] Manchester Academic Health Sciences Centre, University of Manchester.

For Review Only

Corresponding author:

Dr Emily Pegg
Department of Neurology
Manchester Centre for Clinical Neurosciences
Salford Royal Hospital NHS Foundation Trust
Stott Lane
Salford M6 8HD
United Kingdom
Email: emily-pegg@doctors.org.uk

Abstract

Despite an increasing number of drug treatment options for people with Idiopathic Generalised Epilepsy, drug resistance remains a significant issue and the mechanisms underlying it remain poorly understood. Previous studies have largely focused on potential cellular or genetic explanations for drug resistance. However, epilepsy is understood to be a network disorder and there is a growing body of literature suggesting altered topology of large-scale resting networks in people with epilepsy compared to controls. We hypothesise that network alterations may also play a role in seizure control. The study aim was to compare resting state functional network structure between well-controlled IGE (WC-IGE), drug resistant IGE (DR-IGE), and healthy controls. 35 participants with IGE (23 with WC-IGE and 12 with DR-IGE) and 34 controls were recruited. Resting-state functional MRI networks were reconstructed using the Functional Connectivity Toolbox (CONN). Global graph theoretic network measures of average node strength, node strength distribution variance, characteristic path length, average clustering coefficient, small world index, and average betweenness centrality were computed. Graphs were constructed separately for positively weighted connections and for absolute values. Individual nodal values of strength and betweenness centrality were also measured and 'hub nodes' were compared between groups. Outcome measures were assessed across the three groups and between the whole group with IGE and controls. The IGE group as a whole had a higher average node strength, characteristic path length, and average betweenness centrality, irrespective of seizure control. Outcome metrics were sensitive to whether negatively correlated connections were included in the network construction. There were no clear differences in the location of 'hub nodes' between groups. The results suggest that the IGE interictal network topology is more regular and has a higher global connectivity compared to controls, with no alteration in the location of hub nodes. These alterations may produce a resting state network that is more vulnerable to transitioning to the seizure state. It is also demonstrated that network topological features are influenced by the sign of connectivity weights and therefore future methodological work is warranted to account for anticorrelations in graph theoretic studies.

Key words: graph theory, drug resistant epilepsy, fMRI, connectivity, network analysis

For Review Only

Introduction

Epilepsy affects around 70 million people worldwide (Ngugi *et al.*, 2010), of whom 15-20% are estimated to have Idiopathic Generalised Epilepsy (IGE) (Jallon and Latour, 2005). IGEs comprise a group of syndromes characterised by the occurrence of generalised seizures in the absence of neurodevelopmental abnormalities or structural brain lesions (Scheffer *et al.*, 2017). Approximately 18% of people with IGE do not become seizure free despite an adequate trial of at least two appropriate and tolerated antiepileptic drugs (AEDs) (Semah *et al.*, 1998; Brodie *et al.*, 2012). Subsequent changes to drug regimens have a low chance of resulting in seizure freedom (Mohanraj and Brodie, 2006) and, therefore, such patients are considered to have drug resistant epilepsy (Kwan *et al.*, 2010). In addition to a high seizure burden, people with drug resistant epilepsy have a higher rate of injury (Beghi *et al.*, 2002), sudden unexplained death in epilepsy (SUDEP) (Tomson, 2000), and social difficulties (Ridsdale *et al.*, 2017), compared to those with controlled seizures.

Traditionally, drug resistance in epilepsy has been examined from a cellular or genetic perspective. However, such approaches have failed to fully explain the underlying mechanisms of drug resistance (Tang *et al.*, 2017). Since epilepsy is now understood to be a network disorder, in which seizures emerge from the dynamic resting state of the brain (Richardson, 2012), investigating epilepsy drug resistance from a resting-state network perspective may facilitate greater understanding of this important issue.

Resting state brain networks may be examined using functional magnetic resonance imaging (fMRI), whereby blood oxygen level dependent (BOLD) signal is statistically analysed to establish the extent of connectivity between regions. Graph theory provides a robust mathematical method to subsequently delineate and analyse network topology (structure). Within this framework, each brain area is termed a 'node' and the connections between nodes are termed 'edges'. Edges may be weighted according to the strength of the signal. Information regarding the presence and strength of pairs of connections within a network is contained within a connectivity matrix and from this, a range of network metrics and features can be determined (*table 1*) (Newman, 2008; Rubinov and Sporns, 2010). Overall evidence from graph theoretical studies derived from electroencephalography (EEG), magnetoencephalography (MEG) and MRI suggests that networks of people with focal epilepsy and IGE have a more regular topology compared to controls (van Diessen *et al.*, 2014c; Pegg *et al.*, 2020). It has been proposed that this regularity may render the network more likely to synchronise than a network that has a more random structure (van Diessen *et al.*,

For Review Only

2014a). However, there are inconsistencies within the literature, with some studies consistent with a more random network structure in epilepsy and others not identifying any differences in network regularity (Zhang *et al.*, 2011; Elshahabi *et al.*, 2015; Lee and Park, 2019).

To our knowledge, analysing fMRI-derived functional connectivity from a global network perspective in IGE according to seizure control has not previously been considered. However, reduced connectivity in specific networks (cerebellar and default mode networks) in drug resistant IGE (DR-IGE) compared with well-controlled IGE (WC-IGE) has been described (Kay *et al.*, 2013; Kay *et al.*, 2014). In an EEG topology study by our group, differences were found between controls and WC-IGE in the 10-12 Hz frequency band (submitted for publication). This perhaps suggests that in people who respond to medication, drug induced alterations to the network render it less susceptible to seizures.

Considering that network topology may play a role in seizure control in IGE, and that diverging findings in the literature of IGE network topology may be influenced by a lack of evaluation according to seizure control (Pegg *et al.*, 2020), the aim of this study was to compare resting state global network topology in people with DR-IGE, WC-IGE, and controls, using fMRI. Consistent with the intrinsic severity hypothesis of drug resistant epilepsy, where the inherent severity of epilepsy determines medication response (Rogawski and Johnson, 2008), we hypothesise that network aberrations in epilepsy lie on a spectrum according to seizure control, with alterations in WC-IGE lying between those of DR-IGE and controls. We also tested the hypothesis that specific nodes which play a prominent role in network integration (so called 'hub nodes'), differ between people with IGE and controls (Frei *et al.*, 2010; Zhang *et al.*, 2011). The potential importance of hub nodes in seizure susceptibility in focal epilepsy is well described (Lopes *et al.*, 2017; Lee *et al.*, 2018), but hub nodes have been seldom explored in IGE.

For Review Only

Materials and methods

Recruitment

Thirty-five participants with IGE were recruited from the Walton Centre NHS Foundation Trust and from Salford Royal NHS Foundation Trust. All participants with IGE had been diagnosed by an experienced epileptologist according to current ILAE criteria (Scheffer *et al.*, 2017) based on patient history, seizure semiology and EEG. Thirty-five people with IGE were recruited, two of whom were subsequently excluded. This was due to re-classification of epilepsy type in one case. In the other, there was an MRI finding of focal cortical dysplasia

(this was an incidental finding, the syndromic classification of IGE remains following review of diagnosis). Twenty-three participants had DR-IGE (persistent seizures despite AED treatment) and 10 were seizure free for at least one year and therefore were classified as having WC-IGE. Thirty-four healthy controls were recruited. Informed, written consent was obtained for all participants (UK Research Ethical Committee reference 14/NW/0332).

Data collection and pre-processing

3D T1 weighted and resting state fMRI images were obtained for each participant using a 3T GE Discovery MR 750 MR system. Scanning was performed supine in the head-first orientation. Participants were instructed to close their eyes and to remain awake. T1-weighted data was acquired using the following parameters: Pulse sequence = BRAVO; echo time (TE) = 3.22 ms; repetition time (TR) = 8.2 ms; field of view (FOV) = 24, TI = 450 ms; slice thickness = 1 mm; voxel size = 1 mm × 1 mm x 1 mm; 140 slices; flip angle = 12. RS-fMRI was obtained with a 6-minute T2-weighted sequence with the following parameters: Pulse sequence = gradient echo; TE = 25 ms; TR = 2,000 ms; FOV= 24; slice thickness = 2.4 mm; voxel size = 3mm x 3mm x 3mm; 180 volumes; 38 slices; flip angle = 75.

Spatial pre-processing was implemented in SPM12 using the standard SPM pipeline. Slice timing correction of the fMRI time series was performed using the first slice as the reference. Head motion and EPI distortion were corrected to the first functional volume. The estimated movement parameters (3 translation; 3 rotation) were saved and later included as covariates for each subject in the first level analysis to produce the connectivity matrix. Data were normalised into MNI (Montreal Neurological Institute) space using the ICBM 152 template of European brains (Mazziotta *et al.*, 2001); the mean functional image was registered to the template image via a direct affine and interpolated into 2 x 2 x 2 mm voxel space using 4th degree B-Spline method. The resulting warp parameter was then applied to all volumes. Gaussian kernel smoothing with an 8mm full width half-maximum Gaussian kernel was employed at each data point and neighbourhood voxel. Tissue segmentation was performed using the SPM add-on CAT12 toolbox (http://www.neuro.uni-jena.de/cat/). This spatially normalises the T1 weighted image into the MNI space then segments it into skull-stripped brain. Following this, adaptive maximum a posteriori segmentation (AMAP) (Rajapakse *et al.*, 1997) was performed to quantify estimates of grey matter, white matter and cerebrospinal fluid present at each element.

Spatially pre-processed data were next temporally pre-processed using the Functional Connectivity Toolbox (CONN) (Whitfield-Gabrieli and Nieto-Castanon, 2012). Component-

For Review Only

based noise correction using the CompCor method (Behzadi *et al.*, 2007) was performed to reduce voxel specific noise. Head motion effects that were detected in spatial pre-processing (6 estimated movement parameters per volume) were used as co-variates to further reduce noise. These steps are reported to increase sensitivity of results of both correlated and anticorrelated networks (Whitfield-Gabrieli and Nieto-Castanon, 2012). BOLD data were then bandpass filtered between 0.01 and 0.08 Hz. Networks within this frequency range are widely reported to represent the resting state of the brain (Biswal *et al.*, 1995; Fox and Raichle, 2007; Buckner *et al.*, 2008; Whitfield-Gabrieli and Nieto-Castanon, 2012).

Network construction

Weighted functional connectivity matrices were constructed using the CONN functional connectivity toolbox. Data were parcellated using AICHA (Atlas of Intrinsic Connectivity of Homotopic Areas) (Joliot *et al.*, 2015). This functional resting-state connectivity atlas segregates data into 384 regions comprising 244 gyral regions, 100 sulcal regions and 40 grey matter nuclei. Network edges were defined using a weighted least squares linear model, where Pearson's correlation of average BOLD signal was determined between each pair of regions, with the strength of correlation forming the weight. This was Fisher transformed to provide normally distributed scores, producing Z, representing the weighted matrix of Fisher transformed correlation coefficients.

Weighted, undirected, graphs were subsequently constructed using a custom script implemented in Matlab (R2019a). Thresholding was performed in order to improve sensitivity to more physiologically relevant connections (Rubinov and Sporns, 2010) with connections with weights between -0.25 and + 0.25 excluded (Eickhoff *et al.*, 2015). There is no optimal solution to handle negative values in graph theoretical analysis (Fornito, 2016); typically either positively correlated values or absolute values are used (Fornito *et al.*, 2013). The rationale for discarding negatively correlated edges comes from studies demonstrating that anticorrelated networks reflect artefact generated in pre-processing (Murphy *et al.*, 2009; Saad *et al.*, 2012). However, there is also evidence to suggest that anticorrelated networks have an important role in brain functioning (Kelly *et al.*, 2008; De Pisapia *et al.*, 2012) and as such, relevant connectivity information may be overlooked if negative correlations are ignored (Fornito, 2016). In view of this debate, and the fact that graph theoretic measures cannot account for signed weights, two separate analyses were performed for global metrics; one based on networks created from only positive correlations, and the other using absolute correlations.

Graph analysis

For Review Only

Global measures of average node strength, node strength distribution variance, average clustering coefficient, characteristic path length, small world index, and average betweenness centrality were calculated (*table 1*). These metrics were chosen to provide a broad overview of network topology. Because clustering coefficient and characteristic path length are sensitive to degree, normalised metrics were calculated for each by dividing average clustering coefficient and characteristic path length by the mean of the clustering coefficient and characteristic path length distributions of 500 surrogate random networks respectively (Stam *et al.*, 2006; Stam *et al.*, 2008).

As a post hoc analysis, strength and betweenness centrality were also calculated for each node individually. Subsequently, 'hub nodes' were identified for each participant. Nodes were considered to be hubs if both strength and betweenness centrality were greater than one standard deviation above the corresponding mean network value (He *et al.*, 2009; Bernhardt *et al.*, 2011; Tian *et al.*, 2011). The nodal metric analysis was carried out using absolute values only, and compared IGE with controls, in view of the results of the global network analysis.

Statistical analysis

Demographic and outcome metric results were firstly assessed for normality (by reviewing kurtosis, skewness, histograms and Q-Q plots). Next, potential differences in demographics and outcome metrics between the three groups were evaluated using Kruskall- Wallis tests or one-way analysis of co-variance (ANCOVA), as appropriate. Age and epilepsy duration were included as co-variates. Where differences were found, pairwise comparisons were evaluated using a Mann-Whitney U or Tukey test. This was Bonferroni corrected for multiple comparisons. In addition, both groups with epilepsy were combined into one cohort and global outcome metrics were compared with controls using an independent t-test, controlled for participant age.

Potential differences in connectivity between individual nodes in IGE compared to controls were evaluated by comparing the strength and betweenness centrality of each node, using a Mann-Whitney U test. Correction for multiple comparisons was implemented using the False Discovery Rate (Benjamini and Hochberg, 1995) with a q-value of 0.8. Following the identification of hub nodes, the total number of times a node was considered a hub in each group was calculated and displayed visually. The number of hub nodes in each group was compared using a Kruskall-Wallis test.

Figure 1. Schematic overview of study methodology

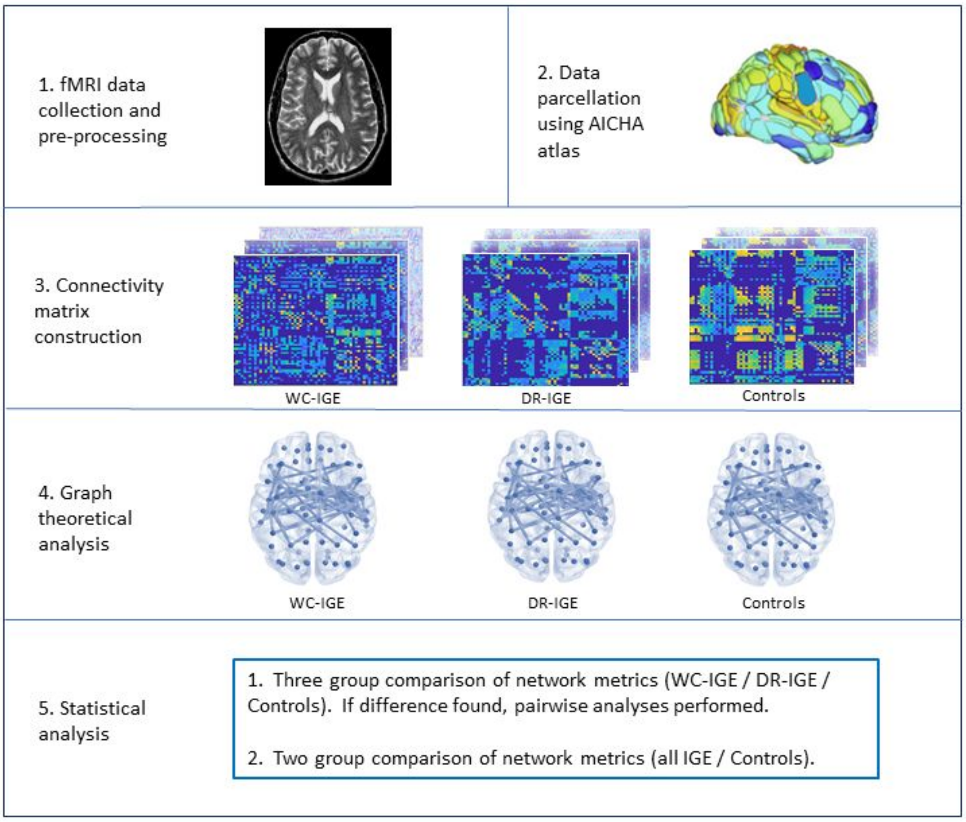

After data were collected and pre-processed, parcellation into network nodes was performed. Connectivity matrices were constructed for each participant. Graphs were created for each participant in each group, followed by group-level statistical analysis.

Only

Results

Participant demographics

(Table 2)

Median age significantly differed between groups (DR-IGE = 31 years; WC-IGE = 22.5 years; Controls = 32 years. Kruskall-Wallis H = 8.02, p = 0.018). Pairwise comparisons found a difference in age between WC-IGE and controls (p = 0.014), with no significant differences between WC-IGE and DR-IGE (p = 0.094), DR-IGE and controls (p = 1.00), or between both IGE groups (combined) and controls (p=0.066). Females comprised 59.7% of participants, with no significant difference across groups (Pearson Chi-square = 0.84, p = 0.656). Median duration of epilepsy was 14.5 years in DR-IGE and 6.5 years in WC-IGE. This difference was not statistically significant (Kruskall-Wallis H = 2.715, p = 0.099).

Global outcome metrics

(Supplementary data 1)

In the graphs constructed using absolute values, there was a difference between the three groups in average betweenness centrality (one-way ANOVA F = 4.657, p = 0.013). Pairwise comparisons identified a significantly higher average betweenness centrality in WC-IGE compared with controls (p = 0.048) and a trend towards a significantly higher average betweenness centrality in DR-IGE compared with controls (p = 0.057), with no difference between WC-IGE and DR-IGE (p = 1). There were no other differences in global metrics at the three-group level. When both IGE groups (WC-IGE and DR-IGE combined) were compared with controls, a higher average node strength *(figure 2a)* and average betweenness centrality *(figure 2f)* were found in the group with IGE (respectively; t = 5.956, p =0.017; t = 8.963, p= 0.004). A trend toward a significantly higher characteristic path length (*figure 2d*) and lower small world index (*figure 2e*) was seen in IGE (respectively; t = 3.864, p = 0.054; t= 3.787, p = 0.056, respectively). There were no differences in node strength distribution variance (*figure 2b*) or clustering coefficient (*figure 2c*) between the two groups.

In the graphs constructed using positively correlated edges only, there were no significant results at the three-group level. A higher average node strength (*figure 2g*) and greater characteristic path length (*figure 2j*) was identified in IGE (WC-IGE and DR-IGE combined) compared to controls (respectively; t = 6.200, p = 0.015; t = 4.717 p = 0.034). The remaining outcome metrics did not significantly differ between the two groups (*figures 2h,2j,2k,2l*).

There was no correlation between age or epilepsy duration with any outcome metric (Pearson's correlation p > 0.05 in all comparisons).

Nodal outcome metrics

There were significant differences in betweenness centrality and strength at the level of individual nodes between the IGE group and controls in 37 and 35 nodes respectively, but none survived correction for multiple comparisons *(supplementary data 2).*

Strength was higher in IGE in all significant nodes (*figure 3a*), whereas there was a greater betweenness centrality at some nodes in IGE and a lower value in others *(figure 3b).* The median number of hub nodes in each group was 38 and there was no significant difference in the total number of hub nodes between each group, at either the three group or two group comparison (respectively; Kruskall Wallis U = 0.593, p = 0.743; Mann-Whitney U = 617, p = 0.671). On inspection of plots of the frequency of hub nodes at each location, there were no clear group differences between the location of hub nodes *(supplementary data 2).*

For Review Only

Figure 3. Nodal differences between IGE and controls

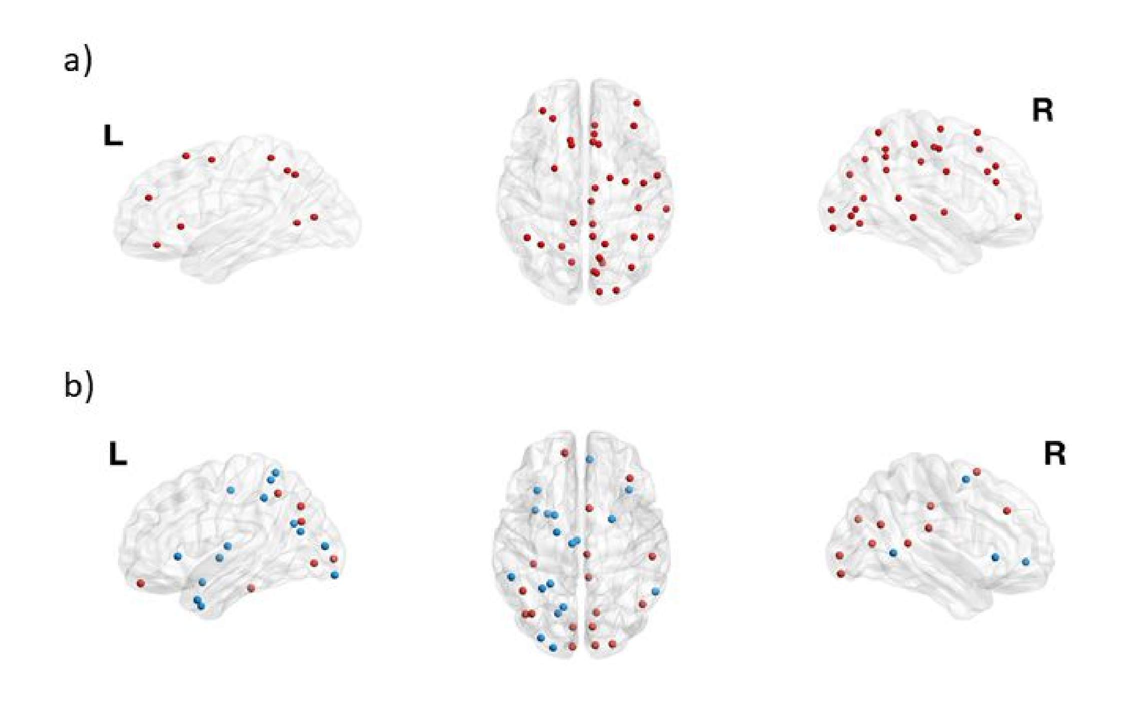

Figure 3a illustrates nodes which differ in strength between IGE and controls. Figure 3b illustrates nodes which differ in betweenness centrality between IGE and controls. L= left side of brain, R = right side of brain. Red dots indicate a higher value in IGE, blue dots represent a lower value in IGE. This figure was created using BrainNet Viewer (Xia *et al.*, 2013).

Discussion

This study investigated global resting state fMRI network features in people with drug resistant IGE, well-controlled IGE and healthy controls. The results suggest that compared to controls, network topology in IGE is less integrated (as evidenced by a higher path length) and has a generally greater connectivity across the network nodes (demonstrated by a higher average node strength and average betweenness centrality), without a clear difference in the location of hub nodes. Network topology did not vary according to seizure control.

A higher characteristic path length results in a more regular network topology (Watts and Strogatz, 1998). It has been suggested that a regular configuration may render a network more vulnerable to synchronisation (van Diessen *et al.*, 2014b). The finding of a higher

characteristic path length in IGE, is consistent with our recent similar analysis using EEG (submitted for publication) and is also consistent with the findings from a meta-analysis of functional connectivity studies in focal epilepsy using fMRI and EEG (van Diessen *et al.*, 2014c), and in structural studies in IGE (Xue *et al.*, 2014b; Qiu *et al.*, 2017; Lee and Park, 2019). However, in the two fMRI-derived functional connectivity studies (Zhang *et al.*, 2011; Liao *et al.*, 2013) identified in our systematic review (Pegg *et al.*, 2020), there was no difference in characteristic path length between people with IGE and controls. However, in both previous studies, networks were constructed using absolute correlations whereas the finding of altered characteristic path length in the present study was in positively correlated networks. In these same studies, also in contrast to the present study, one reported a lower clustering coefficient and small world index in IGE (Zhang *et al.*, 2011), and the other reported a higher small world index in IGE (Liao *et al.*, 2013). Average betweenness centrality and average node strength were not considered in these two studies. An important difference of our study compared to both of these studies is the method by which data were parcellated into nodes; in our study, a functional connectivity atlas was used, whereas the others used an anatomical atlas. It is known that the technique of data parcellation may affect connectivity measures (Arslan *et al.*, 2018) and as such this is an important methodological decision. In functionally derived data parcellation schemes, nodes comprise components with similar temporal activation patterns. As such, it is suggested that such atlases are particularly suitable for functional connectivity analysis as the nodes reflect functionally coherent areas (Shen *et al.*, 2013; Finn *et al.*, 2015; Eickhoff *et al.*, 2018).

The average node strength of a network reflects the strength of connections of each node across the network. Therefore, networks which have a higher average node strength perhaps reflect networks with generally greater connectivity. Similarly, networks with higher average betweenness centrality (a measure of the extent of 'information flow' within a network), may also reflect a greater resting state hyperexcitability of the epileptic brain (Grobelny *et al.*, 2018). There are limited studies within the IGE literature which have considered these metrics. Increased average betweenness centrality, average node strength, or mean degree (an equivalent measure in a network that is not fully connected) have been reported in at least two EEG / MEG studies (Chavez *et al.*, 2010; Chowdhury *et al.*, 2014). However, other studies have reported no difference between groups (Caeyenberghs *et al.*, 2015), or a decreased value (Xue *et al.*, 2014a). It should be noted that the comparison between fMRI and EEG / MEG is challenging owing to their differing sensitivities to temporal and spatial resolution, which may account for diverging findings (Pegg *et al.*, 2020).

For Review Only

Similar to the present study, previous fMRI connectivity studies have found widespread locations of specific nodes that display altered connectivity in IGE, and a similar location of hub nodes in IGE and controls (Zhang *et al.*, 2011; Liao *et al.*, 2013). Notwithstanding the fact that the individual nodal comparisons did not survive correction for multiple comparisons, there was no suggestion from this study, or similar studies (Zhang *et al.*, 2011; Liao *et al.*, 2013), that there are specific regions of altered resting state connectivity in IGE. Whilst corticothalamic regions have been implicated in seizure genesis, it is possible that in generalised seizure disorders, the precise area of network aberration from where a seizure is initiated may vary between, or within, individuals (Anderson and Hamandi, 2011).

The additional findings depending on whether negatively correlated edges were discarded highlights that network topology is sensitive to the sign of the edge. As discussed above, the significance of anticorrelated networks and the extent to which they are influenced by pre-processing techniques is not fully elucidated. We suggest that by using absolute values, correlation values may be regarded as a reflection of the strength of neural connectivity, irrespective of the nature of the relationship. The similarity of results of both analyses suggests that the results are not confounded by taking into account negative correlations and in fact, their inclusion may improve sensitivity to the detection of network differences. How negative correlations may be mathematically accounted for in graph theoretic analysis is an important consideration for future graph theoretical studies.

It is possible that differences in network features in the group with IGE compared to controls represent medication effects. Previous studies have described alterations in global efficiency (inverse of characteristic path length) with topiramate, but not with valproate, lamotrigine or valproate (van Veenendaal *et al.*, 2017). Another study reported altered betweenness centrality (but not other network metrics) with carbamazepine, but not with other commonly used AEDs (Haneef *et al.*, 2015b). Therefore, overall, there is no strong evidence that medication effects directly explain the results. The inclusion of a group with epilepsy not taking an AED would help clarify this, but this would be practically difficult since AEDs are typically started at diagnosis.

This study did not find any differences in network topology dependent upon seizure control. One limitation of this interpretation is the small sample size of the WC-IGE group, which may have been underpowered to detect a possible difference. The low number of participants recruited with WC-IGE is a reflection of the fact that they are less likely to remain under long-term follow up. Larger collaborations between institutions could help increase sample

For Review Only

numbers (Whelan *et al.*, 2018; Hatton *et al.*, 2020). The study groups also differed in terms of age and epilepsy duration (although the latter was not statistically significant). The inclusion of these factors as covariates in the statistical analysis guards against confounding, however it remains possible that the results were influenced by these differences (Haneef *et al.*, 2015a; Varangis *et al.*, 2019). A further potential limitation relates to the difficulties in classifying response to AEDs; Patients may not be concordant with their antiepileptic medication and therefore may be inaccurately categorised as drug resistant. Alternatively, they may have unrecognised co-existent non-epileptic attacks, which could result in a seemingly higher seizure frequency. In addition, it is known that a proportion of patients follow a fluctuating course, shifting in and out of seizure control (Brodie *et al.*, 2012). A larger study may enable the inclusion of this subgroup as a third category.

A further limitation of this study is that interictal epileptiform discharges (IEDs) in the group with IGE may have confounded the results. IEDs are associated with co-localised BOLD activation, in addition to BOLD activation in distant areas (Aghakhani *et al.*, 2015). A combined EEG-fMRI study could overcome this limitation.

Conclusions

In summary, this study demonstrates that the network structure in IGE is more regular and has higher global connectivity, with no evidence of systematic alteration in the location of nodes with high connectivity. This was found to be the case irrespective of seizure control. We suggest that examining drug resistance from a network perspective warrants further exploration in a larger, longitudinal, multimodal study.

Funding

SSK was supported by the Medical Research Council (grant numbers MR/S00355X/1 and MR/K023152/)

References

MATLAB. Natick, Massachusetts: The MathWorks Inc.; R2019a.

For Review Only

Aghakhani Y, Beers CA, Pittman DJ, Gaxiola-Valdez I, Goodyear BG, Federico P. Co-localization between the BOLD response and epileptiform discharges recorded by simultaneous intracranial EEG-fMRI at 3 T. NeuroImage: Clinical 2015; 7: 755-63.
Anderson J, Hamandi K. Understanding juvenile myoclonic epilepsy: Contributions from neuroimaging. Epilepsy Research 2011; 94(3): 127-37.
Arslan S, Ktena SI, Makropoulos A, Robinson EC, Rueckert D, Parisot S. Human brain mapping: A systematic comparison of parcellation methods for the human cerebral cortex. Neuroimage 2018; 170: 5-30.
Beghi E, Cornaggia C, the R-G. Morbidity and Accidents in Patients with Epilepsy: Results of a European Cohort Study. Epilepsia 2002; 43(9): 1076-83.
Behzadi Y, Restom K, Liau J, Liu TT. A component based noise correction method (CompCor) for BOLD and perfusion based fMRI. Neuroimage 2007; 37(1): 90-101.
Benjamini Y, Hochberg Y. Controlling the False Discovery Rate: A Practical and Powerful Approach to Multiple Testing. Journal of the Royal Statistical Society: Series B (Methodological) 1995; 57(1): 289-300.
Bernhardt BC, Chen Z, He Y, Evans AC, Bernasconi N. Graph-theoretical analysis reveals disrupted small-world organization of cortical thickness correlation networks in temporal lobe epilepsy. Cereb Cortex 2011; 21(9): 2147-57.
Biswal B, Yetkin FZ, Haughton VM, Hyde JS. Functional connectivity in the motor cortex of resting human brain using echo-planar MRI. Magn Reson Med 1995; 34(4): 537-41.
Brodie M, Barry S, Bamagous G, Norrie J, Kwan P. Patterns of treatment response in newly diagnosed epilepsy. Neurology. Hagerstown, MD; 2012. p. 1548-54.
Buckner RL, Andrews-Hanna JR, Schacter DL. The brain's default network: anatomy, function, and relevance to disease. Ann N Y Acad Sci 2008; 1124: 1-38.
Caeyenberghs K, Powell HW, Thomas RH, Brindley L, Church C, Evans J*, et al.* Hyperconnectivity in juvenile myoclonic epilepsy: a network analysis. Neuroimage Clin 2015; 7: 98-104.
Chavez M, Valencia M, Navarro V, Latora V, Martinerie J. Functional Modularity of Background Activities in Normal and Epileptic Brain Networks. Physical Review Letters 2010; 104(11): 118701.
Chowdhury FA, Woldman W, FitzGerald TH, Elwes RD, Nashef L, Terry JR*, et al.* Revealing a brain network endophenotype in families with idiopathic generalised epilepsy. PLoS One 2014; 9(10): e110136.
De Pisapia N, Turatto M, Lin P, Jovicich J, Caramazza A. Unconscious Priming Instructions Modulate Activity in Default and Executive Networks of the Human Brain. Cerebral Cortex 2012; 22(3): 639-49.
Eickhoff SB, Thirion B, Varoquaux G, Bzdok D. Connectivity-based parcellation: Critique and implications. Hum Brain Mapp 2015; 36(12): 4771-92.
Eickhoff SB, Yeo BTT, Genon S. Imaging-based parcellations of the human brain. Nature Reviews Neuroscience 2018; 19(11): 672-86.
Elshahabi A, Klamer S, Sahib AK, Lerche H, Braun C, Focke NK. Magnetoencephalography Reveals a Widespread Increase in Network Connectivity in Idiopathic/Genetic Generalized Epilepsy. PLoS One 2015; 10(9): e0138119.
Finn ES, Scheinost D, Shen X, Papademetris X, Constable RT. Methodological Issues in fMRI Functional Connectivity and Network Analysis. In: Toga AW, editor. Brain Mapping. Waltham: Academic Press; 2015. p. 697-704.
Fornito A. Fundamentals of brain network analysis. Amsterdam: Elsevier; 2016.
Fornito A, Zalesky A, Breakspear M. Graph analysis of the human connectome: promise, progress, and pitfalls. Neuroimage 2013; 80: 426-44.
Fox MD, Raichle ME. Spontaneous fluctuations in brain activity observed with functional magnetic resonance imaging. Nat Rev Neurosci 2007; 8(9): 700-11.
Frei MG, Zaveri HP, Arthurs S, Bergey GK, Jouny CC, Lehnertz K*, et al.* Controversies in epilepsy: Debates held during the Fourth International Workshop on Seizure Prediction. Epilepsy & Behavior 2010; 19(1): 4-16.

For Review Only

Grobelny BT, London D, Hill TC, North E, Dugan P, Doyle WK. Betweenness centrality of intracranial electroencephalography networks and surgical epilepsy outcome. Clinical Neurophysiology 2018; 129(9): 1804-12.
Haneef Z, Chiang S, Yeh HJ, Engel J, Jr., Stern JM. Functional connectivity homogeneity correlates with duration of temporal lobe epilepsy. Epilepsy Behav 2015a; 46: 227-33.
Haneef Z, Levin HS, Chiang S. Brain Graph Topology Changes Associated with Anti-Epileptic Drug Use. Brain connectivity 2015b; 5(5): 284-91.
Hatton SN, Huynh KH, Bonilha L, Abela E, Alhusaini S, Altmann A*, et al.* White matter abnormalities across different epilepsy syndromes in adults: an ENIGMA-Epilepsy study. Brain 2020; 143(8): 2454-73.
He Y, Wang J, Wang L, Chen ZJ, Yan C, Yang H*, et al.* Uncovering intrinsic modular organization of spontaneous brain activity in humans. PloS one 2009; 4(4): e5226-e.
Jallon P, Latour P. Epidemiology of Idiopathic Generalized Epilepsies. Epilepsia 2005; 46: 10-4.
Joliot M, Jobard G, Naveau M, Delcroix N, Petit L, Zago L*, et al.* AICHA: An atlas of intrinsic connectivity of homotopic areas. Journal of Neuroscience Methods 2015; 254: 46-59.
Kay BP, DiFrancesco MW, Privitera MD, Gotman J, Holland SK, Szaflarski JP. Reduced default mode network connectivity in treatment-resistant idiopathic generalized epilepsy. Epilepsia 2013; 54(3): 461-70.
Kay BP, Holland SK, Privitera MD, Szaflarski JP. Differences in paracingulate connectivity associated with epileptiform discharges and uncontrolled seizures in genetic generalized epilepsy. Epilepsia 2014; 55(2): 256-63.
Kelly AMC, Uddin LQ, Biswal BB, Castellanos FX, Milham MP. Competition between functional brain networks mediates behavioral variability. NeuroImage 2008; 39(1): 527-37.
Kwan P, Arzimanoglou A, Berg AT, Brodie MJ, Allen Hauser W, Mathern G*, et al.* Definition of drug resistant epilepsy: consensus proposal by the ad hoc Task Force of the ILAE Commission on Therapeutic Strategies. Epilepsia 2010; 51(6): 1069-77.
Lee HJ, Park KM. Structural and functional connectivity in newly diagnosed juvenile myoclonic epilepsy. Acta Neurol Scand 2019; 139(5): 469-75.
Lee K, Khoo HM, Lina J-M, Dubeau F, Gotman J, Grova C. Disruption, emergence and lateralization of brain network hubs in mesial temporal lobe epilepsy. NeuroImage Clinical 2018; 20: 71-84.
Liao W, Zhang Z, Mantini D, Xu Q, Wang Z, Chen G*, et al.* Relationship between large-scale functional and structural covariance networks in idiopathic generalized epilepsy. Brain Connect 2013; 3(3): 240-54.
Lopes MA, Richardson MP, Abela E, Rummel C, Schindler K, Goodfellow M*, et al.* An optimal strategy for epilepsy surgery: Disruption of the rich-club? PLOS Computational Biology 2017; 13(8): e1005637.
Mazziotta J, Toga A, Evans A, Fox P, Lancaster J, Zilles K*, et al.* A probabilistic atlas and reference system for the human brain: International Consortium for Brain Mapping (ICBM). Philosophical transactions of the Royal Society of London Series B, Biological sciences 2001; 356(1412): 1293-322.
Mohanraj R, Brodie MJ. Diagnosing refractory epilepsy: response to sequential treatment schedules. Eur J Neurol 2006; 13(3): 277-82.
Murphy K, Birn RM, Handwerker DA, Jones TB, Bandettini PA. The impact of global signal regression on resting state correlations: Are anti-correlated networks introduced? NeuroImage 2009; 44(3): 893-905.
Newman MEJ. Mathematics of Networks. The New Palgrave Encyclopedia of Economics. 2nd ed. Basingstoke; 2008.
Ngugi AK, Bottomley C, Kleinschmidt I, Sander JW, Newton CR. Estimation of the burden of active and life-time epilepsy: a meta-analytic approach. Epilepsia 2010; 51(5): 883-90.
Pegg EJ, Taylor JR, Keller SS, Mohanraj R. Interictal structural and functional connectivity in idiopathic generalized epilepsy: A systematic review of graph theoretical studies. Epilepsy Behav 2020; 106: 107013.

For Review Only

Qiu W, Yu C, Gao Y, Miao A, Tang L, Huang S*, et al.* Disrupted topological organization of structural brain networks in childhood absence epilepsy. Sci Rep 2017; 7(1): 11973.
Rajapakse JC, Giedd JN, Rapoport JL. Statistical approach to segmentation of single-channel cerebral MR images. IEEE Transactions on Medical Imaging 1997; 16(2): 176-86.
Richardson MP. Large scale brain models of epilepsy: dynamics meets connectomics. Journal of Neurology, Neurosurgery & Psychiatry 2012; 83(12): 1238.
Ridsdale L, Wojewodka G, Robinson E, Landau S, Noble A, Taylor S*, et al.* Characteristics associated with quality of life among people with drug-resistant epilepsy. J Neurol 2017; 264(6): 1174-84.
Rogawski MA, Johnson MR. Intrinsic severity as a determinant of antiepileptic drug refractoriness. Epilepsy Curr 2008; 8(5): 127-30.
Rubinov M, Sporns O. Complex network measures of brain connectivity: uses and interpretations. Neuroimage 2010; 52(3): 1059-69.
Saad ZS, Gotts SJ, Murphy K, Chen G, jo HJ, Martin A*, et al.* Trouble at Rest: How Correlation Patterns and Group Differences Become Distorted After Global Signal Regression. Brain Connectivity 2012; 2(1): 25-32.
Scheffer IE, Berkovic S, Capovilla G, Connolly MB, French J, Guilhoto L*, et al.* ILAE classification of the epilepsies: Position paper of the ILAE Commission for Classification and Terminology. Epilepsia 2017; 58(4): 512-21.
Semah F, Picot MC, Adam C, Broglin D, Arzimanoglou A, Bazin B*, et al.* Is the underlying cause of epilepsy a major prognostic factor for recurrence? Neurology 1998; 51(5): 1256-62.
Shen X, Tokoglu F, Papademetris X, Constable RT. Groupwise whole-brain parcellation from resting-state fMRI data for network node identification. NeuroImage 2013; 82: 403-15.
Stam CJ, de Haan W, Daffertshofer A, Jones BF, Manshanden I, van Cappellen van Walsum AM*, et al.* Graph theoretical analysis of magnetoencephalographic functional connectivity in Alzheimer's disease. Brain 2008; 132(1): 213-24.
Stam CJ, Jones BF, Nolte G, Breakspear M, Scheltens P. Small-World Networks and Functional Connectivity in Alzheimer's Disease. Cerebral Cortex 2006; 17(1): 92-9.
Tang F, Hartz AMS, Bauer B. Drug-Resistant Epilepsy: Multiple Hypotheses, Few Answers. Front Neurol 2017; 8.
Tian L, Wang J, Yan C, He Y. Hemisphere- and gender-related differences in small-world brain networks: A resting-state functional MRI study. NeuroImage 2011; 54(1): 191-202.
Tomson T. Mortality in epilepsy. J Neurol 2000; 247(1): 15-21.
van Diessen E, Numan T, van Dellen E, van der Kooi AW, Boersma M, Hofman D*, et al.* Opportunities and methodological challenges in EEG and MEG resting state functional brain network research. Clinical Neurophysiology 2014a; 126(8): 1468-81.
van Diessen E, Zweiphenning W, Jansen FE, Stam CJ, Braun KPJ, Otte WM. Brain Network Organization in Focal Epilepsy: A Systematic Review and Meta-Analysis. In: Doesburg S, editor. PLoS One. San Francisco, USA; 2014b.
van Diessen E, Zweiphenning WJ, Jansen FE, Stam CJ, Braun KP, Otte WM. Brain Network Organization in Focal Epilepsy: A Systematic Review and Meta-Analysis. PLoS One 2014c; 9(12): e114606.
van Veenendaal TM, DM IJ, Aldenkamp AP, Lazeron RHC, Hofman PAM, de Louw AJA*, et al.* Chronic antiepileptic drug use and functional network efficiency: A functional magnetic resonance imaging study. World J Radiol 2017; 9(6): 287-94.
Varangis E, Habeck CG, Razlighi QR, Stern Y. The Effect of Aging on Resting State Connectivity of Predefined Networks in the Brain. Frontiers in Aging Neuroscience 2019; 11: 234.
Watts DJ, Strogatz SH. Collective dynamics of 'small-world' networks. Nature 1998; 393(6684): 440-2.
Whelan CD, Altmann A, Botía JA, Jahanshad N, Hibar DP, Absil J*, et al.* Structural brain abnormalities in the common epilepsies assessed in a worldwide ENIGMA study. Brain 2018; 141(2): 391-408.
Whitfield-Gabrieli S, Nieto-Castanon A. Conn: a functional connectivity toolbox for correlated and anticorrelated brain networks. Brain Connect 2012; 2(3): 125-41.

For Review Only

Xia M, Wang J, He Y. BrainNet Viewer: A Network Visualization Tool for Human Brain Connectomics. PLOS ONE 2013; 8(7): e68910.
Xue K, Luo C, Zhang D, Yang T, Li J, Gong D*, et al.* Diffusion tensor tractography reveals disrupted structural connectivity in childhood absence epilepsy. Epilepsy Research 2014a; 108(1): 125-38.
Xue K, Luo C, Zhang D, Yang T, Li J, Gong D*, et al.* Diffusion tensor tractography reveals disrupted structural connectivity in childhood absence epilepsy. Epilepsy Res 2014b; 108(1): 125-38.
Zhang Z, Liao W, Chen H, Mantini D, Ding JR, Xu Q*, et al.* Altered functional-structural coupling of large-scale brain networks in idiopathic generalized epilepsy. Brain 2011; 134(Pt 10): 2912-28.

For Review Only

Table 1. Commonly used graph theoretical terms and measures applied to epilepsy research

Node (vertex) *(n)*	The unit which forms a graph and represents an underlying brain region
Edge<br><br>Directed edge<br><br>Undirected edge	Connection between two nodes<br><br>Information flows in one direction only<br><br>Information flows in either direction

Weighted edge	A value given to an edge according to the strength of the connection
Degree distribution variance / node strength distribution variance	The variance of the node degree /node strength distribution
Degree (*k*)<br><br>Node strength<br><br><br><br><br><br>Average node strength	Number of connections of a node<br><br>The summed strength of connections of a node<br><br>Nodes with a high number of connections or a high connectivity strength may be regarded as 'hub nodes'<br><br>The mean value of the node strength values of all network nodes
Clustering coefficient *(C)*<br><br><br>Mean clustering coefficient *(Ci)*	The probability that the neighbouring nodes of a given node are themselves connected<br><br>C is averaged to calculate the clustering coefficient of the whole graph. (A measure of network segregation)
Path length *(d)*<br><br><br>Characteristic path length *(L)*	Minimum (or shortest) number of edges connecting 2 nodes<br><br>Mean of the shortest path length between all pairs of network nodes (a measure of network integration)
Small- worldness	Ratio of average clustering coefficient of the graph to the mean clustering coefficient of a similar size random graph as a proportion of the ratio of the characteristic path length of the graph compared to the path length of a random graph<br><br>$\frac{[C / C\ random]}{[P / P\ random]}$<br><br>Small-world networks have higher than expected clustering coefficient with a characteristic path length of equal or lower value than a random graph
Betweenness centrality	A measure of to what extent a node lies on all shortest paths between each pair of network nodes.

For Review Only

	A measure of the importance of a node within the network. Nodes with high betweenness centrality may be regarding as 'hub nodes'
Average betweenness centrality	The mean value of the betweenness centrality values of all network nodes

For Review Only

Table 2. Participant demographics (IGE group)

ID	Group	Age (years)	Gender	Onset age (years)	Seizure types
4	WC-IGE	25	M	19	MJ
18	WC-IGE	24	F	16	Abs, GTCS
23	WC-IGE	23	M	16	Abs, GTCS

24	WC-IGE	19	F	13	GTCS
26	WC-IGE	18	F	15	Jeavon's
27	WC-IGE	22	M	2	Abs, MJ
29	WC-IGE	56	F	3	Abs
31	WC-IGE	33	M	7	Abs
32	WC-IGE	19	F	14	Abs, MJ
34	WC-IGE	20	M	16	Abs
1	DR-IGE	23	F	14	Abs, MJ
2	DR-IGE	19	M	16	IGE
3	DR-IGE	19	F	8	GTCS, Abs
5	DR-IGE	60	F	13	GTCS, Abs
6	DR-IGE	24	M	15	GTC, MJ, abs
7	DR-IGE	21	F	15	GTC, MJ, abs
8	DR-IGE	32	F	23	GTC, MJ
9	DR-IGE	38	M	18	GTC, MJ
10	DR-IGE	67	M	29	GTC, Abs
11	DR-IGE	46	F	7	Abs
13	DR-IGE	20	M	8	GTC
14	DR-IGE	24	F	13	GTC, MJ
15	DR-IGE	35	M	6	GTC
16	DR-IGE	18	M	14	GTC, Abs
17	DR-IGE	39	M	17	GTC
19	DR-IGE	21	M	16	GTC, abs, MJ
20	DR-IGE	36	F	17	GTC
21	DR-IGE	31	F	15	GTC
22	DR-IGE	31	F	16	GTC, MJ, Abs
25	DR-IGE	58	F	15	GTC, Abs
28	DR-IGE	24	M	13	MJ, abs
30	DR-IGE	57	F	7	GTC, abs
33	DR-IGE	57	F	7	GTC, abs

Table 2 displays participant information for the group with IGE. WC-IGE = well controlled IGE, DR-IGE = drug resistant IGE. F = female, M = male, GTC = generalised tonic clonic, MJ = myoclonic jerk, Abs = absence.

For Review Only

Figure 2. Global outcome metrics

i) Networks constructed using absolute values of edges

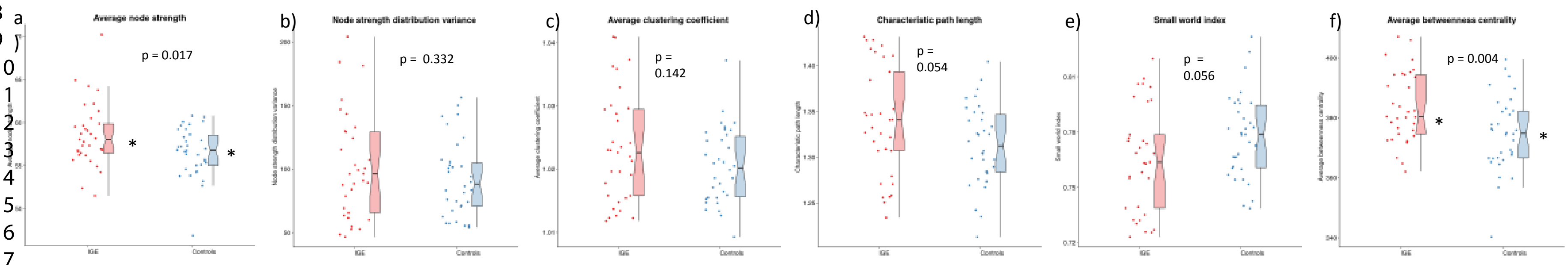

ii) Networks constructed using positively correlated edges

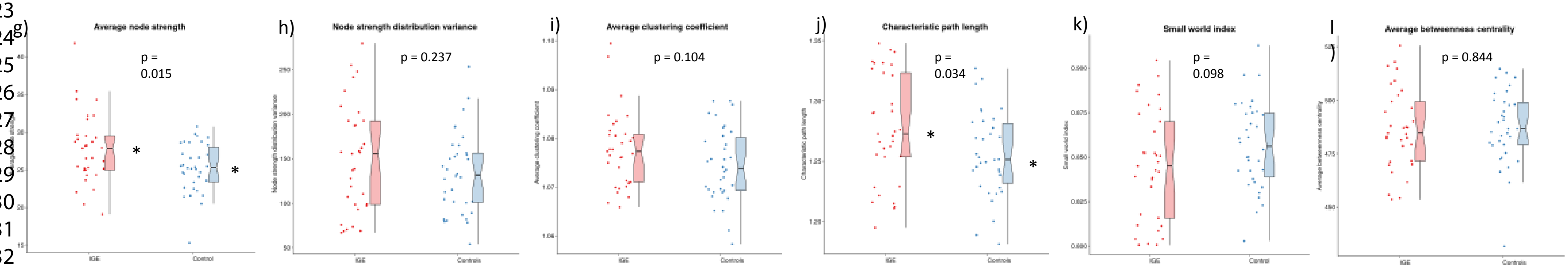

Data is plotted for IGE (both groups combined) and controls. * = statistically significant difference between groups at p < 0.5

Supplementary data 1: All global outcome metric results

A) Networks constructed using absolute values of edges

A1) Three- group comparison (WC-IGE / DR- IGE / Controls)

	3 group comparison			Pairwise comparisons for statistically significant results		
	F	P value	Partial Eta Squared	Groups Compared	P value	Std Error
**Average node strength**	2.97	0.059	0.086			
**Node strength distribution variance**	.988	0.378	0.030			
**Average clustering coefficient**	1.11	0.337	0.034			
**Characteristic path length**	2.11	0.130	0.063			
**Small-world index**	2.13	0.127	0.063			
**Average betweenness centrality**	4.66	0.013	0.129	WC-IGE – Con	0.048	4.455
				DR-IGE – Con	0.057	3.288
				WC-IGE – DR-IGE	1.000	4.742

For Review Only

A2) Two-group comparison (Both IGE groups combined / Controls)

	F	P value	Partial Eta Squared
**Average node strength**	5.956	0.017	0.085
**Node strength distribution variance**	.995	0.332	0.015
**Average clustering coefficient**	2.216	0.142	0.033

**Characteristic path length**	3.864	0.054	0.057
**Small-world index**	3.787	0.056	0.056
**Average betweenness centrality**	8.963	0.004	0.123

B) Networks constructed using positively correlated edges

B1) Three- group comparison (WC-IGE / DR- IGE / Controls)

	3 group comparison		
	F	P value	Partial Eta Squared
**Average node strength**	3.072	0.053	0.89
**Node strength distribution variance**	0.949	.392	0.029
**Average clustering coefficient**	1.815	.171	0.054
**Characteristic path length**	2.689	.076	0.079
**Small-world index**	1.587	.213	0.048
**Average betweenness centrality**	0.405	.669	0.013

For Review Only

B2) Two- group comparison (Both IGE groups combined / Controls)

	F	P value	Partial Eta Squared
**Average node strength**	6.200	0.015	0.088
**Node strength distribution variance**	1.425	0.237	0.022
**Average clustering coefficient**	2.725	0.104	0.041
**Characteristic path length**	4.717	0.034	0.069
**Small-world index**	2.827	0.098	0.042
**Average betweenness centrality**	0.039	.844	0.001

For Review Only
