# Supplementary material for: Functional network topology in drug resistant and well-controlled idiopathic generalized epilepsy: a resting state functional MRI study
Source: Brain Commun. 2021 Aug 26;3(3):fcab196. doi: 10.1093/braincomms/fcab196 (PMC8417840; doi:10.1093/braincomms/fcab196)
Supplement: fcab196_Supplementary_Data [file fcab196_supplementary_data.zip › Supplementary Material.docx]

|  | 3 group comparison | | | | | | Pairwise comparisons for statistically significant results | | |
| --- | --- | --- | --- | --- | --- | --- | --- | --- | --- |
|  | Mean  WC-IGE | Mean DR-IGE | Mean control | F | P value | Partial Eta Squared | Groups  Compared | P value | Std Error |
| **Average node strength** | 58.79 | 58.46 | 56.57 | 2.97 | 0.059 | 0.086 | - | - | - |
| **Node strength distribution variance** | 111.14 | 97.17 | 92.54 | .988 | 0.378 | 0.030 | - | - | - |
| **Average clustering coefficient** | 1.024 | 1.023 | 1.021 | 1.11 | 0.337 | 0.034 | - | - | - |
| **Characteristic path length** | 1.352 | 1.333 | 1.313 | 2.11 | 0.130 | 0.063 | - | - | - |
| **Small-world index** | 0.758 | 0.768 | 0.776 | 2.13 | 0.127 | 0.063 | - | - | - |
| **Average betweenness centrality** | 386.31 | 382.37 | 374.65 | 4.66 | 0.013* | 0.129 | WC-IGE – Con | 0.048* | 4.455 |
|  |  |  |  |  |  |  | DR-IGE – Con | 0.057 | 3.288 |
|  |  |  |  |  |  |  | WC-IGE – DR-IGE | 1.000 | 4.742 |

**Supplementary Material**

Supplementary Table 1. Three group comparison of global metrics for networks constructed using absolute values of edges with a threshold r = 0.25

Supplementary Table 2. Three group comparison of global metrics for networks constructed using positively correlated edges with threshold r = 0.25

|  | 3 group comparison | | | | | |
| --- | --- | --- | --- | --- | --- | --- |
|  | Mean  WC-IGE | Mean DR-IGE | Mean control | F | P value | Partial Eta Squared |
| **Average node strength** | 27.97 | 27.61 | 25.28 | 3.072 | 0.053 | 0.89 |
| **Node strength distribution variance** | 161.22 | 144.31 | 133.53 | 0.949 | 0.392 | 0.029 |
| **Average clustering coefficient** | 1.080 | 1.077 | 1.075 | 1.815 | 0.171 | 0.054 |
| **Characteristic path length** | 1.290 | 1.271 | 1.255 | 2.689 | 0.076 | 0.079 |
| **Small-world index** | 0.838 | 0.848 | 0.857 | 1.587 | 0.213 | 0.048 |
| **Average betweenness centrality** | 492.52 | 484.06 | 487.31 | 0.405 | 0.669 | 0.013 |

Supplementary Table 3. Comparison of global metrics of both IGE groups (combined) with controls for a range of thresholds

|  | **Absolute or positive edge values** | **Threshold** | **Mean value IGE group** | **Standard deviation** | **Mean value controls** | **Standard deviation** | **F statistic** | **P value** | **Effect size** |
| --- | --- | --- | --- | --- | --- | --- | --- | --- | --- |
| **Average node strength** | Absolute | Nil | 72.30 | 5.76 | 69.42 | 4.01 | 5.58 | 0.021* | 0.08 |
|  |  | 0.125 | 48.77 | 4.17 | 46.59 | 3.21 | 5.74 | 0.020* | 0.082 |
|  |  | 0.25 | 58.56 | 3.74 | 56.57 | 2.83 | 5.956 | 0.017* | 0.085 |
|  |  | 0.375 | 66.36 | 18.64 | 64.20 | 2.93 | 6.263 | 0.015* | 0.89 |
|  | Positive | Nil | 43.93 | 4.48 | 41.58 | 4.11 | 5.776 | 0.019* | 0.83 |
|  |  | 0.125 | 38.86 | 4.64 | 36.40 | 0.01 | 5.913 | 0.018* | 0.085 |
|  |  | 0.25 | 27.73 | 4.64 | 25.28 | 3.25 | 6.200 | 0.015* | 0.088 |
|  |  | 0.375 | 17.30 | 3.93 | 15.18 | 2.54 | 6.795 | 0.012* | 0.094 |
| **Node strength distribution variance** | Absolute | Nil | 213.27 | 122.82 | 193.84 | 74.41 | 0.568 | 0.454 | 0.009 |
|  |  | 0.125 | 125.30 | 52.01 | 110.98 | 39.15 | 1.662 | 0.202 | 0.025 |
|  |  | 0.25 | 101.40 | 41.55 | 92.54 | 29.36 | 0.995 | 0.332 | 0.015 |
|  |  | 0.375 | 113.29 | 41.85 | 109.77 | 29.90 | 0.149 | 0.701 | 0.002 |
|  | Positive | Nil | 142.85 | 57.15 | 127.11 | 44.27 | 1.572 | 0.214 | 0.024 |
|  |  | 0.125 | 153.04 | 59.74 | 137.43 | 46.38 | 1.450 | 0.233 | 0.022 |
|  |  | 0.25 | 149.43 | 60.94 | 133.53 | 45.23 | 1.425 | 0.237 | 0.022 |
|  |  | 0.375 | 112.88 | 52.20 | 96.38 | 35.82 | 2.246 | 0.139 | 0.034 |
| **Average clustering coefficient** | Absolute | Nil | 1.016 | 0.007 | 1.016 | 0.005 | 0.091 | 0.764 | 0.001 |
|  |  | 0.125 | 1.081 | 0.212 | 1.078 | 0.015 | 1.662 | 0.202 | 0.025 |
|  |  | 0.25 | 1.023 | 0.009 | 1.021 | 0.006 | 2.216 | 0.142 | 0.033 |
|  |  | 0.375 | 1.012 | 0.004 | 1.011 | 0.003 | 0.508 | 0.579 | 0.008 |
|  | Positive | Nil | 1.142 | 0.024 | 1.141 | 0.017 | 0.006 | 0.214 | 0.024 |
|  |  | 0.125 | 1.096 | 0.010 | 1.095 | 0.008 | 0.345 | 0.559 | 0.005 |
|  |  | 0.25 | 1.078 | 0.008 | 1.075 | 0.008 | 2.725 | 0.104 | 0.041 |
|  |  | 0.375 | 1.058 | 0.010 | 1.052 | 0.007 | 6.844 | 0.011* | 0.097 |
| **Characteristic path length** | Absolute | Nil | 1.222 | 0.423 | 1.206 | 0.318 | 2.788 | 0.100 | 0.042 |
|  |  | 0.125 | 1.384 | 0.061 | 1.361 | 0.451 | 2.308 | 0.134 | 0.035 |
|  |  | 0.25 | 1.339 | 0.060 | 1.314 | 0.044 | 3.864 | 0.054 | 0.057 |
|  |  | 0.375 | 1.240 | 0.046 | 1.219 | 0.035 | 4.461 | 0.039* | 0.065 |
|  | Positive | Nil | 1.373 | 0.059 | 1.354 | 0.044 | 2.386 | 0.127 | 0.036 |
|  |  | 0.125 | 1.345 | 0.054 | 1.326 | 0.041 | 2.873 | 0.095 | 0.043 |
|  |  | 0.25 | 1.277 | 0.452 | 1.256 | 0.035 | 4.717 | 0.034* | 0.069 |
|  |  | 0.375 | 1.119 | 0.033 | 1.173 | 0.254 | 5.852 | 0.018* | 0.084 |
| **Small-world index** | Absolute | Nil | 0.832 | 0.024 | 0.842 | 0.019 | 3.417 | 0.069 | 0.051 |
|  |  | 0.125 | 0.784 | 0.022 | 0.792 | 0.190 | 2.471 | 0.121 | 0.037 |
|  |  | 0.25 | 0.765 | 0.029 | 0.778 | 0.023 | 3.787 | 0.056 | 0.056 |
|  |  | 0.375 | 0.817 | 0.028 | 0.830 | 0.022 | 4.461 | 0.034* | 0.064 |
|  | Positive | Nil | 0.832 | 0.024 | 0.838 | 0.021 | 4.651 | 0.035* | 0.068 |
|  |  | 0.125 | 0.816 | 0.028 | 0.827 | 0.022 | 3.312 | 0.082 | 0.047 |
|  |  | 0.25 | 0.845 | 0.031 | 0.857 | 0.025 | 2.827 | 0.098 | 0.042 |
|  |  | 0.375 | 0.889 | 0.029 | 0.897 | 0.021 | 1.854 | 0.178 | 0.028 |
| **Average betweenness centrality** | Absolute | Nil | 329.095 | 7.291 | 326.610 | 7.290 | 1.905 | 0.172 | 0.029 |
|  |  | 0.125 | 474.672 | 18.654 | 471.660 | 17.043 | 0.436 | 0.511 | 0.007 |
|  |  | 0.25 | 383.562 | 11.798 | 374.654 | 12.318 | 8.963 | 0.004* | 0.123 |
|  |  | 0.375 | 326.761 | 6.331 | 323.174 | 7.488 | 4.535 | 0.041* | 0.064 |
|  | Positive | Nil | 474.687 | 18.653 | 471.680 | 0.022 | 0.435 | 0.512 | 0.007 |
|  |  | 0.125 | 474.689 | 18.652 | 471.684 | 17.040 | 0.434 | 0.512 | 0.007 |
|  |  | 0.25 | 486.626 | 18.834 | 487.307 | 17.142 | 0.039 | 0.844 | 0.001 |
|  |  | 0.375 | 614.977 | 36.192 | 637.1408 | 35.084 | 6.667 | 0.012* | 0.094 |

* statistically significant at p = < 0.05 using one way ANOVA.

Supplementary Figure 1. Frequency of hub nodes in the frontal regions plotted for controls and IGE group (WC-IGE and DR-IGE combined).

Supplementary Figure 2. Frequency of hub nodes in the temporal regions plotted for controls and IGE group (WC-IGE and DR-IGE combined).

).

Supplementary Figure 3. Frequency of hub nodes in the parietal regions plotted for controls and IGE group (WC-IGE and DR-IGE combined).

Supplementary Figure 4. Frequency of hub nodes in the occipital regions plotted for controls and IGE group (WC-IGE and DR-IGE combined).

Supplementary Figure 5. Frequency of hub nodes in the thalamic regions plotted for controls and IGE group (WC-IGE and DR-IGE combined).

Supplementary Table 4. Statistical significance of difference in node strength between IGE group (WC-IGE and DR-IGE combined) and controls for each region

| **AICHA region** | **AICHA region name** | **P value (un-corrected)** | **Mann-Whitney U** | **IGE mean rank** | **Control mean rank** | **Effect size** |
| --- | --- | --- | --- | --- | --- | --- |
| 1 | G_Frontal_Sup-1-L | NS |  |  |  |  |
| 2 | G_Frontal_Sup-1-R | NS |  |  |  |  |
| 3 | G_Frontal_Sup-2-L | NS |  |  |  |  |
| 4 | G_Frontal_Sup-2-R | NS |  |  |  |  |
| 5 | G_Frontal_Sup-3-L | NS |  |  |  |  |
| 6 | G_Frontal_Sup-3-R | NS |  |  |  |  |
| 7 | S_Sup_Frontal-1-L | NS |  |  |  |  |
| 8 | S_Sup_Frontal-1-R | NS |  |  |  |  |
| 9 | S_Sup_Frontal-2-L | 0.009 | 352 | 40.33 | 27.85 | -0.13 |
| 10 | S_Sup_Frontal-2-R | NS |  |  |  |  |
| 11 | S_Sup_Frontal-3-L | 0.004 | 331 | 40.97 | 27.24 | -0.15 |
| 12 | S_Sup_Frontal-3-R | NS |  |  |  |  |
| 13 | S_Sup_Frontal-4-L | NS |  |  |  |  |
| 14 | S_Sup_Frontal-4-R | NS |  |  |  |  |
| 15 | S_Sup_Frontal-5-L | NS |  |  |  |  |
| 16 | S_Sup_Frontal-5-R | NS |  |  |  |  |
| 17 | S_Sup_Frontal-6-L | NS |  |  |  |  |
| 18 | S_Sup_Frontal-6-R | NS |  |  |  |  |
| 19 | G_Frontal_Mid-1-L | NS |  |  |  |  |
| 20 | G_Frontal_Mid-1-R | NS |  |  |  |  |
| 21 | G_Frontal_Mid-2-L | NS |  |  |  |  |
| 22 | G_Frontal_Mid-2-R | NS |  |  |  |  |
| 23 | G_Frontal_Mid-3-L | 0.014 | 365 | 39.94 | 28.24 | -0.13 |
| 24 | G_Frontal_Mid-3-R | NS |  |  |  |  |
| 25 | G_Frontal_Mid-4-L | NS |  |  |  |  |
| 26 | G_Frontal_Mid-4-R | NS |  |  |  |  |
| 27 | G_Frontal_Mid-5-L | NS |  |  |  |  |
| 28 | G_Frontal_Mid-5-R | NS |  |  |  |  |
| 29 | S_Inf_Frontal-1-L | NS |  |  |  |  |
| 30 | S_Inf_Frontal-1-R | NS |  |  |  |  |
| 31 | S_Inf_Frontal-2-L | NS |  |  |  |  |
| 32 | S_Inf_Frontal-2-R | NS |  |  |  |  |
| 33 | G_Frontal_Inf_Tri-1-L | NS |  |  |  |  |
| 34 | G_Frontal_Inf_Tri-1-R | NS |  |  |  |  |
| 35 | G_Frontal_Sup_Orb-1-L | NS |  |  |  |  |
| 36 | G_Frontal_Sup_Orb-1-R | NS |  |  |  |  |
| 37 | G_Frontal_Mid_Orb-1-L | NS |  |  |  |  |
| 38 | G_Frontal_Mid_Orb-1-R | NS |  |  |  |  |
| 39 | G_Frontal_Mid_Orb-2-L | NS |  |  |  |  |
| 40 | G_Frontal_Mid_Orb-2-R | NS |  |  |  |  |
| 41 | G_Frontal_Inf_Orb-1-L | NS |  |  |  |  |
| 42 | G_Frontal_Inf_Orb-1-R 4 | NS |  |  |  |  |
| 43 | G_Frontal_Inf_Orb-2-L | NS |  |  |  |  |
| 44 | G_Frontal_Inf_Orb-2-R | NS |  |  |  |  |
| 45 | S_Orbital-1-L | NS |  |  |  |  |
| 46 | S_Orbital-1-R | NS |  |  |  |  |
| 47 | S_Orbital-2-L | NS |  |  |  |  |
| 48 | S_Orbital-2-R | NS |  |  |  |  |
| 49 | S_Olfactory-1-L | NS |  |  |  |  |
| 50 | S_Olfactory-1-R | NS |  |  |  |  |
| 51 | S_Precentral-1-L | NS |  |  |  |  |
| 52 | S_Precentral-1-R | NS |  |  |  |  |
| 53 | S_Precentral-2-L | NS |  |  |  |  |
| 54 | S_Precentral-2-R | 0.032 | 390 | 39.18 | 28.97 | -0.11 |
| 55 | S_Precentral-3-L | NS |  |  |  |  |
| 56 | S_Precentral-3-R | 0.034 | 392 | 39.12 | 29.03 | -0.11 |
| 57 | S_Precentral-4-L | NS |  |  |  |  |
| 58 | S_Precentral-4-R | NS |  |  |  |  |
| 59 | S_Precentral-5-L | NS |  |  |  |  |
| 60 | S_Precentral-5-R | NS |  |  |  |  |
| 61 | S_Precentral-6-L | NS |  |  |  |  |
| 62 | S_Precentral-6-R | NS |  |  |  |  |
| 63 | S_Rolando-1-L | NS |  |  |  |  |
| 64 | S_Rolando-1-R | NS |  |  |  |  |
| 65 | S_Rolando-2-L | NS |  |  |  |  |
| 66 | S_Rolando-2-R | NS |  |  |  |  |
| 67 | S_Rolando-3-L | NS |  |  |  |  |
| 68 | S_Rolando-3-R | NS |  |  |  |  |
| 69 | S_Rolando-4-L | NS |  |  |  |  |
| 70 | S_Rolando-4-R | NS |  |  |  |  |
| 71 | S_Postcentral-1-L | NS |  |  |  |  |
| 72 | S_Postcentral-1-R | NS |  |  |  |  |
| 73 | S_Postcentral-2-L | NS |  |  |  |  |
| 74 | S_Postcentral-2-R | NS |  |  |  |  |
| 75 | S_Postcentral-3-L | NS |  |  |  |  |
| 76 | S_Postcentral-3-R | NS |  |  |  |  |
| 77 | G_Parietal_Sup-1-L | NS |  |  |  |  |
| 78 | G_Parietal_Sup-1-R | NS |  |  |  |  |
| 79 | G_Parietal_Sup-2-L | NS |  |  |  |  |
| 80 | G_Parietal_Sup-2-R | NS |  |  |  |  |
| 81 | G_Parietal_Sup-3-L | NS |  |  |  |  |
| 82 | G_Parietal_Sup-3-R | NS |  |  |  |  |
| 83 | G_Parietal_Sup-4-L | NS |  |  |  |  |
| 84 | G_Parietal_Sup-4-R | NS |  |  |  |  |
| 85 | G_Parietal_Sup-5-L | NS |  |  |  |  |
| 86 | G_Parietal_Sup-5-R | NS |  |  |  |  |
| 87 | G_Supramarginal-1-L | NS |  |  |  |  |
| 88 | G_Supramarginal-1-R | NS |  |  |  |  |
| 89 | G_SupraMarginal-2-L | NS |  |  |  |  |
| 90 | G_SupraMarginal-2-R | NS |  |  |  |  |
| 91 | G_Supramarginal-3-L | NS |  |  |  |  |
| 92 | G_Supramarginal-3-R | NS |  |  |  |  |
| 93 | G_Supramarginal-4-L | NS |  |  |  |  |
| 94 | G_Supramarginal-4-R | NS |  |  |  |  |
| 95 | G_SupraMarginal-5-L | NS |  |  |  |  |
| 96 | G_SupraMarginal-5-R | NS |  |  |  |  |
| 97 | G_SupraMarginal-6-L | NS |  |  |  |  |
| 98 | G_SupraMarginal-6-R | NS |  |  |  |  |
| 99 | G_SupraMarginal-7-L | NS |  |  |  |  |
| 100 | G_SupraMarginal-7-R | NS |  |  |  |  |
| 101 | G_Angular-1-L | NS |  |  |  |  |
| 102 | G_Angular-1-R | NS |  |  |  |  |
| 103 | G_Angular-2-L | NS |  |  |  |  |
| 104 | G_Angular-2-R | NS |  |  |  |  |
| 105 | G_Angular-3-L | NS |  |  |  |  |
| 106 | G_Angular-3-R | NS |  |  |  |  |
| 107 | G_Parietal_Inf-1-L | NS |  |  |  |  |
| 108 | G_Parietal_Inf-1-R | NS |  |  |  |  |
| 109 | S_Intraparietal-1-L | NS |  |  |  |  |
| 110 | S_Intraparietal-1-R | NS |  |  |  |  |
| 111 | S_Intraparietal-2-L | NS |  |  |  |  |
| 112 | S_Intraparietal-2-R | 0.024 | 381 | 39.45 | 28.71 | -0.12 |
| 113 | S_Intraparietal-3-L | NS |  |  |  |  |
| 114 | S_Intraparietal-3-R | NS |  |  |  |  |
| 115 | S_Intraoccipital-1-L | NS |  |  |  |  |
| 116 | S_Intraoccipital-1-R | NS |  |  |  |  |
| 117 | G_Occipital_Pole-1-L | NS |  |  |  |  |
| 118 | G_Occipital_Pole-1-R | NS |  |  |  |  |
| 119 | G_Occipital_Lat-1-L | NS |  |  |  |  |
| 120 | G_Occipital_Lat-1-R | 0.026 | 384 | 39.36 | 28.79 | -0.11 |
| 121 | G_Occipital_Lat-2-L | NS |  |  |  |  |
| 122 | G_Occipital_Lat-2-R | NS |  |  |  |  |
| 123 | G_Occipital_Lat-3-L | NS |  |  |  |  |
| 124 | G_Occipital_Lat-3-R | NS |  |  |  |  |
| 125 | G_Occipital_Lat-4-L | NS |  |  |  |  |
| 126 | G_Occipital_Lat-4-R | NS |  |  |  |  |
| 127 | G_Occipital_Lat-5-L | NS |  |  |  |  |
| 128 | G_Occipital_Lat-5-R | NS |  |  |  |  |
| 129 | G_Occipital_Sup-1-L | NS |  |  |  |  |
| 130 | G_Occipital_Sup-1-R | NS |  |  |  |  |
| 131 | G_Occipital_Sup-2-L | NS |  |  |  |  |
| 132 | G_Occipital_Sup-2-R | NS |  |  |  |  |
| 133 | G_Occipital_Mid-1-L | NS |  |  |  |  |
| 134 | G_Occipital_Mid-1-R | NS |  |  |  |  |
| 135 | G_Occipital_Mid-2-L | NS |  |  |  |  |
| 136 | G_Occipital_Mid-2-R | NS |  |  |  |  |
| 137 | G_Occipital_Mid-3-L | NS |  |  |  |  |
| 138 | G_Occipital_Mid-3-R | NS |  |  |  |  |
| 139 | G_Occipital_Mid-4-L | NS |  |  |  |  |
| 140 | G_Occipital_Mid-4-R | NS |  |  |  |  |
| 141 | G_Occipital_Inf-1-L | NS |  |  |  |  |
| 142 | G_Occipital_Inf-1-R | NS |  |  |  |  |
| 143 | G_Occipital_Inf-2-L | NS |  |  |  |  |
| 144 | G_Occipital_Inf-2-R | NS |  |  |  |  |
| 145 | G_Insula-anterior-1-L | 0.036 | 394 | 39.06 | 29.09 | -0.11 |
| 146 | G_Insula-anterior-1-R | NS |  |  |  |  |
| 147 | G_Insula-anterior-2-L | NS |  |  |  |  |
| 148 | G_Insula-anterior-2-R | NS |  |  |  |  |
| 149 | G_Insula-anterior-3-L | NS |  |  |  |  |
| 150 | G_Insula-anterior-3-R | 0.024 | 381 | 39.45 | 28.71 | -0.12 |
| 151 | G_Insula-anterior-4-L | NS |  |  |  |  |
| 152 | G_Insula-anterior-4-R | NS |  |  |  |  |
| 153 | G_Insula-anterior-5-L | NS |  |  |  |  |
| 154 | G_Insula-anterior-5-R | NS |  |  |  |  |
| 155 | G_Insula-posterior-1-L | NS |  |  |  |  |
| 156 | G_Insula-posterior-1-R | NS |  |  |  |  |
| 157 | G_Rolandic_Oper-1-L | NS |  |  |  |  |
| 158 | G_Rolandic_Oper-1-R | NS |  |  |  |  |
| 159 | G_Rolandic_Oper-2-L | NS |  |  |  |  |
| 160 | G_Rolandic_Oper-2-R | NS |  |  |  |  |
| 161 | G_Temporal_Sup-1-L | NS |  |  |  |  |
| 162 | G_Temporal_Sup-1-R | NS |  |  |  |  |
| 163 | G_Temporal_Sup-2-L | NS |  |  |  |  |
| 164 | G_Temporal_Sup-2-R | NS |  |  |  |  |
| 165 | G_Temporal_Sup-3-L | NS |  |  |  |  |
| 166 | G_Temporal_Sup-3-R | NS |  |  |  |  |
| 167 | G_Temporal_Sup-4-L | NS |  |  |  |  |
| 168 | G_Temporal_Sup-4-R | NS |  |  |  |  |
| 169 | S_Sup_Temporal-1-L | NS |  |  |  |  |
| 170 | S_Sup_Temporal-1-R | NS |  |  |  |  |
| 171 | S_Sup_Temporal-2-L | NS |  |  |  |  |
| 172 | S_Sup_Temporal-2-R | NS |  |  |  |  |
| 173 | S_Sup_Temporal-3-L | NS |  |  |  |  |
| 174 | S_Sup_Temporal-3-R | 0.021 | 377.5 | 39.56 | 28.6 | -0.12 |
| 175 | S_Sup_Temporal-4-L | NS |  |  |  |  |
| 176 | S_Sup_Temporal-4-R | NS |  |  |  |  |
| 177 | S_Sup_Temporal-5-L | NS |  |  |  |  |
| 178 | S_Sup_Temporal-5-R | NS |  |  |  |  |
| 179 | G_Temporal_Mid-1-L | NS |  |  |  |  |
| 180 | G_Temporal_Mid-1-R | NS |  |  |  |  |
| 181 | G_Temporal_Mid-2-L | NS |  |  |  |  |
| 182 | G_Temporal_Mid-2-R | NS |  |  |  |  |
| 183 | G_Temporal_Mid-3-L | NS |  |  |  |  |
| 184 | G_Temporal_Mid-3-R | NS |  |  |  |  |
| 185 | G_Temporal_Mid-4-L | NS |  |  |  |  |
| 186 | G_Temporal_Mid-4-R | NS |  |  |  |  |
| 187 | G_Temporal_Inf-1-L | NS |  |  |  |  |
| 188 | G_Temporal_Inf-1-R | NS |  |  |  |  |
| 189 | G_Temporal_Inf-2-L | NS |  |  |  |  |
| 190 | G_Temporal_Inf-2-R | NS |  |  |  |  |
| 191 | G_Temporal_Inf-3-L | NS |  |  |  |  |
| 192 | G_Temporal_Inf-3-R | NS |  |  |  |  |
| 193 | G_Temporal_Inf-4-L | NS |  |  |  |  |
| 194 | G_Temporal_Inf-4-R | NS |  |  |  |  |
| 195 | G_Temporal_Inf-5-L | NS |  |  |  |  |
| 196 | G_Temporal_Inf-5-R | NS |  |  |  |  |
| 197 | G_Temporal_Pole_Sup-1-L | NS |  |  |  |  |
| 198 | G_Temporal_Pole_Sup-1-R | NS |  |  |  |  |
| 199 | G_Temporal_Pole_Sup-2-L | NS |  |  |  |  |
| 200 | G_Temporal_Pole_Sup-2-R | NS |  |  |  |  |
| 201 | G_Temporal_Pole_Mid-1-L | NS |  |  |  |  |
| 202 | G_Temporal_Pole_Mid-1-R | NS |  |  |  |  |
| 203 | G_Temporal_Pole_Mid-2-L | NS |  |  |  |  |
| 204 | G_Temporal_Pole_Mid-2-R | 0.022 | 378 | 39.55 | 28.62 | -0.12 |
| 205 | G_Temporal_Pole_Mid-3-L | NS |  |  |  |  |
| 206 | G_Temporal_Pole_Mid-3-R | NS |  |  |  |  |
| 207 | G_Frontal_Sup_Medial-1-L | NS |  |  |  |  |
| 208 | G_Frontal_Sup_Medial-1-R | NS |  |  |  |  |
| 209 | G_Frontal_Sup_Medial-2-L | NS |  |  |  |  |
| 210 | G_Frontal_Sup_Medial-2-R | NS |  |  |  |  |
| 211 | G_Frontal_Sup_Medial-3-L | NS |  |  |  |  |
| 212 | G_Frontal_Sup_Medial-3-R | NS | 393 | 39.09 | 29.06 | -0.11 |
| 213 | S_Anterior_Rostral-1-L | NS |  |  |  |  |
| 214 | S_Anterior_Rostral-1-R | NS |  |  |  |  |
| 215 | G_Frontal_Med_Orb-1-L | NS |  |  |  |  |
| 216 | G_Frontal_Med_Orb-1-R | NS |  |  |  |  |
| 217 | G_Frontal_Med_Orb-2-L | NS |  |  |  |  |
| 218 | G_Frontal_Med_Orb-2-R | NS |  |  |  |  |
| 219 | G_subcallosal-1-L | NS |  |  |  |  |
| 220 | G_subcallosal-1-R | NS |  |  |  |  |
| 221 | G_Supp_Motor_Area-1-L | NS |  |  |  |  |
| 222 | G_Supp_Motor_Area-1-R | NS |  |  |  |  |
| 223 | G_Supp_Motor_Area-2-L | 0.031 | 389 | 39.21 | 28.94 | -0.11 |
| 224 | G_Supp_Motor_Area-2-R | 0.028 | 386 | 39.3 | 28.85 | -0.11 |
| 225 | G_Supp_Motor_Area-3-L | 0.001 | 302 | 41.85 | 26.38 | -0.17 |
| 226 | G_Supp_Motor_Area-3-R | NS |  |  |  |  |
| 227 | S_Cingulate-1-L | NS |  |  |  |  |
| 228 | S_Cingulate-1-R | NS |  |  |  |  |
| 229 | S_Cingulate-2-L | 0.039 | 396 | 39 | 29.15 | -0.12 |
| 230 | S_Cingulate-2-R | NS |  |  |  |  |
| 231 | S_Cingulate-3-L | NS |  |  |  |  |
| 232 | S_Cingulate-3-R | NS |  |  |  |  |
| 233 | S_Cingulate-4-L | NS |  |  |  |  |
| 234 | S_Cingulate-4-R | NS |  |  |  |  |
| 235 | S_Cingulate-5-L | 0.045 | 401 | 38.85 | 29.29 | -0.10 |
| 236 | S_Cingulate-5-R | NS |  |  |  |  |
| 237 | S_Cingulate-6-L | NS |  |  |  |  |
| 238 | S_Cingulate-6-R | NS |  |  |  |  |
| 239 | S_Cingulate-7-L | NS |  |  |  |  |
| 240 | S_Cingulate-7-R | NS |  |  |  |  |
| 241 | G_Cingulum_Ant-1-L | NS |  |  |  |  |
| 242 | G_Cingulum_Ant-1-R | NS |  |  |  |  |
| 243 | G_Cingulum_Ant-2-L | 0.029 | 387 | 39.27 | 28.88 | -0.11 |
| 244 | G_Cingulum_Ant-2-R | NS |  |  |  |  |
| 245 | G_Cingulum_Mid-1-L | NS |  |  |  |  |
| 246 | G_Cingulum_Mid-1-R | NS |  |  |  |  |
| 247 | G_Cingulum_Mid-2-L | NS |  |  |  |  |
| 248 | G_Cingulum_Mid-2-R | 0.022 | 378 | 39.55 | 28.62 | -0.12 |
| 249 | G_Cingulum_Mid-3-L | NS |  |  |  |  |
| 250 | G_Cingulum_Mid-3-R | NS |  |  |  |  |
| 251 | G_Cingulum_Post-1-L | NS | 445 | 37.52 | 30.59 | -0.14 |
| 252 | G_Cingulum_Post-1-R | NS |  |  |  |  |
| 253 | G_Cingulum_Post-2-L | NS |  |  |  |  |
| 254 | G_Cingulum_Post-2-R | NS |  |  |  |  |
| 255 | G_Cingulum_Post-3-L | NS |  |  |  |  |
| 256 | G_Cingulum_Post-3-R | 0.008 | 350 | 40.39 | 27.79 | -0.13 |
| 257 | G_Paracentral_Lobule-1-L | NS |  |  |  |  |
| 258 | G_Paracentral_Lobule-1-R | NS |  |  |  |  |
| 259 | G_Paracentral_Lobule-2-L | NS |  |  |  |  |
| 260 | G_Paracentral_Lobule-2-R | NS |  |  |  |  |
| 261 | G_Paracentral_Lobule-3-L | NS |  |  |  |  |
| 262 | G_Paracentral_Lobule-3-R | NS |  |  |  |  |
| 263 | G_Paracentral_Lobule-4-L | NS |  |  |  |  |
| 264 | G_Paracentral_Lobule-4-R | NS |  |  |  |  |
| 265 | G_Precuneus-1-L | NS |  |  |  |  |
| 266 | G_Precuneus-1-R | NS |  |  |  |  |
| 267 | G_Precuneus-2-L | NS |  |  |  |  |
| 268 | G_Precuneus-2-R | NS |  |  |  |  |
| 269 | G_Precuneus-3-L | NS |  |  |  |  |
| 270 | G_Precuneus-3-R | NS |  |  |  |  |
| 271 | G_Precuneus-4-L | NS |  |  |  |  |
| 272 | G_Precuneus-4-R | NS |  |  |  |  |
| 273 | G_Precuneus-5-L | NS |  |  |  |  |
| 274 | G_Precuneus-5-R | NS |  |  |  |  |
| 275 | G_Precuneus-6-L | NS |  |  |  |  |
| 276 | G_Precuneus-6-R | NS |  |  |  |  |
| 277 | G_Precuneus-7-L | NS |  |  |  |  |
| 278 | G_Precuneus-7-R | NS |  |  |  |  |
| 279 | G_Precuneus-8-L | NS |  |  |  |  |
| 280 | G_Precuneus-8-R | NS |  |  |  |  |
| 281 | G_Precuneus-9-L | NS |  |  |  |  |
| 282 | G_Precuneus-9-R | NS |  |  |  |  |
| 283 | S_Parietooccipital-1-L | NS |  |  |  |  |
| 284 | S_Parietooccipital-1-R | 0.012 | 360 | 40.09 | 28.09 | -0.14 |
| 285 | S_Parietooccipital-2-L | NS |  |  |  |  |
| 286 | S_Parietooccipital-2-R | NS |  |  |  |  |
| 287 | S_Parietooccipital-3-L | NS |  |  |  |  |
| 288 | S_Parietooccipital-3-R | NS |  |  |  |  |
| 289 | S_Parietooccipital-4-L | NS |  |  |  |  |
| 290 | S_Parietooccipital-4-R | NS |  |  |  |  |
| 291 | S_Parietooccipital-5-L | NS |  |  |  |  |
| 292 | S_Parietooccipital-5-R | NS |  |  |  |  |
| 293 | S_Parietooccipital-6-L | NS |  |  |  |  |
| 294 | S_Parietooccipital-6-R | NS |  |  |  |  |
| 295 | G_Cuneus-1-L | NS |  |  |  |  |
| 296 | G_Cuneus-1-R | NS |  |  |  |  |
| 297 | G_Cuneus-2-L | NS |  |  |  |  |
| 298 | G_Cuneus-2-R | NS |  |  |  |  |
| 299 | G_Calcarine-1-L | NS |  |  |  |  |
| 300 | G_Calcarine-1-R | NS |  |  |  |  |
| 301 | G_Calcarine-2-L | NS |  |  |  |  |
| 302 | G_Calcarine-2-R | NS |  |  |  |  |
| 303 | G_Calcarine-3-L | 0.028 | 386 | 39.3 | 28.85 | -0.11 |
| 304 | G_Calcarine-3-R | 0.029 | 387 | 39.27 | 28.88 | -0.11 |
| 305 | G_Lingual-1-L | NS |  |  |  |  |
| 306 | G_Lingual-1-R | 0.017 | 371 | 39.76 | 28.41 | -0.12 |
| 307 | G_Lingual-2-L | NS |  |  |  |  |
| 308 | G_Lingual-2-R | NS |  |  |  |  |
| 309 | G_Lingual-3-L | NS |  |  |  |  |
| 310 | G_Lingual-3-R | NS |  |  |  |  |
| 311 | G_Lingual-4-L | NS |  |  |  |  |
| 312 | G_Lingual-4-R | NS |  |  |  |  |
| 313 | G_Lingual-5-L | NS |  |  |  |  |
| 314 | G_Lingual-5-R | 0.027 | 385 | 39.33 | 28.82 | -0.11 |
| 315 | G_Lingual-6-L | NS |  |  |  |  |
| 316 | G_Lingual-6-R | NS |  |  |  |  |
| 317 | G_Hippocampus-1-L | 0.005 | 337 | 40.79 | 27.41 | -0.14 |
| 318 | G_Hippocampus-1-R | NS |  |  |  |  |
| 319 | G_Hippocampus-2-L | NS |  |  |  |  |
| 320 | G_Hippocampus-2-R | 0.001 | 293 | 42.12 | 26.12 | -0.17 |
| 321 | G_ParaHippocampal-1-L | 0.024 | 381 | 39.45 | 28.71 | -0.12 |
| 322 | G_ParaHippocampal-1-R | NS |  |  |  |  |
| 323 | G_ParaHippocampal-2-L | NS |  |  |  |  |
| 324 | G_ParaHippocampal-2-R | NS |  |  |  |  |
| 325 | G_ParaHippocampal-3-L | NS |  |  |  |  |
| 326 | G_ParaHippocampal-3-R | NS |  |  |  |  |
| 327 | G_ParaHippocampal-4-L | 0.022 | 378 | 39.55 | 28.62 | -0.12 |
| 328 | G_ParaHippocampal-4-R | NS |  |  |  |  |
| 329 | G_ParaHippocampal-5-L | NS |  |  |  |  |
| 330 | G_ParaHippocampal-5-R | NS |  |  |  |  |
| 331 | G_Fusiform-1-L | NS |  |  |  |  |
| 332 | G_Fusiform-1-R | 0.01 | 356 | 40.21 | 27.97 | -0.13 |
| 333 | G_Fusiform-2-L | NS |  |  |  |  |
| 334 | G_Fusiform-2-R | NS |  |  |  |  |
| 335 | G_Fusiform-3-L | NS |  |  |  |  |
| 336 | G_Fusiform-3-R | NS |  |  |  |  |
| 337 | G_Fusiform-4-L | NS |  |  |  |  |
| 338 | G_Fusiform-4-R | NS |  |  |  |  |
| 339 | G_Fusiform-5-L | NS |  |  |  |  |
| 340 | G_Fusiform-5-R | 0.034 | 392 | 39.12 | 29.03 | -0.11 |
| 341 | G_Fusiform-6-L | NS |  |  |  |  |
| 342 | G_Fusiform-6-R | 0.022 | 378 | 39.55 | 28.62 | -0.12 |
| 343 | G_Fusiform-7-L | NS |  |  |  |  |
| 344 | G_Fusiform-7-R | 0.007 | 346 | 40.52 | 27.68 | -0.14 |
| 345 | N_Amygdala-1-L | NS |  |  |  |  |
| 346 | N_Amygdala-1-R | NS |  |  |  |  |
| 347 | N_Caudate-1-L | NS |  |  |  |  |
| 348 | N_Caudate-1-R | 0.006 | 341 | 40.67 | 27.53 | -0.14 |
| 349 | N_Caudate-2-L | NS |  |  |  |  |
| 350 | N_Caudate-2-R | 0.015 | 367 | 39.88 | 28.29 | -0.12 |
| 351 | N_Caudate-3-L | NS |  |  |  |  |
| 352 | N_Caudate-3-R | NS |  |  |  |  |
| 353 | N_Caudate-4-L | NS |  |  |  |  |
| 354 | N_Caudate-4-R | NS |  |  |  |  |
| 355 | N_Caudate-5-L | NS |  |  |  |  |
| 356 | N_Caudate-5-R | NS |  |  |  |  |
| 357 | N_Caudate-6-L | NS |  |  |  |  |
| 358 | N_Caudate-6-R | NS |  |  |  |  |
| 359 | N_Caudate-7-L | NS |  |  |  |  |
| 360 | N_Caudate-7-R | NS |  |  |  |  |
| 361 | N_Pallidum-1-L | NS |  |  |  |  |
| 362 | N_Pallidum-1-R | NS |  |  |  |  |
| 363 | N_Putamen-2-L | NS |  |  |  |  |
| 364 | N_Putamen-2-R | NS |  |  |  |  |
| 365 | N_Putamen-3-L | NS |  |  |  |  |
| 366 | N_Putamen-3-R | NS |  |  |  |  |
| 367 | N_Thalamus-1-L | NS |  |  |  |  |
| 368 | N_Thalamus-1-R | NS |  |  |  |  |
| 369 | N_Thalamus-2-L | NS |  |  |  |  |
| 370 | N_Thalamus-2-R | NS |  |  |  |  |
| 371 | N_Thalamus-3-L | NS |  |  |  |  |
| 372 | N_Thalamus-3-R | NS |  |  |  |  |
| 373 | N_Thalamus-4-L | 0.016 | 368 | 39.85 | 28.32 | -0.12 |
| 374 | N_Thalamus-4-R | NS |  |  |  |  |
| 375 | N_Thalamus-5-L | NS |  |  |  |  |
| 376 | N_Thalamus-5-R | NS |  |  |  |  |
| 377 | N_Thalamus-6-L | NS |  |  |  |  |
| 378 | N_Thalamus-6-R | NS |  |  |  |  |
| 379 | N_Thalamus-7-L | NS |  |  |  |  |
| 380 | N_Thalamus-7-R | NS |  |  |  |  |
| 381 | N_Thalamus-8-L | NS |  |  |  |  |
| 382 | N_Thalamus-8-R | NS |  |  |  |  |
| 383 | N_Thalamus-9- L | NS |  |  |  |  |
| 384 | N_Thalamus-9-R | NS |  |  |  |  |

NS = not significant at p < 0.05. Effect size calculated as r=$Z/\surd N$. All results displayed to two decimal points.

Supplementary Table 5. Statistical significance of difference in betweenness centrality between controls and people with IGE for each region

| **AICHA region** | **AICHA region name** | **p value (uncorrected)** | **Mann-Whitney U** | **IGE mean rank** | **Control mean rank** | **Effect size** |
| --- | --- | --- | --- | --- | --- | --- |
| 1 | G_Frontal_Sup-1-L | NS |  |  |  |  |
| 2 | G_Frontal_Sup-1-R | NS |  |  |  |  |
| 3 | G_Frontal_Sup-2-L | NS |  |  |  |  |
| 4 | G_Frontal_Sup-2-R | NS |  |  |  |  |
| 5 | G_Frontal_Sup-3-L | NS |  |  |  |  |
| 6 | G_Frontal_Sup-3-R | NS |  |  |  |  |
| 7 | S_Sup_Frontal-1-L | NS |  |  |  |  |
| 8 | S_Sup_Frontal-1-R | NS |  |  |  |  |
| 9 | S_Sup_Frontal-2-L | NS |  |  |  |  |
| 10 | S_Sup_Frontal-2-R | NS |  |  |  |  |
| 11 | S_Sup_Frontal-3-L | NS |  |  |  |  |
| 12 | S_Sup_Frontal-3-R | NS |  |  |  |  |
| 13 | S_Sup_Frontal-4-L | NS |  |  |  |  |
| 14 | S_Sup_Frontal-4-R | NS |  |  |  |  |
| 15 | S_Sup_Frontal-5-L | NS |  |  |  |  |
| 16 | S_Sup_Frontal-5-R | NS |  |  |  |  |
| 17 | S_Sup_Frontal-6-L | NS |  |  |  |  |
| 18 | S_Sup_Frontal-6-R | 0.005 | 339 | 40.73 | 28.32 | -0.14 |
| 19 | G_Frontal_Mid-1-L | NS |  |  |  |  |
| 20 | G_Frontal_Mid-1-R | NS |  |  |  |  |
| 21 | G_Frontal_Mid-2-L | NS |  |  |  |  |
| 22 | G_Frontal_Mid-2-R | NS |  |  |  |  |
| 23 | G_Frontal_Mid-3-L | NS |  |  |  |  |
| 24 | G_Frontal_Mid-3-R | NS |  |  |  |  |
| 25 | G_Frontal_Mid-4-L | NS |  |  |  |  |
| 26 | G_Frontal_Mid-4-R | NS |  |  |  |  |
| 27 | G_Frontal_Mid-5-L | NS |  |  |  |  |
| 28 | G_Frontal_Mid-5-R | NS |  |  |  |  |
| 29 | S_Inf_Frontal-1-L | NS |  |  |  |  |
| 30 | S_Inf_Frontal-1-R | NS |  |  |  |  |
| 31 | S_Inf_Frontal-2-L | NS |  |  |  |  |
| 32 | S_Inf_Frontal-2-R | NS |  |  |  |  |
| 33 | G_Frontal_Inf_Tri-1-L | NS |  |  |  |  |
| 34 | G_Frontal_Inf_Tri-1-R | NS |  |  |  |  |
| 35 | G_Frontal_Sup_Orb-1-L | NS |  |  |  |  |
| 36 | G_Frontal_Sup_Orb-1-R | NS |  |  |  |  |
| 37 | G_Frontal_Mid_Orb-1-L | NS |  |  |  |  |
| 38 | G_Frontal_Mid_Orb-1-R | NS |  |  |  |  |
| 39 | G_Frontal_Mid_Orb-2-L | 0.017 | 751 | 28.24 | 40.6 | 0.15 |
| 40 | G_Frontal_Mid_Orb-2-R | NS |  |  |  |  |
| 41 | G_Frontal_Inf_Orb-1-L | NS |  |  |  |  |
| 42 | G_Frontal_Inf_Orb-1-R 4 | NS |  |  |  |  |
| 43 | G_Frontal_Inf_Orb-2-L | NS |  |  |  |  |
| 44 | G_Frontal_Inf_Orb-2-R | NS |  |  |  |  |
| 45 | S_Orbital-1-L | NS |  |  |  |  |
| 46 | S_Orbital-1-R | NS |  |  |  |  |
| 47 | S_Orbital-2-L | NS |  |  |  |  |
| 48 | S_Orbital-2-R | NS |  |  |  |  |
| 49 | S_Olfactory-1-L | 0.031 | 733 | 28.97 | 39.56 | 0.11 |
| 50 | S_Olfactory-1-R | NS |  |  |  |  |
| 51 | S_Precentral-1-L | NS |  |  |  |  |
| 52 | S_Precentral-1-R | 0.042 | 399 | 38.91 | 29.41 | -0.10 |
| 53 | S_Precentral-2-L | 0.027 | 284.5 | 39.35 | 29.75 | -0.11 |
| 54 | S_Precentral-2-R | 0.026 | 383 | 39.39 | 29 | -0.11 |
| 55 | S_Precentral-3-L | NS |  |  |  |  |
| 56 | S_Precentral-3-R | NS |  |  |  |  |
| 57 | S_Precentral-4-L | NS |  |  |  |  |
| 58 | S_Precentral-4-R | NS |  |  |  |  |
| 59 | S_Precentral-5-L | 0.046 | 720 | 29.18 | 36.68 | 0.10 |
| 60 | S_Precentral-5-R | NS |  |  |  |  |
| 61 | S_Precentral-6-L | NS |  |  |  |  |
| 62 | S_Precentral-6-R | NS |  |  |  |  |
| 63 | S_Rolando-1-L | 0.008 | 771.5 | 27.62 | 40.25 | 0.13 |
| 64 | S_Rolando-1-R | NS |  |  |  |  |
| 65 | S_Rolando-2-L | NS |  |  |  |  |
| 66 | S_Rolando-2-R | NS |  |  |  |  |
| 67 | S_Rolando-3-L | NS |  |  |  |  |
| 68 | S_Rolando-3-R | NS |  |  |  |  |
| 69 | S_Rolando-4-L | NS |  |  |  |  |
| 70 | S_Rolando-4-R | NS |  |  |  |  |
| 71 | S_Postcentral-1-L | NS |  |  |  |  |
| 72 | S_Postcentral-1-R | NS |  |  |  |  |
| 73 | S_Postcentral-2-L | 0.007 | 776.5 | 27.47 | 41.25 | 0.14 |
| 74 | S_Postcentral-2-R | NS |  |  |  |  |
| 75 | S_Postcentral-3-L | 0.007 | 777 | 27.45 | 41.35 | 0.14 |
| 76 | S_Postcentral-3-R | NS |  |  |  |  |
| 77 | G_Parietal_Sup-1-L | NS |  |  |  |  |
| 78 | G_Parietal_Sup-1-R | NS |  |  |  |  |
| 79 | G_Parietal_Sup-2-L | NS |  |  |  |  |
| 80 | G_Parietal_Sup-2-R | NS |  |  |  |  |
| 81 | G_Parietal_Sup-3-L | NS |  |  |  |  |
| 82 | G_Parietal_Sup-3-R | NS |  |  |  |  |
| 83 | G_Parietal_Sup-4-L | NS |  |  |  |  |
| 84 | G_Parietal_Sup-4-R | NS |  |  |  |  |
| 85 | G_Parietal_Sup-5-L | NS |  |  |  |  |
| 86 | G_Parietal_Sup-5-R | NS |  |  |  |  |
| 87 | G_Supramarginal-1-L | NS |  |  |  |  |
| 88 | G_Supramarginal-1-R | NS |  |  |  |  |
| 89 | G_SupraMarginal-2-L | NS |  |  |  |  |
| 90 | G_SupraMarginal-2-R | NS |  |  |  |  |
| 91 | G_Supramarginal-3-L | NS |  |  |  |  |
| 92 | G_Supramarginal-3-R | NS |  |  |  |  |
| 93 | G_Supramarginal-4-L | NS |  |  |  |  |
| 94 | G_Supramarginal-4-R | NS |  |  |  |  |
| 95 | G_SupraMarginal-5-L | NS |  |  |  |  |
| 96 | G_SupraMarginal-5-R | NS |  |  |  |  |
| 97 | G_SupraMarginal-6-L | NS |  |  |  |  |
| 98 | G_SupraMarginal-6-R | NS |  |  |  |  |
| 99 | G_SupraMarginal-7-L | NS |  |  |  |  |
| 100 | G_SupraMarginal-7-R | NS |  |  |  |  |
| 101 | G_Angular-1-L | NS |  |  |  |  |
| 102 | G_Angular-1-R | 0.014 | 756.5 | 28.08 | 39.75 | 0.13 |
| 103 | G_Angular-2-L | 0.04 | 724.5 | 29.05 | 39.72 | 0.10 |
| 104 | G_Angular-2-R | NS |  |  |  |  |
| 105 | G_Angular-3-L | NS |  |  |  |  |
| 106 | G_Angular-3-R | NS |  |  |  |  |
| 107 | G_Parietal_Inf-1-L | NS |  |  |  |  |
| 108 | G_Parietal_Inf-1-R | NS |  |  |  |  |
| 109 | S_Intraparietal-1-L | NS |  |  |  |  |
| 110 | S_Intraparietal-1-R | NS |  |  |  |  |
| 111 | S_Intraparietal-2-L | NS |  |  |  |  |
| 112 | S_Intraparietal-2-R | NS |  |  |  |  |
| 113 | S_Intraparietal-3-L | NS |  |  |  |  |
| 114 | S_Intraparietal-3-R | NS |  |  |  |  |
| 115 | S_Intraoccipital-1-L | NS |  |  |  |  |
| 116 | S_Intraoccipital-1-R | NS |  |  |  |  |
| 117 | G_Occipital_Pole-1-L | NS |  |  |  |  |
| 118 | G_Occipital_Pole-1-R | NS |  |  |  |  |
| 119 | G_Occipital_Lat-1-L | NS |  |  |  |  |
| 120 | G_Occipital_Lat-1-R | NS |  |  |  |  |
| 121 | G_Occipital_Lat-2-L | NS |  |  |  |  |
| 122 | G_Occipital_Lat-2-R | NS |  |  |  |  |
| 123 | G_Occipital_Lat-3-L | NS |  |  |  |  |
| 124 | G_Occipital_Lat-3-R | NS |  |  |  |  |
| 125 | G_Occipital_Lat-4-L | NS |  |  |  |  |
| 126 | G_Occipital_Lat-4-R | NS |  |  |  |  |
| 127 | G_Occipital_Lat-5-L | NS |  |  |  |  |
| 128 | G_Occipital_Lat-5-R | NS |  |  |  |  |
| 129 | G_Occipital_Sup-1-L | 0.004 | 789 | 27.09 | 41.35 | 0.15 |
| 130 | G_Occipital_Sup-1-R | NS |  |  |  |  |
| 131 | G_Occipital_Sup-2-L | NS |  |  |  |  |
| 132 | G_Occipital_Sup-2-R | NS |  |  |  |  |
| 133 | G_Occipital_Mid-1-L | NS |  |  |  |  |
| 134 | G_Occipital_Mid-1-R | NS |  |  |  |  |
| 135 | G_Occipital_Mid-2-L | NS |  |  |  |  |
| 136 | G_Occipital_Mid-2-R | NS |  |  |  |  |
| 137 | G_Occipital_Mid-3-L | NS |  |  |  |  |
| 138 | G_Occipital_Mid-3-R | NS |  |  |  |  |
| 139 | G_Occipital_Mid-4-L | NS |  |  |  |  |
| 140 | G_Occipital_Mid-4-R | NS |  |  |  |  |
| 141 | G_Occipital_Inf-1-L | NS |  |  |  |  |
| 142 | G_Occipital_Inf-1-R | NS |  |  |  |  |
| 143 | G_Occipital_Inf-2-L | 0.023 | 742.5 | 28.5 | 39.34 | 0.12 |
| 144 | G_Occipital_Inf-2-R | NS |  |  |  |  |
| 145 | G_Insula-anterior-1-L | NS |  |  |  |  |
| 146 | G_Insula-anterior-1-R | 0.017 | 750.5 | 28.26 | 39.57 | 0.12 |
| 147 | G_Insula-anterior-2-L | NS |  |  |  |  |
| 148 | G_Insula-anterior-2-R | NS |  |  |  |  |
| 149 | G_Insula-anterior-3-L | NS |  |  |  |  |
| 150 | G_Insula-anterior-3-R | NS |  |  |  |  |
| 151 | G_Insula-anterior-4-L | NS |  |  |  |  |
| 152 | G_Insula-anterior-4-R | 0.043 | 399.5 | 38.89 | 29.25 | -0.10 |
| 153 | G_Insula-anterior-5-L | NS |  |  |  |  |
| 154 | G_Insula-anterior-5-R | NS |  |  |  |  |
| 155 | G_Insula-posterior-1-L | NS |  |  |  |  |
| 156 | G_Insula-posterior-1-R | NS |  |  |  |  |
| 157 | G_Rolandic_Oper-1-L | NS |  |  |  |  |
| 158 | G_Rolandic_Oper-1-R | 0.034 | 391.5 | 39.14 | 29.16 | -0.11 |
| 159 | G_Rolandic_Oper-2-L | NS |  |  |  |  |
| 160 | G_Rolandic_Oper-2-R | NS |  |  |  |  |
| 161 | G_Temporal_Sup-1-L | NS |  |  |  |  |
| 162 | G_Temporal_Sup-1-R | NS |  |  |  |  |
| 163 | G_Temporal_Sup-2-L | NS |  |  |  |  |
| 164 | G_Temporal_Sup-2-R | NS |  |  |  |  |
| 165 | G_Temporal_Sup-3-L | NS |  |  |  |  |
| 166 | G_Temporal_Sup-3-R | NS |  |  |  |  |
| 167 | G_Temporal_Sup-4-L | NS |  |  |  |  |
| 168 | G_Temporal_Sup-4-R | 0.048 | 403.5 | 38.77 | 29.72 | -0.10 |
| 169 | S_Sup_Temporal-1-L | NS |  |  |  |  |
| 170 | S_Sup_Temporal-1-R | NS |  |  |  |  |
| 171 | S_Sup_Temporal-2-L | NS |  |  |  |  |
| 172 | S_Sup_Temporal-2-R | NS |  |  |  |  |
| 173 | S_Sup_Temporal-3-L | NS |  |  |  |  |
| 174 | S_Sup_Temporal-3-R | NS |  |  |  |  |
| 175 | S_Sup_Temporal-4-L | NS |  |  |  |  |
| 176 | S_Sup_Temporal-4-R | NS |  |  |  |  |
| 177 | S_Sup_Temporal-5-L | NS |  |  |  |  |
| 178 | S_Sup_Temporal-5-R | NS |  |  |  |  |
| 179 | G_Temporal_Mid-1-L | NS |  |  |  |  |
| 180 | G_Temporal_Mid-1-R | NS |  |  |  |  |
| 181 | G_Temporal_Mid-2-L | 0.012 | 761.5 | 27.92 | 40.09 | 0.13 |
| 182 | G_Temporal_Mid-2-R | NS |  |  |  |  |
| 183 | G_Temporal_Mid-3-L | NS |  |  |  |  |
| 184 | G_Temporal_Mid-3-R | NS |  |  |  |  |
| 185 | G_Temporal_Mid-4-L | NS |  |  |  |  |
| 186 | G_Temporal_Mid-4-R | NS |  |  |  |  |
| 187 | G_Temporal_Inf-1-L | 0.042 | 723 | 29.09 | 39.65 | 0.10 |
| 188 | G_Temporal_Inf-1-R | NS |  |  |  |  |
| 189 | G_Temporal_Inf-2-L | NS |  |  |  |  |
| 190 | G_Temporal_Inf-2-R | NS |  |  |  |  |
| 191 | G_Temporal_Inf-3-L | NS |  |  |  |  |
| 192 | G_Temporal_Inf-3-R | NS |  |  |  |  |
| 193 | G_Temporal_Inf-4-L | NS |  |  |  |  |
| 194 | G_Temporal_Inf-4-R | NS |  |  |  |  |
| 195 | G_Temporal_Inf-5-L | NS |  |  |  |  |
| 196 | G_Temporal_Inf-5-R | NS |  |  |  |  |
| 197 | G_Temporal_Pole_Sup-1-L | NS |  |  |  |  |
| 198 | G_Temporal_Pole_Sup-1-R | NS |  |  |  |  |
| 199 | G_Temporal_Pole_Sup-2-L | NS |  |  |  |  |
| 200 | G_Temporal_Pole_Sup-2-R | NS |  |  |  |  |
| 201 | G_Temporal_Pole_Mid-1-L | 0.035 | 729 | 28.91 | 39.76 | 0.11 |
| 202 | G_Temporal_Pole_Mid-1-R | NS |  |  |  |  |
| 203 | G_Temporal_Pole_Mid-2-L | NS |  |  |  |  |
| 204 | G_Temporal_Pole_Mid-2-R | 0.005 | 337.5 | 40.77 | 27.69 | -0.14 |
| 205 | G_Temporal_Pole_Mid-3-L | NS |  |  |  |  |
| 206 | G_Temporal_Pole_Mid-3-R | NS |  |  |  |  |
| 207 | G_Frontal_Sup_Medial-1-L | NS |  |  |  |  |
| 208 | G_Frontal_Sup_Medial-1-R | NS |  |  |  |  |
| 209 | G_Frontal_Sup_Medial-2-L | NS |  |  |  |  |
| 210 | G_Frontal_Sup_Medial-2-R | NS |  |  |  |  |
| 211 | G_Frontal_Sup_Medial-3-L | NS |  |  |  |  |
| 212 | G_Frontal_Sup_Medial-3-R | NS |  |  |  |  |
| 213 | S_Anterior_Rostral-1-L | NS |  |  |  |  |
| 214 | S_Anterior_Rostral-1-R | NS |  |  |  |  |
| 215 | G_Frontal_Med_Orb-1-L | NS |  |  |  |  |
| 216 | G_Frontal_Med_Orb-1-R | NS |  |  |  |  |
| 217 | G_Frontal_Med_Orb-2-L | NS |  |  |  |  |
| 218 | G_Frontal_Med_Orb-2-R | NS |  |  |  |  |
| 219 | G_subcallosal-1-L | NS |  |  |  |  |
| 220 | G_subcallosal-1-R | NS |  |  |  |  |
| 221 | G_Supp_Motor_Area-1-L | NS |  |  |  |  |
| 222 | G_Supp_Motor_Area-1-R | NS |  |  |  |  |
| 223 | G_Supp_Motor_Area-2-L | NS |  |  |  |  |
| 224 | G_Supp_Motor_Area-2-R | NS |  |  |  |  |
| 225 | G_Supp_Motor_Area-3-L | NS |  |  |  |  |
| 226 | G_Supp_Motor_Area-3-R | NS |  |  |  |  |
| 227 | S_Cingulate-1-L | NS |  |  |  |  |
| 228 | S_Cingulate-1-R | NS |  |  |  |  |
| 229 | S_Cingulate-2-L | NS |  |  |  |  |
| 230 | S_Cingulate-2-R | NS |  |  |  |  |
| 231 | S_Cingulate-3-L | NS |  |  |  |  |
| 232 | S_Cingulate-3-R | NS |  |  |  |  |
| 233 | S_Cingulate-4-L | NS |  |  |  |  |
| 234 | S_Cingulate-4-R | NS |  |  |  |  |
| 235 | S_Cingulate-5-L | NS |  |  |  |  |
| 236 | S_Cingulate-5-R | 0.034 | 392 | 39.12 | 29.56 | -0.11 |
| 237 | S_Cingulate-6-L | NS |  |  |  |  |
| 238 | S_Cingulate-6-R | NS |  |  |  |  |
| 239 | S_Cingulate-7-L | NS |  |  |  |  |
| 240 | S_Cingulate-7-R | NS |  |  |  |  |
| 241 | G_Cingulum_Ant-1-L | NS |  |  |  |  |
| 242 | G_Cingulum_Ant-1-R | NS |  |  |  |  |
| 243 | G_Cingulum_Ant-2-L | NS |  |  |  |  |
| 244 | G_Cingulum_Ant-2-R | NS |  |  |  |  |
| 245 | G_Cingulum_Mid-1-L | 0.047 | 403 | 38.79 | 29.47 | 0.10 |
| 246 | G_Cingulum_Mid-1-R | NS |  |  |  |  |
| 247 | G_Cingulum_Mid-2-L | NS |  |  |  |  |
| 248 | G_Cingulum_Mid-2-R | NS |  |  |  |  |
| 249 | G_Cingulum_Mid-3-L | NS |  |  |  |  |
| 250 | G_Cingulum_Mid-3-R | NS |  |  |  |  |
| 251 | G_Cingulum_Post-1-L | 0.009 | 354 | 40.27 | 28.29 | -0.13 |
| 252 | G_Cingulum_Post-1-R | NS |  |  |  |  |
| 253 | G_Cingulum_Post-2-L | NS |  |  |  |  |
| 254 | G_Cingulum_Post-2-R | NS |  |  |  |  |
| 255 | G_Cingulum_Post-3-L | NS |  |  |  |  |
| 256 | G_Cingulum_Post-3-R | NS |  |  |  |  |
| 257 | G_Paracentral_Lobule-1-L | NS |  |  |  |  |
| 258 | G_Paracentral_Lobule-1-R | NS |  |  |  |  |
| 259 | G_Paracentral_Lobule-2-L | NS |  |  |  |  |
| 260 | G_Paracentral_Lobule-2-R | NS |  |  |  |  |
| 261 | G_Paracentral_Lobule-3-L | NS |  |  |  |  |
| 262 | G_Paracentral_Lobule-3-R | NS |  |  |  |  |
| 263 | G_Paracentral_Lobule-4-L | NS |  |  |  |  |
| 264 | G_Paracentral_Lobule-4-R | NS |  |  |  |  |
| 265 | G_Precuneus-1-L | NS |  |  |  |  |
| 266 | G_Precuneus-1-R | NS |  |  |  |  |
| 267 | G_Precuneus-2-L | 0.03 | 734 | 28.76 | 39.35 | 0.11 |
| 268 | G_Precuneus-2-R | NS |  |  |  |  |
| 269 | G_Precuneus-3-L | NS |  |  |  |  |
| 270 | G_Precuneus-3-R | NS |  |  |  |  |
| 271 | G_Precuneus-4-L | NS |  |  |  |  |
| 272 | G_Precuneus-4-R | NS |  |  |  |  |
| 273 | G_Precuneus-5-L | NS |  |  |  |  |
| 274 | G_Precuneus-5-R | NS |  |  |  |  |
| 275 | G_Precuneus-6-L | NS |  |  |  |  |
| 276 | G_Precuneus-6-R | NS |  |  |  |  |
| 277 | G_Precuneus-7-L | NS |  |  |  |  |
| 278 | G_Precuneus-7-R | NS |  |  |  |  |
| 279 | G_Precuneus-8-L | NS |  |  |  |  |
| 280 | G_Precuneus-8-R | NS |  |  |  |  |
| 281 | G_Precuneus-9-L | NS |  |  |  |  |
| 282 | G_Precuneus-9-R | NS |  |  |  |  |
| 283 | S_Parietooccipital-1-L | NS |  |  |  |  |
| 284 | S_Parietooccipital-1-R | NS |  |  |  |  |
| 285 | S_Parietooccipital-2-L | 0.036 | 632 | 31.85 | 36.09 | -0.11 |
| 286 | S_Parietooccipital-2-R | NS |  |  |  |  |
| 287 | S_Parietooccipital-3-L | NS |  |  |  |  |
| 288 | S_Parietooccipital-3-R | NS |  |  |  |  |
| 289 | S_Parietooccipital-4-L | NS |  |  |  |  |
| 290 | S_Parietooccipital-4-R | NS |  |  |  |  |
| 291 | S_Parietooccipital-5-L | NS |  |  |  |  |
| 292 | S_Parietooccipital-5-R | NS |  |  |  |  |
| 293 | S_Parietooccipital-6-L | NS |  |  |  |  |
| 294 | S_Parietooccipital-6-R | NS |  |  |  |  |
| 295 | G_Cuneus-1-L | NS |  |  |  |  |
| 296 | G_Cuneus-1-R | NS |  |  |  |  |
| 297 | G_Cuneus-2-L | NS |  |  |  |  |
| 298 | G_Cuneus-2-R | NS |  |  |  |  |
| 299 | G_Calcarine-1-L | 0.037 | 727.5 | 28.95 | 38.9 | 0.11 |
| 300 | G_Calcarine-1-R | NS |  |  |  |  |
| 301 | G_Calcarine-2-L | NS |  |  |  |  |
| 302 | G_Calcarine-2-R | NS |  |  |  |  |
| 303 | G_Calcarine-3-L | NS |  |  |  |  |
| 304 | G_Calcarine-3-R | NS |  |  |  |  |
| 305 | G_Lingual-1-L | 0.036 | 394 | 39.06 | 39.06 | -0.11 |
| 306 | G_Lingual-1-R | NS |  |  |  |  |
| 307 | G_Lingual-2-L | NS |  |  |  |  |
| 308 | G_Lingual-2-R | NS |  |  |  |  |
| 309 | G_Lingual-3-L | NS |  |  |  |  |
| 310 | G_Lingual-3-R | NS |  |  |  |  |
| 311 | G_Lingual-4-L | NS |  |  |  |  |
| 312 | G_Lingual-4-R | NS |  |  |  |  |
| 313 | G_Lingual-5-L | NS |  |  |  |  |
| 314 | G_Lingual-5-R | NS |  |  |  |  |
| 315 | G_Lingual-6-L | NS |  |  |  |  |
| 316 | G_Lingual-6-R | NS |  |  |  |  |
| 317 | G_Hippocampus-1-L | 0.024 | 381.5 | 39.44 | 29.51 | -0.11 |
| 318 | G_Hippocampus-1-R | NS |  |  |  |  |
| 319 | G_Hippocampus-2-L | NS |  |  |  |  |
| 320 | G_Hippocampus-2-R | 0.032 | 390 | 39.18 | 29.03 | -0.11 |
| 321 | G_ParaHippocampal-1-L | NS |  |  |  |  |
| 322 | G_ParaHippocampal-1-R | NS |  |  |  |  |
| 323 | G_ParaHippocampal-2-L | NS |  |  |  |  |
| 324 | G_ParaHippocampal-2-R | NS |  |  |  |  |
| 325 | G_ParaHippocampal-3-L | NS |  |  |  |  |
| 326 | G_ParaHippocampal-3-R | NS |  |  |  |  |
| 327 | G_ParaHippocampal-4-L | NS |  |  |  |  |
| 328 | G_ParaHippocampal-4-R | NS |  |  |  |  |
| 329 | G_ParaHippocampal-5-L | NS |  |  |  |  |
| 330 | G_ParaHippocampal-5-R | NS |  |  |  |  |
| 331 | G_Fusiform-1-L | NS |  |  |  |  |
| 332 | G_Fusiform-1-R | NS |  |  |  |  |
| 333 | G_Fusiform-2-L | NS |  |  |  |  |
| 334 | G_Fusiform-2-R | NS |  |  |  |  |
| 335 | G_Fusiform-3-L | NS |  |  |  |  |
| 336 | G_Fusiform-3-R | NS |  |  |  |  |
| 337 | G_Fusiform-4-L | NS |  |  |  |  |
| 338 | G_Fusiform-4-R | NS |  |  |  |  |
| 339 | G_Fusiform-5-L | NS |  |  |  |  |
| 340 | G_Fusiform-5-R | 0.034 | 392 | 39.12 | 29.21 | -0.11 |
| 341 | G_Fusiform-6-L | NS |  |  |  |  |
| 342 | G_Fusiform-6-R | 0.04 | 397 | 38.97 | 29.79 | -0.10 |
| 343 | G_Fusiform-7-L | NS |  |  |  |  |
| 344 | G_Fusiform-7-R | 0.008 | 348 | 40.75 | 28.53 | -0.14 |
| 345 | N_Amygdala-1-L | NS |  |  |  |  |
| 346 | N_Amygdala-1-R | NS |  |  |  |  |
| 347 | N_Caudate-1-L | NS |  |  |  |  |
| 348 | N_Caudate-1-R | NS |  |  |  |  |
| 349 | N_Caudate-2-L | NS |  |  |  |  |
| 350 | N_Caudate-2-R | NS |  |  |  |  |
| 351 | N_Caudate-3-L | NS |  |  |  |  |
| 352 | N_Caudate-3-R | NS |  |  |  |  |
| 353 | N_Caudate-4-L | NS |  |  |  |  |
| 354 | N_Caudate-4-R | NS |  |  |  |  |
| 355 | N_Caudate-5-L | NS |  |  |  |  |
| 356 | N_Caudate-5-R | NS |  |  |  |  |
| 357 | N_Caudate-6-L | NS |  |  |  |  |
| 358 | N_Caudate-6-R | NS |  |  |  |  |
| 359 | N_Caudate-7-L | NS |  |  |  |  |
| 360 | N_Caudate-7-R | NS |  |  |  |  |
| 361 | N_Pallidum-1-L | NS |  |  |  |  |
| 362 | N_Pallidum-1-R | NS |  |  |  |  |
| 363 | N_Putamen-2-L | NS |  |  |  |  |
| 364 | N_Putamen-2-R | NS |  |  |  |  |
| 365 | N_Putamen-3-L | NS |  |  |  |  |
| 366 | N_Putamen-3-R | NS |  |  |  |  |
| 367 | N_Thalamus-1-L | NS |  |  |  |  |
| 368 | N_Thalamus-1-R | NS |  |  |  |  |
| 369 | N_Thalamus-2-L | NS |  |  |  |  |
| 370 | N_Thalamus-2-R | NS |  |  |  |  |
| 371 | N_Thalamus-3-L | NS |  |  |  |  |
| 372 | N_Thalamus-3-R | NS |  |  |  |  |
| 373 | N_Thalamus-4-L | NS |  |  |  |  |
| 374 | N_Thalamus-4-R | NS |  |  |  |  |
| 375 | N_Thalamus-5-L | NS |  |  |  |  |
| 376 | N_Thalamus-5-R | NS |  |  |  |  |
| 377 | N_Thalamus-6-L | NS |  |  |  |  |
| 378 | N_Thalamus-6-R | NS |  |  |  |  |
| 379 | N_Thalamus-7-L | NS |  |  |  |  |
| 380 | N_Thalamus-7-R | NS |  |  |  |  |
| 381 | N_Thalamus-8-L | NS |  |  |  |  |
| 382 | N_Thalamus-8-R | NS |  |  |  |  |
| 383 | N_Thalamus-9- L | NS |  |  |  |  |
| 384 | N_Thalamus-9-R | NS |  |  |  |  |

NS = not significant at p < 0.05. Effect size calculated as r = $Z/\surd N$. All results displayed to two decimal points.

Supplementary Table 6. Frequency of hub nodes in each region

| **AICHA region** | **AICHA region name** | **Controls** | **IGE (both groups)** | **WC-IGE** | **DR-IGE** |
| --- | --- | --- | --- | --- | --- |
| 1 | G_Frontal_Sup-1-L | 3 | 1 | 0 | 1 |
| 2 | G_Frontal_Sup-1-R | 1 | 0 | 0 | 0 |
| 3 | G_Frontal_Sup-2-L | 12 | 11 | 5 | 6 |
| 4 | G_Frontal_Sup-2-R | 14 | 20 | 3 | 17 |
| 5 | G_Frontal_Sup-3-L | 1 | 3 | 1 | 2 |
| 6 | G_Frontal_Sup-3-R | 0 | 1 | 0 | 1 |
| 7 | S_Sup_Frontal-1-L | 0 | 0 | 0 | 0 |
| 8 | S_Sup_Frontal-1-R | 1 | 4 | 1 | 3 |
| 9 | S_Sup_Frontal-2-L | 0 | 2 | 1 | 1 |
| 10 | S_Sup_Frontal-2-R | 3 | 2 | 1 | 1 |
| 11 | S_Sup_Frontal-3-L | 3 | 1 | 0 | 1 |
| 12 | S_Sup_Frontal-3-R | 2 | 5 | 1 | 4 |
| 13 | S_Sup_Frontal-4-L | 8 | 11 | 6 | 5 |
| 14 | S_Sup_Frontal-4-R | 9 | 8 | 3 | 5 |
| 15 | S_Sup_Frontal-5-L | 5 | 3 | 0 | 3 |
| 16 | S_Sup_Frontal-5-R | 4 | 5 | 1 | 4 |
| 17 | S_Sup_Frontal-6-L | 7 | 3 | 0 | 3 |
| 18 | S_Sup_Frontal-6-R | 4 | 12 | 4 | 8 |
| 19 | G_Frontal_Mid-1-L | 4 | 4 | 1 | 3 |
| 20 | G_Frontal_Mid-1-R | 5 | 6 | 1 | 5 |
| 21 | G_Frontal_Mid-2-L | 2 | 3 | 0 | 3 |
| 22 | G_Frontal_Mid-2-R | 8 | 8 | 3 | 5 |
| 23 | G_Frontal_Mid-3-L | 0 | 2 | 2 | 0 |
| 24 | G_Frontal_Mid-3-R | 4 | 6 | 0 | 6 |
| 25 | G_Frontal_Mid-4-L | 4 | 8 | 2 | 6 |
| 26 | G_Frontal_Mid-4-R | 1 | 4 | 1 | 3 |
| 27 | G_Frontal_Mid-5-L | 2 | 4 | 2 | 2 |
| 28 | G_Frontal_Mid-5-R | 4 | 4 | 0 | 4 |
| 29 | S_Inf_Frontal-1-L | 3 | 4 | 1 | 3 |
| 30 | S_Inf_Frontal-1-R | 2 | 2 | 1 | 1 |
| 31 | S_Inf_Frontal-2-L | 5 | 2 | 0 | 2 |
| 32 | S_Inf_Frontal-2-R | 4 | 4 | 0 | 4 |
| 33 | G_Frontal_Inf_Tri-1-L | 3 | 3 | 0 | 3 |
| 34 | G_Frontal_Inf_Tri-1-R | 2 | 3 | 2 | 1 |
| 35 | G_Frontal_Sup_Orb-1-L | 1 | 1 | 1 | 0 |
| 36 | G_Frontal_Sup_Orb-1-R | 0 | 0 | 0 | 0 |
| 37 | G_Frontal_Mid_Orb-1-L | 0 | 1 | 0 | 1 |
| 38 | G_Frontal_Mid_Orb-1-R | 0 | 1 | 0 | 1 |
| 39 | G_Frontal_Mid_Orb-2-L | 4 | 3 | 0 | 3 |
| 40 | G_Frontal_Mid_Orb-2-R | 2 | 4 | 2 | 2 |
| 41 | G_Frontal_Inf_Orb-1-L | 0 | 0 | 0 | 0 |
| 42 | G_Frontal_Inf_Orb-1-R 4 | 4 | 1 | 0 | 1 |
| 43 | G_Frontal_Inf_Orb-2-L | 0 | 0 | 0 | 0 |
| 44 | G_Frontal_Inf_Orb-2-R | 1 | 0 | 0 | 0 |
| 45 | S_Orbital-1-L | 1 | 0 | 0 | 1 |
| 46 | S_Orbital-1-R | 1 | 1 | 0 | 0 |
| 47 | S_Orbital-2-L | 0 | 0 | 0 | 0 |
| 48 | S_Orbital-2-R | 1 | 0 | 0 | 4 |
| 49 | S_Olfactory-1-L | 13 | 4 | 0 | 0 |
| 50 | S_Olfactory-1-R | 0 | 0 | 1 | 2 |
| 51 | S_Precentral-1-L | 3 | 3 | 3 | 6 |
| 52 | S_Precentral-1-R | 4 | 9 | 0 | 0 |
| 53 | S_Precentral-2-L | 0 | 0 | 0 | 2 |
| 54 | S_Precentral-2-R | 0 | 2 | 1 | 0 |
| 55 | S_Precentral-3-L | 0 | 1 | 1 | 0 |
| 56 | S_Precentral-3-R | 2 | 1 | 0 | 2 |
| 57 | S_Precentral-4-L | 2 | 2 | 1 | 5 |
| 58 | S_Precentral-4-R | 7 | 6 | 0 | 1 |
| 59 | S_Precentral-5-L | 2 | 1 | 1 | 3 |
| 60 | S_Precentral-5-R | 1 | 4 | 0 | 1 |
| 61 | S_Precentral-6-L | 0 | 1 | 1 | 1 |
| 62 | S_Precentral-6-R | 0 | 2 | 0 | 0 |
| 63 | S_Rolando-1-L | 1 | 0 | 1 | 3 |
| 64 | S_Rolando-1-R | 4 | 4 | 1 | 2 |
| 65 | S_Rolando-2-L | 2 | 3 | 0 | 2 |
| 66 | S_Rolando-2-R | 2 | 2 | 1 | 2 |
| 67 | S_Rolando-3-L | 3 | 3 | 1 | 4 |
| 68 | S_Rolando-3-R | 4 | 5 | 1 | 7 |
| 69 | S_Rolando-4-L | 7 | 8 | 1 | 3 |
| 70 | S_Rolando-4-R | 7 | 4 | 1 | 1 |
| 71 | S_Postcentral-1-L | 2 | 2 | 0 | 2 |
| 72 | S_Postcentral-1-R | 2 | 2 | 0 | 6 |
| 73 | S_Postcentral-2-L | 13 | 6 | 2 | 6 |
| 74 | S_Postcentral-2-R | 5 | 8 | 0 | 4 |
| 75 | S_Postcentral-3-L | 2 | 4 | 0 | 3 |
| 76 | S_Postcentral-3-R | 6 | 3 | 1 | 0 |
| 77 | G_Parietal_Sup-1-L | 2 | 1 | 1 | 1 |
| 78 | G_Parietal_Sup-1-R | 1 | 2 | 1 | 3 |
| 79 | G_Parietal_Sup-2-L | 7 | 4 | 4 | 3 |
| 80 | G_Parietal_Sup-2-R | 7 | 7 | 1 | 5 |
| 81 | G_Parietal_Sup-3-L | 8 | 6 | 0 | 5 |
| 82 | G_Parietal_Sup-3-R | 6 | 5 | 1 | 3 |
| 83 | G_Parietal_Sup-4-L | 7 | 4 | 1 | 3 |
| 84 | G_Parietal_Sup-4-R | 4 | 4 | 1 | 6 |
| 85 | G_Parietal_Sup-5-L | 9 | 7 | 1 | 4 |
| 86 | G_Parietal_Sup-5-R | 10 | 5 | 3 | 4 |
| 87 | G_Supramarginal-1-L | 11 | 7 | 2 | 6 |
| 88 | G_Supramarginal-1-R | 7 | 8 | 1 | 6 |
| 89 | G_SupraMarginal-2-L | 6 | 7 | 1 | 8 |
| 90 | G_SupraMarginal-2-R | 10 | 9 | 4 | 5 |
| 91 | G_Supramarginal-3-L | 8 | 9 | 2 | 5 |
| 92 | G_Supramarginal-3-R | 8 | 7 | 1 | 5 |
| 93 | G_Supramarginal-4-L | 9 | 6 | 2 | 8 |
| 94 | G_Supramarginal-4-R | 9 | 10 | 0 | 1 |
| 95 | G_SupraMarginal-5-L | 1 | 1 | 1 | 3 |
| 96 | G_SupraMarginal-5-R | 3 | 4 | 1 | 4 |
| 97 | G_SupraMarginal-6-L | 1 | 5 | 1 | 3 |
| 98 | G_SupraMarginal-6-R | 10 | 4 | 1 | 4 |
| 99 | G_SupraMarginal-7-L | 4 | 5 | 0 | 1 |
| 100 | G_SupraMarginal-7-R | 6 | 1 | 3 | 9 |
| 101 | G_Angular-1-L | 18 | 12 | 3 | 6 |
| 102 | G_Angular-1-R | 14 | 9 | 6 | 13 |
| 103 | G_Angular-2-L | 23 | 19 | 6 | 13 |
| 104 | G_Angular-2-R | 17 | 19 | 5 | 7 |
| 105 | G_Angular-3-L | 14 | 12 | 3 | 7 |
| 106 | G_Angular-3-R | 9 | 10 | 1 | 4 |
| 107 | G_Parietal_Inf-1-L | 8 | 5 | 1 | 7 |
| 108 | G_Parietal_Inf-1-R | 9 | 8 | 1 | 8 |
| 109 | S_Intraparietal-1-L | 12 | 9 | 3 | 10 |
| 110 | S_Intraparietal-1-R | 14 | 13 | 4 | 7 |
| 111 | S_Intraparietal-2-L | 10 | 11 | 1 | 9 |
| 112 | S_Intraparietal-2-R | 6 | 10 | 3 | 10 |
| 113 | S_Intraparietal-3-L | 8 | 13 | 3 | 4 |
| 114 | S_Intraparietal-3-R | 8 | 7 | 2 | 1 |
| 115 | S_Intraoccipital-1-L | 0 | 3 | 1 | 0 |
| 116 | S_Intraoccipital-1-R | 3 | 1 | 3 | 1 |
| 117 | G_Occipital_Pole-1-L | 1 | 4 | 1 | 2 |
| 118 | G_Occipital_Pole-1-R | 2 | 3 | 2 | 0 |
| 119 | G_Occipital_Lat-1-L | 1 | 2 | 0 | 1 |
| 120 | G_Occipital_Lat-1-R | 0 | 1 | 1 | 2 |
| 121 | G_Occipital_Lat-2-L | 3 | 3 | 2 | 4 |
| 122 | G_Occipital_Lat-2-R | 1 | 6 | 0 | 0 |
| 123 | G_Occipital_Lat-3-L | 1 | 0 | 0 | 1 |
| 124 | G_Occipital_Lat-3-R | 2 | 1 | 4 | 1 |
| 125 | G_Occipital_Lat-4-L | 6 | 5 | 1 | 1 |
| 126 | G_Occipital_Lat-4-R | 3 | 2 | 6 | 8 |
| 127 | G_Occipital_Lat-5-L | 13 | 14 | 5 | 8 |
| 128 | G_Occipital_Lat-5-R | 8 | 13 | 1 | 2 |
| 129 | G_Occipital_Sup-1-L | 9 | 3 | 1 | 0 |
| 130 | G_Occipital_Sup-1-R | 3 | 1 | 1 | 4 |
| 131 | G_Occipital_Sup-2-L | 10 | 5 | 2 | 6 |
| 132 | G_Occipital_Sup-2-R | 6 | 8 | 3 | 5 |
| 133 | G_Occipital_Mid-1-L | 11 | 8 | 3 | 7 |
| 134 | G_Occipital_Mid-1-R | 7 | 10 | 1 | 1 |
| 135 | G_Occipital_Mid-2-L | 5 | 2 | 1 | 2 |
| 136 | G_Occipital_Mid-2-R | 3 | 3 | 1 | 4 |
| 137 | G_Occipital_Mid-3-L | 3 | 5 | 4 | 4 |
| 138 | G_Occipital_Mid-3-R | 2 | 8 | 3 | 5 |
| 139 | G_Occipital_Mid-4-L | 6 | 8 | 3 | 5 |
| 140 | G_Occipital_Mid-4-R | 4 | 8 | 0 | 1 |
| 141 | G_Occipital_Inf-1-L | 1 | 1 | 2 | 2 |
| 142 | G_Occipital_Inf-1-R | 2 | 4 | 0 | 3 |
| 143 | G_Occipital_Inf-2-L | 6 | 3 | 2 | 4 |
| 144 | G_Occipital_Inf-2-R | 3 | 6 | 0 | 2 |
| 145 | G_Insula-anterior-1-L | 0 | 2 | 0 | 0 |
| 146 | G_Insula-anterior-1-R | 1 | 0 | 0 | 0 |
| 147 | G_Insula-anterior-2-L | 1 | 0 | 0 | 2 |
| 148 | G_Insula-anterior-2-R | 1 | 2 | 1 | 0 |
| 149 | G_Insula-anterior-3-L | 1 | 1 | 2 | 4 |
| 150 | G_Insula-anterior-3-R | 4 | 6 | 3 | 3 |
| 151 | G_Insula-anterior-4-L | 5 | 6 | 4 | 11 |
| 152 | G_Insula-anterior-4-R | 9 | 15 | 1 | 0 |
| 153 | G_Insula-anterior-5-L | 6 | 1 | 2 | 4 |
| 154 | G_Insula-anterior-5-R | 10 | 6 | 1 | 2 |
| 155 | G_Insula-posterior-1-L | 1 | 3 | 0 | 0 |
| 156 | G_Insula-posterior-1-R | 2 | 0 | 6 | 4 |
| 157 | G_Rolandic_Oper-1-L | 7 | 10 | 3 | 7 |
| 158 | G_Rolandic_Oper-1-R | 4 | 10 | 3 | 3 |
| 159 | G_Rolandic_Oper-2-L | 3 | 6 | 1 | 2 |
| 160 | G_Rolandic_Oper-2-R | 2 | 3 | 1 | 2 |
| 161 | G_Temporal_Sup-1-L | 6 | 3 | 1 | 5 |
| 162 | G_Temporal_Sup-1-R | 2 | 6 | 0 | 1 |
| 163 | G_Temporal_Sup-2-L | 0 | 1 | 1 | 0 |
| 164 | G_Temporal_Sup-2-R | 1 | 1 | 1 | 3 |
| 165 | G_Temporal_Sup-3-L | 8 | 4 | 4 | 4 |
| 166 | G_Temporal_Sup-3-R | 7 | 8 | 0 | 3 |
| 167 | G_Temporal_Sup-4-L | 4 | 3 | 1 | 0 |
| 168 | G_Temporal_Sup-4-R | 2 | 1 | 0 | 0 |
| 169 | S_Sup_Temporal-1-L | 3 | 0 | 0 | 1 |
| 170 | S_Sup_Temporal-1-R | 1 | 1 | 0 | 1 |
| 171 | S_Sup_Temporal-2-L | 1 | 1 | 2 | 6 |
| 172 | S_Sup_Temporal-2-R | 5 | 8 | 1 | 2 |
| 173 | S_Sup_Temporal-3-L | 0 | 3 | 2 | 1 |
| 174 | S_Sup_Temporal-3-R | 1 | 3 | 1 | 1 |
| 175 | S_Sup_Temporal-4-L | 1 | 2 | 1 | 2 |
| 176 | S_Sup_Temporal-4-R | 3 | 3 | 6 | 11 |
| 177 | S_Sup_Temporal-5-L | 17 | 17 | 4 | 11 |
| 178 | S_Sup_Temporal-5-R | 14 | 15 | 3 | 3 |
| 179 | G_Temporal_Mid-1-L | 5 | 6 | 1 | 6 |
| 180 | G_Temporal_Mid-1-R | 3 | 7 | 2 | 2 |
| 181 | G_Temporal_Mid-2-L | 11 | 4 | 4 | 6 |
| 182 | G_Temporal_Mid-2-R | 5 | 10 | 2 | 2 |
| 183 | G_Temporal_Mid-3-L | 3 | 4 | 2 | 1 |
| 184 | G_Temporal_Mid-3-R | 0 | 3 | 3 | 1 |
| 185 | G_Temporal_Mid-4-L | 4 | 4 | 0 | 2 |
| 186 | G_Temporal_Mid-4-R | 6 | 2 | 0 | 0 |
| 187 | G_Temporal_Inf-1-L | 1 | 0 | 0 | 1 |
| 188 | G_Temporal_Inf-1-R | 0 | 1 | 0 | 0 |
| 189 | G_Temporal_Inf-2-L | 1 | 0 | 0 | 1 |
| 190 | G_Temporal_Inf-2-R | 4 | 1 | 1 | 2 |
| 191 | G_Temporal_Inf-3-L | 5 | 3 | 0 | 2 |
| 192 | G_Temporal_Inf-3-R | 1 | 2 | 2 | 3 |
| 193 | G_Temporal_Inf-4-L | 7 | 5 | 0 | 4 |
| 194 | G_Temporal_Inf-4-R | 6 | 4 | 0 | 0 |
| 195 | G_Temporal_Inf-5-L | 0 | 0 | 3 | 1 |
| 196 | G_Temporal_Inf-5-R | 4 | 4 | 1 | 1 |
| 197 | G_Temporal_Pole_Sup-1-L | 0 | 2 | 1 | 0 |
| 198 | G_Temporal_Pole_Sup-1-R | 1 | 1 | 1 | 0 |
| 199 | G_Temporal_Pole_Sup-2-L | 0 | 1 | 1 | 0 |
| 200 | G_Temporal_Pole_Sup-2-R | 0 | 1 | 1 | 3 |
| 201 | G_Temporal_Pole_Mid-1-L | 7 | 4 | 2 | 6 |
| 202 | G_Temporal_Pole_Mid-1-R | 2 | 8 | 0 | 0 |
| 203 | G_Temporal_Pole_Mid-2-L | 0 | 0 | 0 | 0 |
| 204 | G_Temporal_Pole_Mid-2-R | 1 | 0 | 0 | 1 |
| 205 | G_Temporal_Pole_Mid-3-L | 0 | 1 | 0 | 0 |
| 206 | G_Temporal_Pole_Mid-3-R | 0 | 0 | 1 | 7 |
| 207 | G_Frontal_Sup_Medial-1-L | 6 | 8 | 2 | 4 |
| 208 | G_Frontal_Sup_Medial-1-R | 6 | 6 | 3 | 9 |
| 209 | G_Frontal_Sup_Medial-2-L | 6 | 12 | 3 | 3 |
| 210 | G_Frontal_Sup_Medial-2-R | 9 | 6 | 1 | 1 |
| 211 | G_Frontal_Sup_Medial-3-L | 0 | 2 | 1 | 1 |
| 212 | G_Frontal_Sup_Medial-3-R | 0 | 2 | 2 | 8 |
| 213 | S_Anterior_Rostral-1-L | 12 | 10 | 1 | 3 |
| 214 | S_Anterior_Rostral-1-R | 7 | 4 | 1 | 4 |
| 215 | G_Frontal_Med_Orb-1-L | 4 | 5 | 0 | 1 |
| 216 | G_Frontal_Med_Orb-1-R | 4 | 1 | 1 | 11 |
| 217 | G_Frontal_Med_Orb-2-L | 13 | 12 | 2 | 4 |
| 218 | G_Frontal_Med_Orb-2-R | 3 | 6 | 1 | 0 |
| 219 | G_subcallosal-1-L | 0 | 1 | 0 | 0 |
| 220 | G_subcallosal-1-R | 0 | 0 | 0 | 0 |
| 221 | G_Supp_Motor_Area-1-L | 0 | 0 | 0 | 1 |
| 222 | G_Supp_Motor_Area-1-R | 2 | 1 | 0 | 2 |
| 223 | G_Supp_Motor_Area-2-L | 2 | 2 | 0 | 1 |
| 224 | G_Supp_Motor_Area-2-R | 0 | 1 | 1 | 1 |
| 225 | G_Supp_Motor_Area-3-L | 0 | 2 | 0 | 3 |
| 226 | G_Supp_Motor_Area-3-R | 2 | 3 | 0 | 1 |
| 227 | S_Cingulate-1-L | 0 | 1 | 0 | 1 |
| 228 | S_Cingulate-1-R | 2 | 1 | 0 | 1 |
| 229 | S_Cingulate-2-L | 2 | 1 | 1 | 9 |
| 230 | S_Cingulate-2-R | 5 | 10 | 0 | 2 |
| 231 | S_Cingulate-3-L | 6 | 2 | 4 | 11 |
| 232 | S_Cingulate-3-R | 10 | 15 | 1 | 4 |
| 233 | S_Cingulate-4-L | 4 | 5 | 3 | 7 |
| 234 | S_Cingulate-4-R | 9 | 10 | 0 | 1 |
| 235 | S_Cingulate-5-L | 1 | 1 | 0 | 2 |
| 236 | S_Cingulate-5-R | 0 | 2 | 1 | 2 |
| 237 | S_Cingulate-6-L | 2 | 3 | 2 | 3 |
| 238 | S_Cingulate-6-R | 6 | 5 | 1 | 1 |
| 239 | S_Cingulate-7-L | 2 | 2 | 2 | 3 |
| 240 | S_Cingulate-7-R | 3 | 5 | 0 | 2 |
| 241 | G_Cingulum_Ant-1-L | 2 | 2 | 1 | 6 |
| 242 | G_Cingulum_Ant-1-R | 6 | 7 | 0 | 0 |
| 243 | G_Cingulum_Ant-2-L | 0 | 0 | 3 | 0 |
| 244 | G_Cingulum_Ant-2-R | 1 | 3 | 0 | 0 |
| 245 | G_Cingulum_Mid-1-L | 1 | 0 | 0 | 0 |
| 246 | G_Cingulum_Mid-1-R | 1 | 0 | 0 | 0 |
| 247 | G_Cingulum_Mid-2-L | 0 | 0 | 0 | 0 |
| 248 | G_Cingulum_Mid-2-R | 0 | 0 | 1 | 1 |
| 249 | G_Cingulum_Mid-3-L | 2 | 2 | 1 | 2 |
| 250 | G_Cingulum_Mid-3-R | 2 | 3 | 1 | 0 |
| 251 | G_Cingulum_Post-1-L | 0 | 1 | 1 | 1 |
| 252 | G_Cingulum_Post-1-R | 0 | 2 | 1 | 4 |
| 253 | G_Cingulum_Post-2-L | 1 | 5 | 4 | 5 |
| 254 | G_Cingulum_Post-2-R | 4 | 9 | 1 | 3 |
| 255 | G_Cingulum_Post-3-L | 0 | 4 | 0 | 4 |
| 256 | G_Cingulum_Post-3-R | 1 | 4 | 0 | 1 |
| 257 | G_Paracentral_Lobule-1-L | 0 | 1 | 0 | 3 |
| 258 | G_Paracentral_Lobule-1-R | 1 | 3 | 0 | 1 |
| 259 | G_Paracentral_Lobule-2-L | 0 | 1 | 0 | 2 |
| 260 | G_Paracentral_Lobule-2-R | 2 | 2 | 1 | 1 |
| 261 | G_Paracentral_Lobule-3-L | 0 | 2 | 1 | 2 |
| 262 | G_Paracentral_Lobule-3-R | 1 | 3 | 1 | 0 |
| 263 | G_Paracentral_Lobule-4-L | 0 | 1 | 0 | 1 |
| 264 | G_Paracentral_Lobule-4-R | 0 | 1 | 2 | 12 |
| 265 | G_Precuneus-1-L | 9 | 14 | 4 | 5 |
| 266 | G_Precuneus-1-R | 6 | 9 | 3 | 8 |
| 267 | G_Precuneus-2-L | 14 | 11 | 4 | 7 |
| 268 | G_Precuneus-2-R | 12 | 12 | 5 | 14 |
| 269 | G_Precuneus-3-L | 17 | 19 | 6 | 10 |
| 270 | G_Precuneus-3-R | 24 | 16 | 1 | 2 |
| 271 | G_Precuneus-4-L | 2 | 3 | 1 | 7 |
| 272 | G_Precuneus-4-R | 6 | 8 | 0 | 1 |
| 273 | G_Precuneus-5-L | 4 | 1 | 1 | 2 |
| 274 | G_Precuneus-5-R | 6 | 3 | 1 | 6 |
| 275 | G_Precuneus-6-L | 6 | 7 | 1 | 6 |
| 276 | G_Precuneus-6-R | 9 | 7 | 1 | 6 |
| 277 | G_Precuneus-7-L | 9 | 7 | 2 | 6 |
| 278 | G_Precuneus-7-R | 12 | 8 | 0 | 3 |
| 279 | G_Precuneus-8-L | 4 | 3 | 3 | 9 |
| 280 | G_Precuneus-8-R | 10 | 12 | 1 | 4 |
| 281 | G_Precuneus-9-L | 5 | 5 | 4 | 3 |
| 282 | G_Precuneus-9-R | 7 | 7 | 2 | 7 |
| 283 | S_Parietooccipital-1-L | 5 | 9 | 4 | 7 |
| 284 | S_Parietooccipital-1-R | 5 | 12 | 1 | 1 |
| 285 | S_Parietooccipital-2-L | 1 | 2 | 1 | 2 |
| 286 | S_Parietooccipital-2-R | 3 | 3 | 0 | 3 |
| 287 | S_Parietooccipital-3-L | 7 | 3 | 1 | 5 |
| 288 | S_Parietooccipital-3-R | 6 | 6 | 1 | 2 |
| 289 | S_Parietooccipital-4-L | 3 | 3 | 1 | 1 |
| 290 | S_Parietooccipital-4-R | 3 | 2 | 0 | 1 |
| 291 | S_Parietooccipital-5-L | 1 | 1 | 0 | 2 |
| 292 | S_Parietooccipital-5-R | 0 | 2 | 0 | 4 |
| 293 | S_Parietooccipital-6-L | 4 | 4 | 0 | 1 |
| 294 | S_Parietooccipital-6-R | 2 | 1 | 0 | 3 |
| 295 | G_Cuneus-1-L | 6 | 3 | 1 | 1 |
| 296 | G_Cuneus-1-R | 1 | 2 | 1 | 2 |
| 297 | G_Cuneus-2-L | 0 | 3 | 1 | 1 |
| 298 | G_Cuneus-2-R | 1 | 2 | 1 | 3 |
| 299 | G_Calcarine-1-L | 3 | 4 | 1 | 3 |
| 300 | G_Calcarine-1-R | 1 | 4 | 0 | 5 |
| 301 | G_Calcarine-2-L | 6 | 5 | 2 | 4 |
| 302 | G_Calcarine-2-R | 5 | 6 | 0 | 4 |
| 303 | G_Calcarine-3-L | 1 | 4 | 2 | 3 |
| 304 | G_Calcarine-3-R | 2 | 5 | 0 | 1 |
| 305 | G_Lingual-1-L | 0 | 1 | 0 | 1 |
| 306 | G_Lingual-1-R | 1 | 1 | 1 | 1 |
| 307 | G_Lingual-2-L | 3 | 2 | 0 | 1 |
| 308 | G_Lingual-2-R | 1 | 1 | 2 | 1 |
| 309 | G_Lingual-3-L | 1 | 3 | 1 | 1 |
| 310 | G_Lingual-3-R | 3 | 2 | 0 | 1 |
| 311 | G_Lingual-4-L | 2 | 1 | 0 | 0 |
| 312 | G_Lingual-4-R | 1 | 0 | 0 | 0 |
| 313 | G_Lingual-5-L | 1 | 0 | 0 | 1 |
| 314 | G_Lingual-5-R | 1 | 1 | 0 | 1 |
| 315 | G_Lingual-6-L | 0 | 1 | 0 | 2 |
| 316 | G_Lingual-6-R | 0 | 2 | 0 | 0 |
| 317 | G_Hippocampus-1-L | 0 | 0 | 0 | 2 |
| 318 | G_Hippocampus-1-R | 0 | 2 | 0 | 0 |
| 319 | G_Hippocampus-2-L | 1 | 0 | 0 | 0 |
| 320 | G_Hippocampus-2-R | 1 | 0 | 0 | 1 |
| 321 | G_ParaHippocampal-1-L | 1 | 1 | 0 | 0 |
| 322 | G_ParaHippocampal-1-R | 0 | 0 | 0 | 1 |
| 323 | G_ParaHippocampal-2-L | 1 | 1 | 0 | 1 |
| 324 | G_ParaHippocampal-2-R | 1 | 1 | 0 | 1 |
| 325 | G_ParaHippocampal-3-L | 0 | 1 | 0 | 1 |
| 326 | G_ParaHippocampal-3-R | 0 | 1 | 0 | 1 |
| 327 | G_ParaHippocampal-4-L | 0 | 1 | 1 | 0 |
| 328 | G_ParaHippocampal-4-R | 4 | 1 | 0 | 0 |
| 329 | G_ParaHippocampal-5-L | 3 | 0 | 0 | 2 |
| 330 | G_ParaHippocampal-5-R | 1 | 2 | 1 | 1 |
| 331 | G_Fusiform-1-L | 0 | 2 | 0 | 1 |
| 332 | G_Fusiform-1-R | 0 | 1 | 0 | 1 |
| 333 | G_Fusiform-2-L | 0 | 1 | 0 | 0 |
| 334 | G_Fusiform-2-R | 0 | 0 | 0 | 0 |
| 335 | G_Fusiform-3-L | 0 | 0 | 1 | 0 |
| 336 | G_Fusiform-3-R | 0 | 2 | 0 | 0 |
| 337 | G_Fusiform-4-L | 2 | 0 | 1 | 2 |
| 338 | G_Fusiform-4-R | 0 | 3 | 0 | 0 |
| 339 | G_Fusiform-5-L | 0 | 0 | 1 | 1 |
| 340 | G_Fusiform-5-R | 0 | 2 | 1 | 1 |
| 341 | G_Fusiform-6-L | 2 | 2 | 1 | 1 |
| 342 | G_Fusiform-6-R | 1 | 2 | 0 | 0 |
| 343 | G_Fusiform-7-L | 1 | 0 | 2 | 1 |
| 344 | G_Fusiform-7-R | 0 | 3 | 0 | 1 |
| 345 | N_Amygdala-1-L | 0 | 1 | 0 | 0 |
| 346 | N_Amygdala-1-R | 0 | 0 | 0 | 1 |
| 347 | N_Caudate-1-L | 0 | 1 | 0 | 1 |
| 348 | N_Caudate-1-R | 0 | 1 | 0 | 2 |
| 349 | N_Caudate-2-L | 1 | 2 | 0 | 1 |
| 350 | N_Caudate-2-R | 0 | 1 | 1 | 2 |
| 351 | N_Caudate-3-L | 0 | 3 | 0 | 1 |
| 352 | N_Caudate-3-R | 1 | 1 | 0 | 2 |
| 353 | N_Caudate-4-L | 0 | 2 | 0 | 2 |
| 354 | N_Caudate-4-R | 0 | 2 | 0 | 2 |
| 355 | N_Caudate-5-L | 0 | 2 | 0 | 1 |
| 356 | N_Caudate-5-R | 0 | 1 | 0 | 1 |
| 357 | N_Caudate-6-L | 0 | 1 | 0 | 1 |
| 358 | N_Caudate-6-R | 0 | 1 | 0 | 0 |
| 359 | N_Caudate-7-L | 0 | 0 | 0 | 0 |
| 360 | N_Caudate-7-R | 0 | 0 | 0 | 0 |
| 361 | N_Pallidum-1-L | 0 | 0 | 0 | 0 |
| 362 | N_Pallidum-1-R | 0 | 0 | 0 | 0 |
| 363 | N_Putamen-2-L | 0 | 0 | 0 | 0 |
| 364 | N_Putamen-2-R | 0 | 0 | 0 | 1 |
| 365 | N_Putamen-3-L | 0 | 1 | 0 | 0 |
| 366 | N_Putamen-3-R | 0 | 0 | 0 | 0 |
| 367 | N_Thalamus-1-L | 0 | 0 | 0 | 0 |
| 368 | N_Thalamus-1-R | 0 | 0 | 0 | 1 |
| 369 | N_Thalamus-2-L | 1 | 1 | 0 | 0 |
| 370 | N_Thalamus-2-R | 0 | 0 | 0 | 1 |
| 371 | N_Thalamus-3-L | 0 | 1 | 0 | 0 |
| 372 | N_Thalamus-3-R | 0 | 0 | 0 | 2 |
| 373 | N_Thalamus-4-L | 0 | 2 | 0 | 2 |
| 374 | N_Thalamus-4-R | 1 | 2 | 0 | 0 |
| 375 | N_Thalamus-5-L | 0 | 0 | 0 | 0 |
| 376 | N_Thalamus-5-R | 0 | 0 | 0 | 0 |
| 377 | N_Thalamus-6-L | 0 | 0 | 0 | 0 |
| 378 | N_Thalamus-6-R | 0 | 0 | 0 | 0 |
| 379 | N_Thalamus-7-L | 0 | 0 | 0 | 0 |
| 380 | N_Thalamus-7-R | 0 | 0 | 0 | 0 |
| 381 | N_Thalamus-8-L | 0 | 0 | 0 | 0 |
| 382 | N_Thalamus-8-R | 1 | 0 | 0 | 0 |
| 383 | N_Thalamus-9- L | 0 | 0 | 0 | 0 |
| 384 | N_Thalamus-9-R | 0 | 0 | 0 | 0 |
