# Supplementary material for: Functional network topology in drug resistant and well-controlled idiopathic generalized epilepsy: a resting state functional MRI study
Source: Brain Commun. 2021 Aug 26;3(3):fcab196. doi: 10.1093/braincomms/fcab196 (PMC8417840; doi:10.1093/braincomms/fcab196)
Supplement: fcab196_Supplementary_Data [file Original Submission - Author's Response.pdf › Original Submission - Author's Response.pdf]

Response to reviewers

We thank each of the reviewers for their insightful and constructive comments. We have revised the manuscript to incorporate the suggestions and believe that the paper has been strengthened and as such will be of greater benefit to readers of Brain Communications. Below is a response to each individual comment (in blue text).

Reviewer(s)' Comments to Author:

Reviewer: 1

Comments to the Author
The authors present findings from network correlation and graph theoretical analysis of resting state fMRI in 35 patients with IGE and 35 controls, with the IGE separated into controlled, seizure free for a year and not seizure free. They found differences between IGE and controls but no difference according to seizure control.
The study and hypothesis (that differences in brain network topology will differ according to degree of seizure control) is reasonable - however, the problem is that many other potentially important variables are not considered.

Whilst sample size may have been an issue the heterogeneity of the epilepsy group may have also been a problem.
Grouping all IGE together is reasonable, on the basis that there may be a common underlying network differences, however some recognition and discussion of potential limitation of this, rather than sub-syndrome groupings, JME, JAE, GTCSO should be made.

This has now been discussed as a potential limitation with reference to a relevant paper.

Details of medication, and daily dose should be given in table 2, seizure frequency and recency for each seizure type is also useful, even if not included in statistical analysis, but allows the reader an impression of the cohort, and hence interpretation of the findings.

Details of medication including the doses has now been provided in table 2. Unfortunately, information regarding seizure frequency was not collected and to collect this retrospectively is unlikely to be accurate.

Where were the controls recruited from, community / matched demographics, or not; it is important to state as could be a possible confound in fMRI - eg motion, sleep, familiarity with scanner environment.

Controls were recruited locally from the university and from the general public. None of the participants were familiar with this scanner environment. These details have now been included in the manuscript.

Motion parameters should be compared between groups for any systematic differences. Where there any a priori criteria to exclude any participants based on the extent of motion?

Motion parameters have now been compared between groups (there was no difference between groups). There were a priori criteria to exclude participants depending on extent of movement- this has now been detailed in the methods section.

The authors note in the discussion the possibility of IEDs as a confound, due to absence of concurrent EEG (it should be mentioned that whilst ideal concurrent EEG would add considerably to the time, complexity and cost of the study), however details of recent or past clinical EEG can give a clue as to the 'IED burden' in patients – and if the same cohort in the authors recently published paper details on EEG could be considered.

Some details regarding clinical EEG findings have been added to table 2 in the discussion, we have now mentioned that concurrent EEG would be ideal.

No assessment of possible sleep in the scanner was made? Could be done by post scan questionnaire? Possible exclusion if clearly reports into deep sleep.

Whilst we appreciate that this would have been useful data to collect, we feel that retrospectively asking this now would be inaccurate due to the time period that has lapsed (e.g. some of the controls were scanned in 2016). However, participants were instructed to stay awake and look at a white fixation cross on a black background. Although we did not directly assess sleep, the radiographers were instructing throughout scanning and waiting for answers before and after functional MRI, as they had to be awake to see the cross hair. We have now included this detail in the manuscript.

The fMRI lacks concurrent physiological measures that are now commonly used to regress physiological confounds ie heart rate, respiratory belt, other respiratory measures, and the pre-processing doesn't show any approach to these.

The detail regarding our approach has now been made clear in the manuscript.

Page 3, line 46 "Edges may be weighted according to the strength of the signal." Which signal? Better phrased as strength of correlation (of BOLD signal fluctuations) between at specified nodes?

This has been clarified and rephrased as advised.

Page 4 line 17 – now in press. What were the differences that were found in the EEG study? Was it the same cohort?

This information has been added in and the reference for the now published paper has been included. It was a different cohort of patients (also added into the manuscript).

Page 6, line 39 - 40, what is meant by physiologically relevant connections? ?versus noise? what was the rationale for the chosen thresholds?

This has been clarified and the rationale for the chosen. threshold has been expanded upon

Figure 3 – are these nodes that differ statistically? Or different but not surviving multiple comparisons? Including an outline of statistical test and main findings in figure legend would help.

This has been clarified and the statistical tests on which this figure was based have been referred to

Figure 2 subplot labels were blurred – assume better hires version available

Yes, higher resolution images have been sent with the re-submission.

Supplementary data 2 needs to be better presented that the 'raw' excel file. Data would be better tabulated, titled and with short narrative, including details of the FDR test (rationale for why this was chosen and why q value of 0.8 given in the methods)?

This data has been tabulated as suggested. The q value typo has been corrected- a value of 0.1 was used, as per convention.

Reviewer: 2

Comments to the Author
Summary:

This study presents results regarding changes in fMRI-derived resting-state functional networks in idiopathic generalised epilepsy, as compared to healthy controls. Graph-theoretical measures are derived to quantify possible differences in network topology. Strong evidence is presented for an increase in the average node degree in IGE, and there are also significant increases in either the characteristic path length or the average betweenness centrality, depending on whether negative correlations are taken into account or not.

In general, this article is well written, and the results are well discussed. I do however, have some reservations regarding nodal differences and the thresholding, as well as some other minor issues.

Comments:

Major:

1. My main concern with this study is that results for nodal metrics are lend too much importance, because they can only be interpreted as false (and therefore random) positives after correction for multiple comparison.

(a) One cannot speak of significant differences, when after correction for multiple testing the null hypothesis cannot be rejected. Rather, one should more carefully refer to 'trends' in specific nodes (e.g. in the subsection 'Nodal outcome metrics')

(2) Figure 3 essentially presents false positives, for the above-stated reasons. I would therefore reconsider if this figure needs to be included, since there are no conclusions that can be drawn from it, unless a more elaborate analysis is performed. For example, is there an unusually large number (more than what would occur randomly) of nodes in which both node degree and betweenness centrality differ?

We thank the reviewer for raising these important points (a and 2). The wording of this section has been altered to more carefully describe the interpretation of the results that did not survive correction for multiple comparisons.

After further consideration, we feel that inclusion of figure 3 remains useful. The issue of correcting for multiple comparisons, particularly in an exploratory study, is not straightforward and it is recommended by some that correction is not always necessary (e.g. Althouse 2016, Adjust for Multiple Comparisons? It's Not That Simple. DOI: 10.1016/j.athoracsur.2015.11.024. Feise 2002, https://link.springer.com/article/10.1186/1471-2288-2-8).

However, we agree that the wording of the description should be altered and have also now included effect sizes in the results table. This should enable readers to more easily use their own judgement as to the meaning of these particular results.

(3) p.7, l. 51/52: Here you state that the FDR was controlled with q=0.8. I believe this is a typo, as q adjusts (approximately) the FDR, and in the literature values of q=0.05 or q=0.1 are used.

This has been corrected. A q value of 0.1 was used.

(4) p.12 l.3/4 'Similar to the present study, previous fMRI connectivity studies have found widespread locations of specific nodes that display altered connectivity in IGE, …' This study has not identified any specific nodes with altered connectivity. This sentence should be rephrased.

The phrase "similar to the present study" has been removed as advised.

2. It is common practice to threshold networks before subjecting them to further analysis. However, thresholding can significantly alter the network structure, and the thresholds are often chosen randomly. I would therefore suggest to test how sensitive the results are with respect to this choice, for instance by choosing a few different threshold values, or by removing the thresholding altogether. I assume the results are robust with respect to the choice of threshold value, but some further analysis, or at least a short discussion of this problem, would lend more credence to the results.

Further analysis has now been performed to test a range of thresholds and the problem of threshold has been discussed.

Minor:

1. p.6 l. 32/33: I do not understand why the Fisher transform was used. It is a monotone mapping of correlations from [-1,1] onto [-infinity,infinity], which should not have any bearing on the results.

This refers to z = arctanh(r), not to computation of a standard score.

2. Kruskall —> Kruskal (typo in the manuscript)

This has been rectified.

3. Figure 2: figure and axis labels should be increased in size, currently they are hard to read.

This has been altered

4. The Bonferroni correction factor should be explicitly stated in the text (I expect it is 12, 6 graph theory measures, times 2 network types).

The correction factor has now been stated.

5. I suggest to refer to 'node strength' by 'node degree', which is more common.

The term node strength was used as is it is a fully connected graph. It has now been explained that this is an equivalent measure to mean degree in a network that is fully connected (in the corresponding table and when first mentioned in the manuscript).

6. ll. 40-42: 'valproate, lamotrigine or valproate' (what is the third?)

This has been corrected. The second valproate has been replaced by levetiracetam

7. The discussion on p.13, ll. 10-20 suggest that seizures are over-reported. However, the study by Cook et al. (Lancet Neurology 2013, 12(6): 563-571) and esp. Fig. 4 therein suggest that seizures tend to be under-reported.

The fact that under-reporting of seizures may occur has now been discussed and the paper mentioned has been referenced

Reviewer: 3

Comments to the Author
The study presents an analysis of resting-state fMRI networks from people with IGE and healthy controls. Within the IGE cohort, drug-resistance and seizure freedom are considered as two separate sub-groups. A number of standard graph metrics are computed and statistical comparisons carried out between the (sub)groups. The paper is well written, and the questions and data contribute to the ongoing efforts of understanding the importance of network alterations with respect to the likelihood of seizures. As it stands, however, I think there are a number of issues that would need to be addressed before publication.

Major
Power
The authors summarize a number of limitations and potential confounders in the discussion; including the sample size. I think that a more rigorous and thorough discussion is necessary to solidify that these findings are not just the result of an under-powered study (see also my comment on full details of the multiple comparisons later on). For example, were sample size carried out beforehand, when constructing the statistical analysis plan?

Further discussion on potential underpowering has now been included in the discussion section (including a comment about the limitation of a power calculation in the absence of established population values for network metrics) and correction for multiple comparisons has been further detailed.

Confounder
It is not clear to me whether the entire IGE cohort is on AEDs; and furthermore, whether drug-load should not be considered as a potential confounder? Additionally, should seizure severity not be considered as a confounder?

Drug information has now been included in table 2. The potential for differences in drug load has been added to the discussion on limitations.

Dichotomization
I think it is important to provide more context to the distinction between drug-resistant and well-controlled subgroups. If the distinction between them is purely clinical (i.e. 1 year of no (self)reported seizures), but the actual phenomenon itself may be on a "continuous spectrum", then there are potential problems with imposing an "arbitrary" threshold (there are numerous papers and commentaries that have shown the risks associated with this, see for example MacCallum et al., 2002, On the practice of dichotomization of quantitative variables). Alternatively, why not consider time from last (self-reported) seizure as a variable of interest?

Some discussion on this interesting point has been added.

Minor
Though it's mentioned, the exact details of the multiple comparison corrections carried out are not included. It would be better if the full details were included, even if it was as a supplementary table.

Further detail about correction for multiple comparisons has now been added.

In general, I think reporting effect sizes together with the p-values would strengthen the overall presentation of the results. Some discussion on p-values could be beneficial as well, as at times the lack of statistical significance seems to be used to imply there is evidence of no effect.

This has now been included.

How robust are the results with respect to the specific choice of threshold for the networks? Again, this could go in the supplementary materials, but it would be worth contrasting a number of choices, say 0, 0.125, 0.25 and 0.375?).

Further analysis has now been performed to test these threshold levels.

The resolution in Figure 2 is not great: it is quite hard to read the captions and see the individual data points in clear detail. It would be great if the figure could be provided in much crisper resolution.

The font has now been made larger and the image saved in the highest possible resolution.

Editorial Office comments:

-Please supply a graphical abstract. A graphical abstract is a visual representation of the central finding or methodology of the paper. It will be displayed in the table of contents. Authors should strive to make the graphical abstract informative, interesting, visually appealing, and straightforward (no caption needed, no abbreviations except gene names). Please upload as a separate high resolution image file (tiff preferred). The technical requirements for the graphical abstracts are:

--size of the submitted image: 1200 pixels square at 300dpi

--font of the text: Arial 12-16 point (smaller fonts will not be legible online)

-Please add an Abbreviated Summary (up to 50 words) of your paper that captures the main purpose and conclusions of the work. The summary should state the main results written in the third person ('[1st author surname] et al. report that …') and conclusion. This summary will be used in the contents list online and should not contain any abbreviations other than those on the accepted abbreviations list and accepted gene/protein names, making it accessible to the non-specialist. Please upload it as a Word document. Allowed abbreviations: AIDS; ANOVA; AMPA; ATP; A,T,C,G; CNS; CSF; CT; DNA; ECG; EEG; EMG; GABA; HIV; MRI; NMDA; PET; PCR; RNA.

-Please include a paragraph about data availability in the 'Materials and Methods' section. We strongly encourage data sharing either as supplementary information or uploads to repositories which you can link in the paper.

- To comply with our ethical standards, the appropriate checklist must be included (see Reporting Guideline section within 'Instructions to authors').
