# Supplementary material for: Functional network topology in drug resistant and well-controlled idiopathic generalized epilepsy: a resting state functional MRI study
Source: Brain Commun. 2021 Aug 26;3(3):fcab196. doi: 10.1093/braincomms/fcab196 (PMC8417840; doi:10.1093/braincomms/fcab196)
Supplement: fcab196_Supplementary_Data [file Original Submission – Decision Letter.pdf › Original Submission – Decision Letter.pdf]

Original Submission – Decision Letter

18-Feb-2021

Dear Dr. Pegg,

Thank you for your recent submission BRAINCOM-2020-365 entitled "Functional network topology in drug resistant and well-controlled Idiopathic Generalised Epilepsy: A resting-state fMRI study" to Brain Communications.

Your manuscript has been reviewed, and while the reviewers and editorial team appreciate many aspects of the paper, they raise some concerns that you will need to address in order for the paper to be considered for publication.

If you can address these concerns, I invite you to submit a revised manuscript and a response to each of the reviewers' comments. The reviewer(s)' comments are included at the bottom of this message.

In addition to the reviewers' comments, please note our editorial comments added below to comply with our requirements for presentation of scientific data at Brain Communications.

Please format your revised manuscript according to our Instructions to Authors https://academic.oup.com/braincomms/pages/General_Instructions – a summary of key points is attached to help with this.

To revise your manuscript, click the link below or log into https://mc.manuscriptcentral.com/braincom and enter your Author Centre, where you will find your manuscript title listed under "Manuscripts with Decisions." Under "Actions," click on "Create a Revision." Your manuscript number has been appended to denote a revision.

*** PLEASE NOTE: This is a two-step process. After clicking on the link, you will be directed to a webpage to confirm. ***

https://mc.manuscriptcentral.com/braincom?URL_MASK=622df0769e8c4beda11a3c4942f8fb66

Please upload a revised manuscript with changes tracked with track changes or in coloured text.

When submitting your revised manuscript, you will be asked to respond to the reviewer(s) comments. Please be as specific as possible in your response to all of the comments.

The editorial team at Brain Communications aims to increase transparency in scientific publishing and to demonstrate the value that peer review brings to the scientific process. Please therefore consider whether you are willing to have reviewer(s)' comments on your paper and your response published as supplementary data with the paper if it is accepted. This is entirely optional and will only be done if both the authors and reviewers agree. While submitting your revision there will be an opportunity to review or update the answer you gave previously, if necessary.

IMPORTANT: Your original files are available to you when you upload your revised manuscript. Please delete any redundant files before completing the submission.

Because we are trying to facilitate timely publication of manuscripts submitted to Brain Communications, we would appreciate your revised manuscript as soon as possible. If it is not possible for you to resubmit within 3 months, the option to submit a revision will expire, and we would then consider your revised paper as a new submission. However, if you need a little more time, please get in touch and we can extend this deadline for you.

Once again, thank you for submitting your manuscript to Brain Communications and I look forward to receiving your revision.

Yours sincerely,

Prof. Richard Chin
Editor, Brain Communications

Reviewer(s)' Comments to Author:

Reviewer: 1 (Khalid Hamandi)

Comments to the Author
The authors present findings from network correlation and graph theoretical analysis of resting state fMRI in 35 patients with IGE and 35 controls, with the IGE separated into controlled, seizure free for a year and not seizure free. They found differences between IGE and controls but no difference according to seizure control.
The study and hypothesis (that differences in brain network topology will differ according to degree of seizure control) is reasonable – however, the problem is that many other potentially important variables are not considered. Whilst sample size may have been an issue the heterogeneity of the epilepsy group may have also been a problem.
Grouping all IGE together is reasonable, on the basis that there may be a common underlying network differences, however some recognition and discussion of potential limitation of this, rather than sub-syndrome groupings, JME, JAE, GTCSO should be made.
Details of medication, and daily dose should be given in table 2, seizure frequency and recency for each seizure type is also useful, even if not included in statistical analysis, but allows the reader an impression of the cohort, and hence interpretation of the findings.
Where were the controls recruited from, community / matched demographics, or not; it is important to state as could be a possible confound in fMRI – eg motion, sleep, familiarity with scanner environment.
Motion parameters should be compared between groups for any systematic differences. Where there any a priori criteria to exclude any participants based on the extent of motion?
The authors note in the discussion the possibility of IEDs as a confound, due to absence of concurrent EEG (it should be mentioned that whilst ideal concurrent EEG would add considerably to the time, complexity and cost of the study), however details of recent or past clinical EEG can give a clue as to the 'IED burden' in patients – and if the same cohort in the authors recently published paper details on EEG could be considered.
No assessment of possible sleep in the scanner was made? Could be done by post scan questionnaire? Possible exclusion if clearly reports into deep sleep.
The fMRI lacks concurrent physiological measures that are now commonly used to regress physiological confounds ie heart rate, respiratory belt, other respiratory measures, and the pre-processing doesn't show any approach to these.
Page 3, line 46 "Edges may be weighted according to the strength of the signal." Which signal? Better phrased as strength of correlation (of BOLD signal fluctuations) between at specified nodes?
Page 4 line 17 – now in press? What were the differences that were found in the EEG study? Was it the same cohort?
Page 6, line 39 - 40, what is meant by physiologically relevant connections? ?versus noise? what was the rationale for the chosen thresholds?
Figure 3 – are these nodes that differ statistically? Or different but not surviving multiple comparisons? Including an outline of statistical test and main findings in figure legend would help.
Figure 2 subplot labels were blurred – assume better hires version available
Supplementary data 2 needs to be better presented that the 'raw' excel file. Data would be better tabulated, titled and with short narrative, including details of the FDR test (rationale for why this was chosen and why q value of 0.8 given in the methods)?

Reviewer: 2

Comments to the Author
Summary:

This study presents results regarding changes in fMRI-derived resting-state functional networks in idiopathic generalised epilepsy, as compared to healthy controls. Graph-theoretical measures are derived to quantify possible differences in network topology. Strong evidence is presented for an increase in the average node degree in IGE, and there are also significant increases in either the characteristic path length or the average betweenness centrality, depending on whether negative correlations are taken into account or not.

In general, this article is well written, and the results are well discussed. I do however, have some reservations regarding nodal differences and the thresholding, as well as some other minor issues.

Comments:

Major:

1. My main concern with this study is that results for nodal metrics are lend too much importance, because they can only be interpreted as false (and therefore random) positives after correction for multiple comparison.

(a) One cannot speak of significant differences, when after correction for multiple testing the null

hypothesis cannot be rejected. Rather, one should more carefully refer to 'trends' in specific nodes (e.g. in the subsection 'Nodal outcome metrics')

(2) Figure 3 essentially presents false positives, for the above-stated reasons. I would therefore reconsider if this figure needs to be included, since there are no conclusions that can be drawn from it, unless a more elaborate analysis is performed. For example, is there an unusually large number (more than what would occur randomly) of nodes in which both node degree and betweenness centrality differ?

(3) p.7, l. 51/52: Here you state that the FDR was controlled with q=0.8. I believe this is a typo, as q adjusts (approximately) the FDR, and in the literature values of q=0.05 or q=0.1 are used.

(4) p.12 l.3/4 'Similar to the present study, previous fMRI connectivity studies have found widespread locations of specific nodes that display altered connectivity in IGE, …' This study has not identified any specific nodes with altered connectivity. This sentence should be rephrased.

2. It is common practice to threshold networks before subjecting them to further analysis. However, thresholding can significantly alter the network structure, and the thresholds are often chosen randomly. I would therefore suggest to test how sensitive the results are with respect to this choice, for instance by choosing a few different threshold values, or by removing the thresholding altogether. I assume the results are robust with respect to the choice of threshold value, but some further analysis, or at least a short discussion of this problem, would lend more credence to the results.

Minor:

1. p.6 l. 32/33: I do not understand why the Fisher transform was used. It is a monotone mapping of correlations from [-1,1] onto [-infinity,infinity], which should not have any bearing on the results.

2. Kruskall —> Kruskal (typo in the manuscript)

3. Figure 2: figure and axis labels should be increased in size, currently they are hard to read.

4. The Bonferroni correction factor should be explicitly stated in the text (I expect it is 12, 6 graph theory measures, times 2 network types).

5. I suggest to refer to 'node strength' by 'node degree', which is more common.

6. ll. 40-42: 'valproate, lamotrigine or valproate' (what is the third?)

7. The discussion on p.13, ll. 10-20 suggest that seizures are over-reported. However, the study by Cook et al. (Lancet Neurology 2013, 12(6): 563-571) and esp. Fig. 4 therein suggest that seizures tend to be under-reported.

Reviewer: 3

Comments to the Author
The study presents an analysis of resting-state fMRI networks from people with IGE and healthy controls. Within the IGE cohort, drug-resistance and seizure freedom are considered as two separate sub-groups. A number of standard graph metrics are computed and statistical comparisons carried out between the (sub)groups. The paper is well written, and the questions and data contribute to the ongoing efforts of understanding the importance of network alterations with respect to the likelihood of seizures. As it stands, however, I think there are a number of issues that would need to be addressed before publication.

Major
Power
The authors summarize a number of limitations and potential confounders in the discussion; including the sample size. I think that a more rigorous and thorough discussion is necessary to solidify that these findings are not just the result of an under-powered study (see also my comment on full details of the multiple comparisons later on). For example, were sample size carried out beforehand, when constructing the statistical analysis plan?

Confounder
It is not clear to me whether the entire IGE cohort is on AEDs; and furthermore, whether drug-load should not be considered as a potential confounder? Additionally, should seizure severity not be considered as a confounder?

Dichotomization
I think it is important to provide more context to the distinction between drug-resistant and well-controlled subgroups. If the distinction between them is purely clinical (i.e. 1 year of no (self)reported seizures), but the actual phenomenon itself may be on a “continuous spectrum”, then there are potential problems with imposing an “arbitrary” threshold (there are numerous papers and commentaries that have shown the risks associated with this, see for example MacCallum et al., 2002, On the practice of dichotomization of quantitative variables). Alternatively, why not consider time from last (self-reported) seizure as a variable of interest?

Minor
Though it’s mentioned, the exact details of the multiple comparison corrections carried out are not included. It would be better if the full details were included, even if it was as a supplementary table.

In general, I think reporting effect sizes together with the p-values would strengthen the overall presentation of the results. Some discussion on p-values could be beneficial as well, as at times the lack of statistical significance seems to be used to imply there is evidence of no effect.

How robust are the results with respect to the specific choice of threshold for the networks? Again, this could go in the supplementary materials, but it would be worth contrasting a number of choices, say 0, 0.125, 0.25 and 0.375?).

The resolution in Figure 2 is not great: it is quite hard to read the captions and see the individual data points in clear detail. It would be great if the figure could be provided in much crisper resolution.

Editorial Office comments:

-Please supply a graphical abstract. A graphical abstract is a visual representation of the central finding or methodology of the paper. It will be displayed in the table of contents. Authors should strive to make the graphical abstract informative, interesting, visually appealing, and straightforward (no caption needed, no abbreviations except gene names). Please upload as a separate high resolution image file (tiff preferred). The technical requirements for the graphical abstracts are:
  --size of the submitted image: 1200 pixels square at 300dpi
  --font of the text: Arial 12-16 point (smaller fonts will not be legible online)

-Please add an Abbreviated Summary (up to 50 words) of your paper that captures the main purpose and conclusions of the work. The summary should state the main results written in the third person (‘[1st author surname] et al. report that …’) and conclusion. This summary will be used in the contents list online and should not contain any abbreviations other than those on the accepted abbreviations list and accepted gene/protein names, making it accessible to the non-specialist. Please upload it as a Word document. Allowed abbreviations: AIDS; ANOVA; AMPA; ATP; A,T,C,G; CNS; CSF; CT; DNA; ECG; EEG; EMG; GABA; HIV; MRI; NMDA; PET; PCR; RNA.

-Please include a paragraph about data availability in the 'Materials and Methods' section. We strongly encourage data sharing either as supplementary information or uploads to repositories which you can link in the paper.

- To comply with our ethical standards, the appropriate checklist must be included (see Reporting Guideline section within 'Instructions to authors').
